# Verazine Biosynthesis from Simple Sugars in Engineered *Saccharomyces cerevisiae*

**DOI:** 10.1016/j.ymben.2024.07.011

**Published:** 2024-07-27

**Authors:** Peter H. Winegar, Graham A. Hudson, Luisa B. Dell, Maria C. T. Astolfi, James Reed, Rocky D. Payet, Hugo C. J. Ombredane, Anthony T. Iavarone, Yan Chen, Jennifer W. Gin, Christopher J. Petzold, Anne E. Osbourn, Jay D. Keasling

**Affiliations:** 1 Joint BioEnergy Institute, Lawrence Berkeley National Laboratory, Emeryville, CA 94608, USA; 2 Biological Systems and Engineering, Lawrence Berkeley National Laboratory, Berkeley, CA 94720, USA; 3 California Institute for Quantitative Biosciences (QB3 Institute), University of California, Berkeley, Berkeley, CA 94720, USA; 4 Department of Chemical and Biomolecular Engineering and Department of Bioengineering, University of California, Berkeley, Berkeley, CA 94720, USA; 5 The Novo Nordisk Foundation Center for Biosustainability, Technical University Denmark, Kemitorvet, Building 220, Kongens Lyngby 2800, Denmark; 6 John Innes Centre, Norwich Research Park, Norwich NR4 7UH, UK

**Keywords:** Verazine, Steroidal Alkaloid, Natural Product, Microbial Biosynthesis, Metabolic Engineering, Synthetic Biology

## Abstract

Steroidal alkaloids are FDA-approved drugs (*e.g*., Zytiga) and promising drug candidates/leads (*e.g*., cyclopamine); yet many of the ≥ 697 known steroidal alkaloid natural products remain underutilized as drugs because it can be challenging to scale their biosynthesis in their producing organisms. Cyclopamine is a steroidal alkaloid produced by corn lily (*Veratrum* spp.) plants, and it is an inhibitor of the Hedgehog (Hh) signaling pathway. Therefore, cyclopamine is an important drug candidate/lead to treat human diseases that are associated with dysregulated Hh signaling, such as basal cell carcinoma and acute myeloid leukemia. Cyclopamine and its semi-synthetic derivatives have been studied in (pre)clinical trials as Hh inhibitor-based drugs. However, challenges in scaling the production of cyclopamine have slowed efforts to improve its efficacy and safety profile through (bio)synthetic derivatization, often limiting drug development to synthetic analogs of cyclopamine such as the FDA-approved drugs Odomzo, Daurismo, and Erivedge. If a platform for the scalable and sustainable production of cyclopamine were established, then its (bio)synthetic derivatization, clinical development, and, ultimately, widespread distribution could be accelerated. Ongoing efforts to achieve this goal include the biosynthesis of cyclopamine in *Veratrum* plant cell culture and the semi-/total chemical synthesis of cyclopamine. Herein, this work advances efforts towards a promising future approach: the biosynthesis of cyclopamine in engineered microorganisms. We completed the heterologous microbial production of verazine (biosynthetic precursor to cyclopamine) from simple sugars (*i.e*., glucose and galactose) in engineered *Saccharomyces cerevisiae* (*S. cerevisiae*) through the inducible upregulation of the native yeast mevalonate and lanosterol biosynthetic pathways, diversion of biosynthetic flux from ergosterol (*i.e*., native sterol in *S. cerevisiae*) to cholesterol (*i.e*., biosynthetic precursor to verazine), and expression of a refactored five-step verazine biosynthetic pathway. The engineered *S. cerevisiae* strain that produced verazine contains eight heterologous enzymes sourced from seven different species. Importantly, *S. cerevisiae*-produced verazine was indistinguishable via liquid chromatography-mass spectrometry from both a commercial standard (*Veratrum* spp. plant-produced) and *Nicotiana benthamiana*-produced verazine. To the best of our knowledge, this is the first report describing the heterologous production of a steroidal alkaloid in an engineered yeast. Verazine production was ultimately increased through design-build-test-learn cycles to a final titer of 83 ± 3 µg/L (4.1 ± 0.1 µg/g DCW). Together, this research lays the groundwork for future microbial biosynthesis of cyclopamine, (bio)synthetic derivatives of cyclopamine, and other steroidal alkaloid natural products.

## Graphical abstract

**Graphical Abstract:**
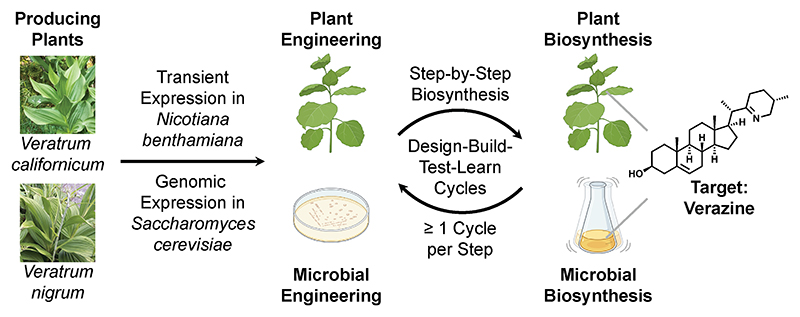

## Abbreviations

AML: acute myeloid leukemia
ARS: autonomously replicating sequence
BCC: basal cell carcinoma
bp: base pair
CM medium: complete minimal medium
CoA: coenzyme A
CPR: cytochrome P450 reductase
CYP450: cytochrome P450
DC: detergent-compatible
DEEMM: diethyl ethoxymethylenemalonate
DIA: data-independent acquisition
DMAPP: dimethylallyl diphosphate
DNA: deoxyribonucleic acid
ER: endoplasmic reticulum
*E. coli*
: *Escherichia coli*
ESI: electrospray ionization
FDR: false discovery rate
GABA: γ-aminobutyric acid
G418 sulfate: Geneticin
GC-MS: gas chromatography-mass spectrometry
Hh: Hedgehog
ICE: Inventory of Composable Elements
IPP: isopentenyl diphosphate
JBEI: Joint BioEnergy Institute
LC-MS: liquid chromatography-mass spectrometry
kbp: kilobase pair
LLS: longest linear sequence
MSBP: membrane steroid-binding protein
*N. benthamiana*
: *Nicotiana benthamiana*
NCBI: National Center for Biotechnology Information
NGS: next-generation sequencing
OD_600_: optical density at 600 nm
PCR: polymerase chain reaction
QB3: California Institute for Quantitative Biosciences
rcf: relative centrifugal force
RP-HPLC: reverse-phase-high-performance liquid chromatography
*S. cerevisiae*
: *Saccharomyces cerevisiae*
SDS: sodium dodecyl sulfate
SOC medium: super optimal medium with catabolite repression
SRA: sequence read archive
TCEP: tris 2-(carboxyethyl)phosphine
TMD: transmembrane domain
UHPLC: ultra-high performance liquid chromatography
UV-vis: ultraviolet-visible
*V. californicum*
: Veratrum californicum
*nigrum*
: *Veratrum nigrum*
w/v%: weight/volume %
YP: yeast extract peptone
YPD medium: yeast extract peptone dextrose medium

## 1 Introduction

Steroidal alkaloids are a class of bioactive (*e.g*., anti-inflammatory, analgesic, antimicrobial, antithrombotic, antiandrogenic, and antiarrhythmic)^1–4^ molecules that include an FDA-approved drug (*i.e*., abiraterone acetate [Zytiga]^5^) and promising drug candidates/leads (*e.g*., cyclopamine^6^). A library of ≥ 697 steroidal alkaloids have been discovered in lily (*Liliaceae*), nightshade (*Solanaceae*), dogbane (*Apocynaceae*), and box (*Buxaceae*) spp. plants; amphibians; and marine organisms.^7^ However, many steroidal alkaloid natural products remain underutilized as drugs because it can be time intensive and environmentally damaging to extract them from their producing organisms at scales that are sufficient for therapeutic study and use. Ongoing efforts to address this challenge have produced select steroidal alkaloid natural products (and new-to-nature derivatives) via biosynthesis in engineered plants,^8–11^ biosynthesis in engineered plant cell culture,^12^ and semi-/total chemical synthesis.^13^ One promising future approach to achieve the scalable and sustainable production of steroidal alkaloid natural products (and new-to-nature derivatives) is biosynthesis in engineered microorganisms. Microbial biosynthesis has been used to successfully produce a wide range of other natural products (*e.g*., vinblastine,^14^ cannabinols,^15^ QS-21,^16^ opioids,^17^ diosgenin,^18^ steroids,^19^ saponins,^20^ etc.) and new-to-nature derivatives (*e.g*., halogenated natural product analogs^21,22^), often from simple sugars.^23–30^ In one prominent example, *Saccharomyces cerevisiae* (*S. cerevisiae*) was engineered to biosynthesize artemisinic acid, a product that can be converted to artemisinin by semi-synthesis.^31,32^ This technology was utilized by Sanofi to produce 51 million artemisinin treatments, contributing to the low current cost of artemisinin-containing anti-malarial combination therapies.^33^ If achieved, the microbial biosynthesis of steroidal alkaloid natural products could drive their (bio)synthetic derivatization, clinical development, and, ultimately, widespread distribution.

Cyclopamine is a steroidal alkaloid produced by corn lily (*Veratrum* spp.) plants (*e.g*., *Veratrum californicum* [*V. californicum*] and *Veratrum nigrum* [*V. nigrum*])^34–36^ that binds antagonistically to the G-coupled protein receptor Smoothened, inhibiting signal transduction in the Hedgehog (Hh) pathway.^37^ While regulated Hh pathway signaling controls tissue patterning (during embryogenesis) and tissue regeneration (after embryogenesis), dysregulated Hh pathway signaling is associated with birth defects (during embryogenesis; *e.g*., cyclopia and polydactyly) and diseases (after embryogenesis; *e.g*., basal cell carcinoma [BCC] and acute myeloid leukemia [AML]).^38,39^ Therefore, Hh inhibitors like cyclopamine are promising drug candidates/leads to treat diseases associated with dysregulated Hh signaling like BCC and AML. Cyclopamine was studied in pre-clinical trials to treat psoriasis^40^ and saridegib (a semi-synthetic derivative of cyclopamine also known as IPI-926 or patidegib) remains under investigation in clinical trials to treat BCC (*i.e*., ClinicalTrials.gov ID: NCT06050122).^41,42^ However, the scalable and sustainable production of cyclopamine remains challenging, slowing efforts to improve its efficacy and safety profile through (bio)synthetic derivatization and often limiting drug development to synthetic analogs of cyclopamine (*e.g*., sonidegib [Odomzo], glasdegib [Daurismo], and vismodegib [Erivedge], which are FDA-approved drugs to treat BCC, AML, and BCC, respectively).^43^ Most cyclopamine produced for use in drugs is isolated from the roots and rhizomes of wild *V. californicum* plants using benzene-based extractions (~1.3 g/kg dried plant material).^44^ This production strategy is complicated by the greater than seven years required between harvests of wild *V. californicum* plants^45^ and the variation in cyclopamine yields between individual plants, growth locations, and seasons.^46^ Successful efforts have established alternative platforms for cyclopamine production at small scales including cyclopamine biosynthesis in plant cell cultures of *V. californicum*,^47–49^ semi-synthesis from dehydroepiandrosterone (20-step, 1% overall yield),^50^ and convergent total synthesis (16-step longest linear sequence [LLS], 1.4% overall yield;^51^ 23-step LLS, 0.2% overall yield;^52^ and 13-step LLS, 6.2% overall yield^53^). One promising future approach to achieve the scalable and sustainable production of cyclopamine is biosynthesis in engineered microorganisms.

The cyclopamine biosynthetic precursor verazine (**1**) is a steroidal alkaloid^35,54^ produced in corn lily (*Veratrum*),^55^ death camas (*Zygadenus*),^56^ and nightshade (*Solanum*)^57^ spp. plants. Verazine (**1**) exhibits weak bioactivity, with anti-inflammatory^58^ and anti-fungal properties (*e.g*., IC_50_ values of 6.2 µg/mL for *Candida albicans*,^59^ 3.1 µg/mL for *Trichophyton rubrum*,^59^ and 6.25 µg/mL for *S. cerevisiae* Sc7^60^). In *V. californicum*^61^ and *V. nigrum*^62^ plants, verazine (**1**) is biosynthesized from cholesterol (**6**) through a five-step biosynthetic pathway (*i.e*., C22 hydroxylation of cholesterol [**6**], C26 oxidation of 22-hydroxycholesterol [**7**], C26 transamination of 22-hydroxycholesterol-26-al [**8**], C22 oxidation of 22-hydroxy-26-aminocholesterol [**10**], and spontaneous cyclization of 22-keto-26-aminocholesterol [**11**]; Supplementary Figure S1). Several strategies have successfully produced verazine (**1**) at small scales, including semi-synthesis from diosgenin (15-step, yield not reported),^63^ biosynthesis via heterologous pathway expression in *Spodoptera frugiperda* Sf9 insect cell culture (yield not reported),^61^ biosynthesis via heterologous pathway expression in *Camelina sativa* seed (50 pg/mg seed),^64^ and biosynthesis via transient pathway expression in *Nicotiana benthamiana* (5.11 μg/g dry cell weight [DCW]).^62^ Together, these studies enable efforts to establish the heterologous biosynthesis of verazine (**1**) in *S. cerevisiae*.

**Figure 1:**
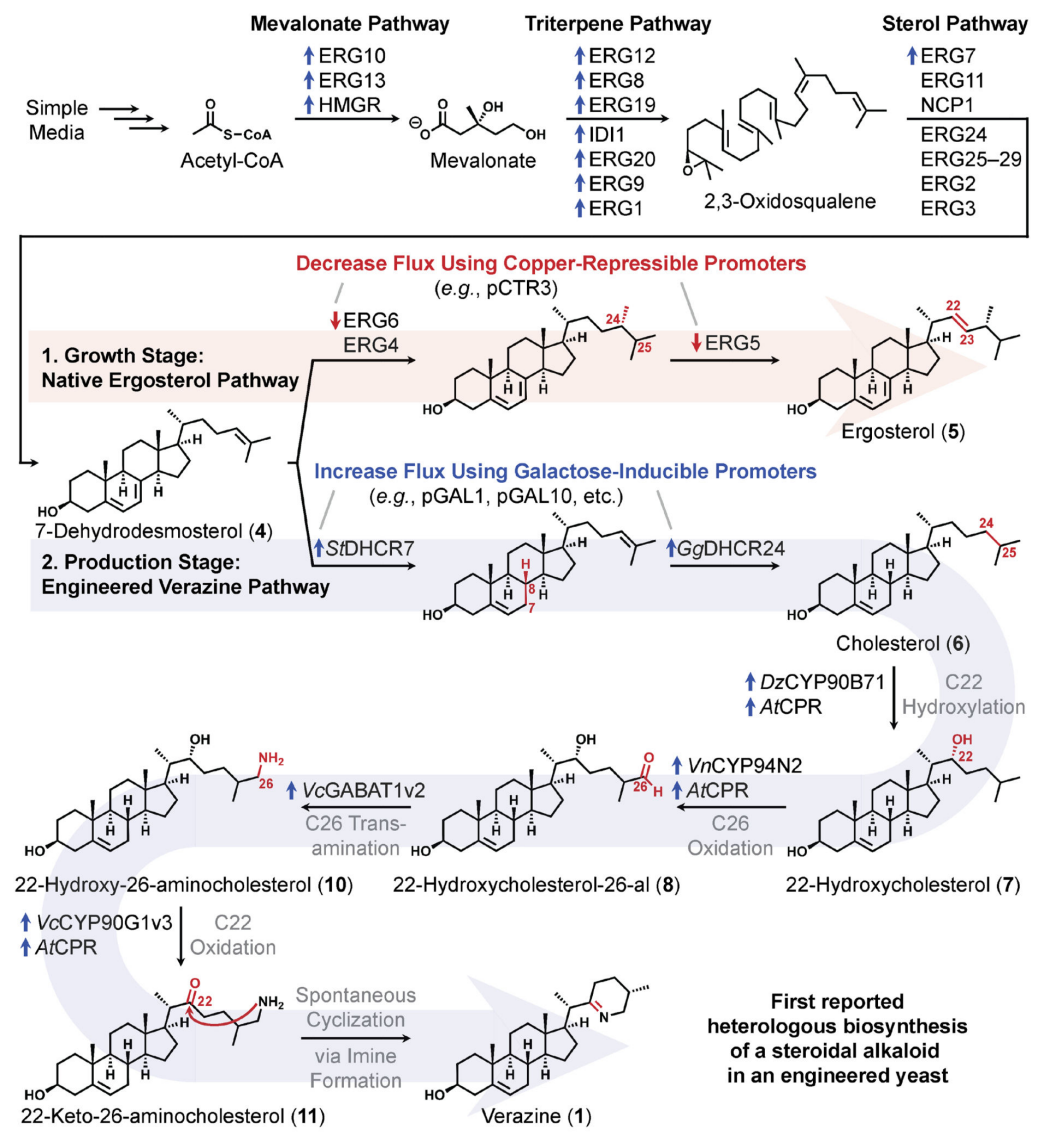
*S. cerevisiae* was engineered to overproduce sterols through inducible overexpression of enzymes in the mevalonate and lanosterol (**3**) biosynthetic pathways (top). An inducible shift from ergosterol (**5**) to cholesterol (**6**) biosynthesis enabled a two-stage production strategy with separate growth (red arrow) and production (blue arrow) stages (middle). Verazine (**1**) was biosynthesized from cholesterol (**6**) through inducible expression of an engineered biosynthetic pathway (bottom). In this figure, blue arrows (oriented upwards) next to gene names indicate genes that are under control of galactose-inducible promoters and red arrows (oriented downwards) next to gene names indicate genes that are under control of copper-repressible promoters.

Herein, the heterologous biosynthesis of verazine (**1**), a biosynthetic precursor to cyclopamine, was refactored in *S. cerevisiae*. Verazine (**1**) was biosynthesized from the simple sugars glucose and galactose in a strain of *S. cerevisiae* that was engineered with inducible upregulation of the native yeast mevalonate and lanosterol biosynthetic pathways, diversion of biosynthetic flux from ergosterol (**5**, *i.e*., native sterol from *S. cerevisiae*) to cholesterol (**6**, *i.e*., biosynthetic precursor to verazine [**1**]), and expression of a refactored verazine (**1**) biosynthetic pathway containing eight heterologous proteins sourced from seven different species (Figure 1). Yeast strains and fermentation conditions were then engineered through design-build-test-learn cycles to increase verazine (**1**) production titers to 83 ± 3 µg/L (4.1 ± 0.1 µg/g DCW). Together, this research lays the groundwork for future microbial biosynthesis of cyclopamine, (bio)synthetic derivatives of cyclopamine, and other steroidal alkaloid natural products.

## 2 Materials and Methods

### 2.1 Source of chemicals and DNA

Chemicals used in this study as analytical standards, reagents, and medium components (Supplementary Table S1) and chemicals produced in this study (Supplementary Table S2) are detailed in the Supplementary Information. Genes used in this study (Supplementary Table S3) were amplified via polymerase chain reaction (PCR) from the genomic DNA of CEN.PK2-1C-derived *S. cerevisiae* (https://www.yeastgenome.org/) or purchased from Integrated DNA Technologies as gBlocks that were codon-optimized for *S. cerevisiae* (https://www.idtdna.com/CodonOpt). DNA primers for PCR amplification were purchased from Integrated DNA Technologies (Supplementary Tables S4 and S5).

### 2.2 Gene identification from transcriptomics data

To identify *V. nigrum* homologs to reported genes for verazine (**1**) biosynthesis in *V. californicum*,^61^ genes were mined from publicly available transcriptome data deposited in the National Center for Biotechnology Information (NCBI). Sequence Read Archive (SRA) accession SRR22875544 was chosen for transcriptome mining because this SRA was derived from *V. nigrum* seedling roots (a site of cyclopamine production) and had the largest number of sequenced nucleobases.

The SRA accession SRR22875544 was downloaded from NCBI and converted to FASTQ format using fasterq-dump from the NCBI SRA-Toolkit. FASTQ files were then assembled using Trinity v2.14.0^65^ using default options. The resultant assembled reads were compiled into a BLAST database using makeblastdb and the BLAST database was subsequently queried for homologs via a translated nucleotide BLAST (tblastn) using the *V. californicum* biosynthetic enzymes as query sequences.^66^ Multiple sequence alignments were performed using CLUSTAL Omega 1.2.4.^67^

### 2.3 Plasmid construction and verification

All plasmids used in this study (Supplementary Table S6) were obtained from Joint BioEnergy Institute’s (JBEI) Inventory of Composable Elements (ICE)^68^ or constructed according to the following protocol. Genes (Supplementary Table S3), promoters (*i.e*., pGAL1, pGAL2, pGAL7, and pGAL10), terminators (*i.e*., tADH1 and tCYC1), and the pESC-URA vector backbone were amplified by polymerase chain reaction (PCR) on an Applied Biosciences VeritiPro 96-Well Thermal Cycler (Thermo Fisher Scientific). Primers were designed to amplify genes utilized in this work (Supplementary Table S4). PCR was performed with the Q5 Hot Start High-Fidelity DNA Polymerase (New England Biolabs, M0493S), Q5 Reaction Buffer (New England Biolabs, B9027S), dNTP mix (Thermo Scientific, R0192) with the manufacturer’s protocol modified to add 1 M betaine to the reaction mixture. PCR products and the GeneRuler 1 kb Plus DNA Ladder (Thermo Scientific, SM1332) were mixed with 6× purple gel loading dye without sodium dodecyl sulfate (SDS, New England Biolabs, B7025S) and loaded into a 1.1 weight/volume % (w/v%) agarose gel stained with GelGreen nucleic acid stain. The agarose gel was run in an Owl Easycast Electrophoresis System (Thomas Scientific) using an Owl EC-105 Power Supply (Thermo Scientific) at 125 V for 30 min. Gels were imaged on an UVP GelSolo Imager (Analytik Jena). Bands corresponding to target DNA amplicons were cut out of the gel and DNA was extracted from the gel bands using the Zymoclean Gel DNA Recovery Kit (Zymo Research, D4002). Concentrations of purified DNA amplicons were measured using an ND-1000 Spectrophotometer (NanoDrop). Next, pESC-URA plasmids were constructed from DNA amplicons via Gibson assembly performed using the NEBuilder HiFi DNA Assembly Master Mix (New England Biolabs, E2621L) according to the manufacturer’s protocol. Subsequently, 50 µL of chemically competent XL1-blue *Escherichia coli* (*E. coli*, QB3-Berkeley) was transformed with 2 µL of products from the Gibson assembly. This mixture was incubated on ice for 30 min, heat shocked in a 42 °C, water bath for 45 s, and incubated on ice for 2 min. Transformed *E. coli* were rescued with 950 µL of Super Optimal medium with Catabolite repression (SOC medium, New England Biolabs, B9020S), incubated at 37 °C for 1 h with shaking at 300 revolutions per minute (rpm), plated onto 1× LB medium agar plates containing 100 µg/mL carbenicillin (Teknova, L1010), and incubated at 37 °C overnight. Individual *E. coli* colonies were selected from the plates and used to inoculate 5-mL of 1× LB medium containing 100 µg/mL carbenicillin. These liquid cell cultures were incubated at 37 °C overnight with shaking at 200 rpm. Glycerol stocks for each liquid cell culture were saved and stored at -80 °C. Next, plasmids were extracted from liquid cell cultures using the QIAprep Spin Miniprep Kit (Qiagen, 27104). Successful plasmid construction was confirmed by whole plasmid next-generation sequencing (NGS) with Plasmidsaurus Labs. For each plasmid constructed in this study (Supplementary Table S6), the plasmid DNA sequence can be found on JBEI’s ICE and the plasmid-transformed *E. coli* strain can be requested from the JBEI’s ICE at https://public-registry.jbei.org/folders/860 (accession numbers can be found in Supplementary Table S6).^68^

### 2.4 *S. cerevisiae* strain construction and verification

All *S. cerevisiae* strains used in this study (Supplementary Table S7) were obtained from JBEI’s ICE^68^ or constructed according to one of the following protocols.

For *S. cerevisiae* transformations involving deletion of native genes and replacing them with heterologous genes, selection based on the KanMX marker was used.^69^ Competent *S. cerevisiae* was transformed following the lithium acetate/single-stranded carrier DNA/polyethylene glycol transformation method^70^ with an equimolar mixture of four DNA fragments: a fragment homologous to ~1 kilobase pairs (kbps) upstream of the integration site, a fragment containing the KanMX cassette, a fragment containing the DNA for integration (2,000 ng), and a fragment homologous to ~1 kbps downstream of the integration site with 30–60 base pairs (bps) of overlap between fragments. Transformed *S. cerevisiae* was rescued with 950 µL of 1× yeast extract peptone dextrose medium (YPD medium), incubated at 30 °C overnight with shaking at 300 rpm, plated onto a 1× YPD medium agar plate containing 300 mg/L of Geneticin (G418 sulfate), and incubated at 30 °C for three to seven days. Colony screen PCR using DNA primers outside the homologous DNA region from the transformation was performed on individual, well-spaced colonies from the selection plate. Colony screen PCR products were mixed with 6× purple gel loading dye without SDS and characterized by running them on a 1.1 w/v% agarose gel stained containing GelRed nucleic acid stain and imaged to identify potential successful integrations. Successful gene integration was then confirmed by purifying colony screen PCR products by the QIAquick PCR Purification Kit (Qiagen, 28104) and submitting them for NGS with Plasmidsaurus Labs. A colony with a successful gene integration was used to inoculate 1× YPD and these liquid cell cultures were incubated at 30 °C for two days with 200 rpm shaking and then a glycerol stock of the *S. cerevisiae* strain was then saved and stored at -80 °C. To cure the KanMX marker, competent *S. cerevisiae* was transformed following the lithium acetate/single-stranded carrier DNA/polyethylene glycol transformation method^70^ with an equimolar mixture of two DNA fragments: a fragment homologous to ~1 kbps upstream of the KanMX marker (2,000 ng) and a fragment homologous to ~1 kbps downstream of the integration site with 30–60 bps of overlap between fragments as well as a pCUT-URA-KanMX plasmid (400 ng, Supplementary Table S6). Following transformation, *S. cerevisiae* was plated onto a complete minimal medium (CM medium) minus uracil agar plates with glucose (Teknova, C3080) and incubated at 30 °C for three to seven days. Colony screen PCR using DNA primers outside the homologous DNA region from the transformation was performed on individual, well-spaced colonies from the selection plate. Colony screen PCR products were mixed with 6× purple gel loading dye without SDS and characterized by running them on a 1.1 w/v% agarose gel stained containing GelRed nucleic acid stain and imaged to identify potential successful integrations. Successful gene integration was then confirmed by purifying colony screen PCR products by the QIAquick PCR Purification Kit and submitting them for NGS with Plasmidsaurus Labs. To select for the removal of the pCUT-URA-KanMX plasmid from the yeast strain, a colony with a successful gene integration was used to inoculate 1× YPD medium, this liquid cell culture was incubated at 30 °C overnight with 200 rpm shaking, this liquid cell cultures were plated onto a CM medium agar plate with glucose and 5-fluoroorotic acid (5-FOA, Teknova, F2344), and these plates were incubated at 30 °C for three to seven days. A colony from this plate was used to inoculate 1× YPD medium, this liquid cell culture was incubated at 30 °C for two days with 200 rpm shaking, and glycerol stock of the *S. cerevisiae* strain was saved and stored at -80 °C.

A published protocol was used to integrate genes into genetically stable, autonomously replicating sequences (ARSs).^71^ Competent *S. cerevisiae* was transformed following the lithium acetate/single-stranded carrier DNA/polyethylene glycol transformation method^70^ with an equimolar mixture of three or four DNA fragments: a fragment homologous to ~1 kbps upstream of the integration site, one or two fragments containing the DNA for integration (2,000 ng), and a fragment homologous to ~1 kbps downstream of the integration site with 30–60 bps of overlap between fragments as well as a corresponding CRISPR/Cas9 pCUT-URA-ARS plasmid (400 ng) from the CRISPR/Cas9 toolkit (Supplementary Table S6).^71^ Following transformation, *S. cerevisiae* was plated onto a CM medium minus uracil agar plate with glucose (Teknova, C3080) and incubated at 30 °C for three to seven days. Colony screen PCR using DNA primers outside the homologous DNA region from the transformation was performed on individual, well-spaced colonies from the selection plate. Colony screened PCR products were mixed with 6× purple gel loading dye without SDS and characterized by running them on a 1.1 w/v% agarose gel stained containing GelRed nucleic acid stain and imaged to identify potential successful integrations. Successful gene integration was then confirmed by purifying colony screen PCR products by the QIAquick PCR Purification Kit (Qiagen, 28104) and submitting them for NGS with Plasmidsaurus Labs. To select for the removal of the pCUT-URA-ARS plasmid from the yeast strain, a colony with a successful gene integration was used to inoculate 1× YPD medium, this liquid cell culture was incubated at 30 °C overnight with 200 rpm shaking, this liquid cell cultures were plated onto a CM medium agar plate with glucose and 5-FOA (Teknova, F2344), and these plates were incubated at 30 °C for three to seven days. A colony from this plate integration was used to inoculate 1× YPD medium, this liquid cell culture was incubated at 30 °C for two days with 200 rpm shaking, and a glycerol stock of the *S. cerevisiae* strain was saved and stored at -80 °C.

To transform *S. cerevisiae* for plasmid-based protein expression, competent *S. cerevisiae* was transformed following the lithium acetate/single-stranded carrier DNA/polyethylene glycol transformation method^70^ with pESC-URA plasmid (300 ng, Supplementary Table S6) containing the promoters, genes, and terminators to be used for protein expression. Following transformation, *S. cerevisiae* was plated onto a CM medium minus uracil agar plate with glucose (Teknova, C3080) and incubated at 30 °C for three to seven days. An individual, well-spaced colony was used to inoculate 1× CM minus uracil with glucose (2 w/v%) medium, this liquid cell culture was incubated at 30 °C for two days with 200 rpm shaking, and a glycerol stock of the *S. cerevisiae* strain was saved and stored at -80 °C.

For each *S. cerevisiae* strain constructed in this study (Supplementary Table S7), the relevant genomic DNA sequence can be found on JBEI’s ICE and the *S. cerevisiae* strain can be requested from the JBEI’s ICE at https://public-registry.jbei.org/folders/860 (accession numbers for each strain can be found in Supplementary Table S7).^68^

### 2.5 Transient expression in *N. benthamiana* and preparation of leaf extracts

Genes for transient expression in *Nicotiana benthamiana* (*N. benthamiana*) were first cloned from *V. californicum. V. californicum* root tissue was homogenized to a fine powder under liquid nitrogen, and then 50 mg was transferred to an RNase-free microcentrifuge tube. To this, 600 µl of lysis buffer (4 M guanidine isothiocyanate, 200 mM sodium acetate, 25 mM EDTA, 2.5% PVP-40, 1% β-mercaptoethanol) and 60 µl of 20% sarcosyl (Sigma, L9150) was added, followed by a 10 min incubation at 70 °C with shaking. Lysate was directly applied to a QIAshredder spin column (Qiagen, 79656) and centrifuged at 18,000 relative centrifugal force (rcf) for 2 min, and the supernatant was then transferred to a fresh tube. 0.5 vol of ethanol was added and then the sample was transferred to an RNeasy Mini spin column (Qiagen, 74104), after which it was centrifuged for 15 s at 8,000 rcf. DNase treatment was performed on the column and RNA concentration was eluted as per manufacturer’s instructions. Purity and concentration of RNA was ascertained by Nanodrop, and then cDNA was prepared using SuperScriptTM III (ThermoFisher, 18080-093), also according to the manufacturer’s instructions.

Genes utilized in *N. benthamiana* include BLAST-identified verazine synthesis genes (*i.e*., CYP90B27v1, CYP94N1v2, GABATv2, CYP90G1v3) from *V. californicum* and genes used to amplify cholesterol biosynthesis (i.e., SSR2, SMO3, 3βHSD2, CPI, CYP51, C-14-R, 8,7-SI, SMO4, C5DS2, 7DR2, and SD) from *Solanum lycopersicum*^72^ (Supplementary Table S9). Primers were designed to amplify each gene (Supplementary Table S5). These primers were flanked with attB sites compatible with Gateway cloning. Genes were amplified by PCR using iProof High-Fidelity DNA Polymerase (Bio-Rad, 1725330) either from prepared root cDNA (for *V. californicum* genes) or from cDNA prepared from tomato leaves (for the cholesterol biosynthesis genes). After confirming a single, correct product on an agarose gel, PCR-products were cleaned up using QIAquick PCR purification kit (Qiagen, 28104), and cloned into pDONR207 using BP Clonase II (ThermoFisher, 11789020). Entry clones were confirmed by Sanger sequencing (Eurofins) and then mobilized into the binary expression vector, pEAQ-*HT*-DEST1, using LR Clonase (ThermoFisher, 11791100). Expression constructs were transformed into *Agrobacterium tumefaciens* strain LBA4404. The transformed cells were inoculated into 10 mL LB media with antibiotics and cultured overnight at 28 °C. Following this, cells were pelleted by centrifugation at 4,000 rcf for 10 min and the pellet was resuspended in 5 mL MMA buffer (10 mM MgCl2, 10 mM MES buffer, 150 μM acetosyringone). Relevant combinations of the resuspended cells were made depending on the desired verazine intermediate (1 mL each) and the total volume was made to 10 mL. In all combinations, the tomato cholesterol biosynthesis genes were co-infiltrated to enhance cholesterol content in *N. benthamiana* and subsequent accumulation of verazine and precursors. Leaves of 5-week-old *N. benthamiana* plants were infiltrated with the necessary combinations and left under greenhouse conditions for 5 days before harvesting. Harvested leaf material was lyophilized and 10 mg dry leaf material was ground using tungsten beads in a Spex Geno/Grinder at 1,000 rpm. The powdered leaf material was resuspended in 500 μL ethanol and shaken for 20 min at 1,000 rpm and 40°C in a Thermo Mixer C (Eppendorf). The extract was centrifuged for 5 min at 20,000 rcf to pellet leaf material and the supernatant was filtered using a 0.22 μm filter mini column (Norgen). The filtered extract was subsequently used for LC-MS.

### 2.6 Liquid cell culture of *S. cerevisiae* for metabolite production

A scale of 5 mL was used for liquid cell cultures for metabolite production. Glycerol stocks for *S. cerevisiae* strains were streaked onto 1× YPD plates (Teknova, Y1000) (or CM medium minus uracil agar plate with glucose [Teknova, C3080] for plasmid-based protein expression), and these plates were incubated at 30 °C for one to two days. An individual, well-spaced colony was used to inoculate 5 mL of 1× yeast extract peptone (YP) with glucose (2 w/v%) medium (or 1× CM minus uracil with glucose [2 w/v%] medium for plasmid-based protein expression) in a 25 × 150 mm glass culture tube. This liquid cell culture was incubated at 30 °C for two days with 200 rpm shaking. Cells were then pelleted by centrifugation (3,000 rcf, 5 min), the supernatant was discarded, and the cells were resuspended in 5 mL of 1× YP with galactose (2 w/v%), copper (II) sulfate (200 µM),^73^ and γ-aminobutyric acid (GABA, 100 mM) (or 1× CM minus uracil with galactose [2 w/v%], copper (II) sulfate [200 µM], and GABA [100 mM] medium for plasmid-based protein expression) depending on experimental conditions and this liquid cell culture was incubated at 30 °C for one to three days with 200 rpm shaking. The final optical density at 600 nm (OD_600_) for this culture was measured using a SpectraMax absorbance plate reader and ultraviolet-visible (UV-vis) spectrophotometer (Molecular Devices, PLUS 384).

### 2.7 Dry cell weight measurement

To measure dry cell weight (DCW), *S. cerevisiae* liquid culture with a known OD_600_ was added to a massed tube. Next, cells were pelleted by centrifugation (13,000 rcf, 5 min) and the supernatant was discarded. Cells were dried on a FreeZone 6 lyophilizer (Labconco) overnight and the Eppendorf tube was massed again. DCW was measured as the observed increase in mass of the Eppendorf tube containing the dried *S. cerevisiae* cells. The value of DCW/OD_600_ agreed well with the reported value of 0.644 ± 0.025 g DCW/L/OD_600_,^74^ so this value was used throughout this work.

### 2.8 Gas chromatography-mass spectrometry (GC-MS) characterization

At the conclusion of a liquid cell culture for metabolite production, 2 mL of liquid cell culture was pelleted by centrifugation (13,000 rcf, 5 min), the supernatant was discarded, and the cells were resuspended in 600 µL of 50:50 40 w/v% potassium hydroxide:100% ethanol and ~100 µL of 0.5 mm glass beads (Next Advance, GB05) were added to the cell pellets. Cells were lysed by shaking (30 Hz, 5 min) on a MM400 mixer mill (Retsch). Products from this pathway were extracted from lysed cells by adding 1 mL of GC-MS grade *n*-hexane and shaking (30 Hz, 5 min) on a MM400 mixer mill (Retsch). The mixture was centrifuged (18,000 rcf, 5 min) and the organic phase was transferred to an LC-MS vial (Agilent). Next, the sample was analyzed on a 6980 GC-5973 MSD (Agilent) or an Intuvo 9000 GC-5977B MSD (Agilent) using a HP-5MS column (Agilent, 19091S-433) and a previously reported heating program.^75^

### 2.9 Liquid chromatography-mass spectrometry (LC-MS) characterization

At the conclusion of a liquid cell culture for metabolite production, 1 mL of liquid cell culture was pelleted by centrifugation (13,000 rcf, 5 min). After removing and discarding the supernatant, 1 mL of 80:20 methanol:nanopure water and ~100 µL of 0.5 mm glass beads (Next Advance, GB05) were added to the cell pellets. Cells were lysed by shaking (30 Hz, 5 min) on a MM400 mixer mill (Retsch). The insoluble fractions of cell lysates were pelleted by centrifugation (18,000 rcf, 5 min) and the supernatant was transferred to a new Eppendorf tube (1.5 mL). Cell lysates were dried at 30 °C under vacuum on a CentriVap benchtop concentrator (Labconco, 7810010). The dried cell lysate was resuspended in 50 µL of 100% methanol with three alternating rounds of vortexing for 10 s and sonicating for 10 min. The resuspended mixture was centrifuged (18,000 rcf, 5 min) to pellet insoluble debris and the supernatant was transferred to an LC-MS vial with a vial insert (Agilent). The aldehyde on 22-hydroxycholesterol-26-al (**8**) was derivatized into (**9**) by mixing equal volumes of the resuspended methanol extract and 2 mg/mL *o*-(carboxymethyl)hydroxylamine hemihydrochloride, and this mixture was left to react at ambient temperature for > 3 h.^76^

For LC-MS characterization, samples were run on a 1260 Infinity II LC/MSD iQ (Agilent) or a 1260 Infinity II LC/MSD XT (Agilent) LC-MS (for most LC-MS characterization) or a 1200 series LC (Agilent)-LTQ-Orbitrap-XL MS with an electrospray ionization source (Thermo Fisher Scientific) LC-MS (for high resolution LC-MS characterization). All LC-MS were run using an analytical EC UHPLC Nucleodur C18 Htec 1.8 µm 100×2 mm column (Macherey Nagel, 760306.20) at room temperature, an injection volume of 5 µL, and a gradient from LC-MS grade water with 0.1% formic acid (buffer A) to LC-MS grade acetonitrile with 0.1% formic acid (buffer B) (*i.e*., 85:15 buffer A:buffer B for 1.5 min, a gradient from 85:15 buffer A:buffer B to 40:60 buffer A:buffer B for 24.5 min, a gradient from 40:60 buffer A:buffer B to 0:100 buffer A:buffer B for 0.5 min, and 0:100 buffer A:buffer B for 6.5 min).

For high-resolution LC-MS characterization, samples were analyzed using a 1200 series LC system (Agilent) that was connected in line with an LTQ-Orbitrap-XL mass spectrometer equipped with an electrospray ionization (ESI) source (Thermo Fisher Scientific). The LC system contained the following modules: G1322A solvent degasser, G1311A quaternary pump, G1316A thermostatted column compartment, and G1329A autosampler unit (Agilent). The LC column compartment was equipped with an Ultra C18 column (length: 150 mm, inner diameter: 2.1 mm, particle size: 3 µm, catalog number: 9174362, Restek). Acetonitrile, formic acid (Optima LC-MS grade, 99.9% minimum, Fisher) and water purified to a resistivity of 18.2 MΩ cm (at 25 °C) using a Milli-Q Gradient ultrapure water purification system (Millipore, Billerica, MA) were used to prepare LC mobile phase solvents. Mobile phase solvent A was 99.9% water/0.1% formic acid and mobile phase solvent B was 99.9% acetonitrile/0.1% formic acid (vol/vol). The elution program consisted of isocratic flow at 15% (vol/vol) B for 1.5 min, a linear gradient to 60% B over 24.5 min, a linear gradient to 99.5% B over 0.5 min, isocratic flow at 99.5% B for 6.5 min, a linear gradient to 15% B over 2 min, and isocratic flow at 15% B for 20 min, at a flow rate of 150 µL/min. The column compartment was maintained at 40 °C and the sample injection volume was 2 µL. External mass calibration was performed using the Pierce LTQ ESI positive ion calibration solution (catalog number 88322, Thermo Fisher Scientific). Full-scan, high-resolution mass spectra were acquired in the positive ion mode over the range of mass-to-charge ratio (*m*/*z*) = 100 to 1,000 using the Orbitrap mass analyzer, in profile format, with a mass resolution setting of 100,000 (at *m*/*z* = 400, measured at full width at half-maximum peak height). Data acquisition and analysis were performed using Xcalibur software (version 2.0.7, Thermo Fisher Scientific).

### 2.10 Confocal microscopy to characterize protein localization in *S. cerevisiae*

Protein localization experiments were performed using plasmid-based expression of the protein of interest fused to mCherry. Glycerol stocks for *S. cerevisiae* strains containing the relevant plasmid were streaked onto a CM medium minus uracil agar plate with glucose (Teknova, C3080) and these plates were incubated at 30 °C for one to two days. Next, a colony from this plate integration was used to inoculate 5 mL of 1× CM minus uracil with glucose (2 w/v%) medium for plasmid-based protein expression). This liquid cell culture was incubated at 30 °C for two days with 200 rpm shaking. Cells were then pelleted by centrifugation (3,000 rcf, 5 min), the supernatant was discarded, and the cells were resuspended in 5 mL of 1× CM minus uracil with galactose (4 w/v%) and this liquid cell culture was incubated at 30 °C for 18 h with 200 rpm shaking. Next, 250 µL of liquid cell culture was pelleted, the supernatant was removed, the cell pellet was resuspended in 50 µL of nuclease-free water, and this mixture was imaged on a LSM710 Confocal Microscope (Zeiss). First, 5 μL of the liquid cell culture resuspended in water was added between a glass slide and a cover slip. Samples were imaged using a 100× objective in the mCherry channel (λ_excitation_ = 580 nm and λ_emission_ = 585–696 nm) using identical image acquisition parameters (*e.g*., laser power, master gain, pinhole size, scan speed, offset) and image processing (*i.e*., Zen 3.3 Blue Edition [Zeiss]) for each sample.

### 2.11 Proteomics analysis of protein expression levels in *S. cerevisiae*

Liquid cell cultures of *S. cerevisiae* were prepared as described in section 2.6. Cells were harvested and stored at -80 °C until further processing. Protein was extracted from cell pellets and tryptic peptides were prepared by following an established proteomic sample preparation protocol.^77^ Briefly, cell pellets were resuspended in Qiagen P2 Lysis Buffer (Qiagen) to promote cell lysis. Proteins were precipitated with addition of 1 mM NaCl and 4 x vol acetone, followed by two additional washes with 80% acetone in water. The recovered protein pellet was homogenized by pipetting mixing with 100 mM ammonium bicarbonate in 20% methanol. Protein concentration was determined by the detergent-compatible (DC) protein assay (BioRad). Protein reduction was accomplished using 5 mM tris 2-(carboxyethyl)phosphine (TCEP) for 30 min at room temperature, and alkylation was performed with 10 mM iodoacetamide (final concentration) for 30 min at room temperature in the dark. Overnight digestion with trypsin was accomplished with a 1:50 trypsin:total protein ratio. The resulting peptide samples were analyzed on an Agilent 1290 UHPLC system coupled to a Thermo Scientific Orbitrap Exploris 480 mass spectrometer for discovery proteomics.^78^ Briefly, peptide samples were loaded onto an Ascentis® ES-C18 Column (Sigma Aldrich) and were eluted from the column by using a 10 min gradient from 98% solvent A (water with 0.1% formic acid) and 2% solvent B (acetonitrile with 0.1% formic acid) to 65% solvent A and 35% solvent B. Eluting peptides were introduced to the mass spectrometer operating in positive-ion mode and were measured in data-independent acquisition (DIA) mode with a duty cycle of 3 survey scans from *m/z* 380 to *m/z* 985 and 45 MS2 scans with precursor isolation width of 13.5 *m/z* to cover the mass range. DIA raw data files were analyzed by an integrated software suite DIA-NN.^79^ The database used in the DIA-NN search (library-free mode) is *S. cerevisiae* latest UniProt proteome FASTA sequences plus the protein sequences of the heterologous proteins and common proteomic contaminants. DIA-NN determines mass tolerances automatically based on first pass analysis of the samples with automated determination of optimal mass accuracies. The retention time extraction window was determined individually for all MS runs analyzed via the automated optimization procedure implemented in DIA-NN. Protein inference was enabled, and the quantification strategy was set to Robust LC = High Accuracy. Output main DIA-NN reports were filtered with a global false discovery rate (FDR) = 0.01 on both the precursor level and protein group level. The Top3 method, which is the average MS signal response of the three most intense tryptic peptides of each identified protein, was used to plot the quantity of the targeted proteins in the samples.^80,81^

### 2.12 Analytical determination of residual GABA in fermentation media

Residual GABA in post-fermentation media was measured using diethyl ethoxymethylenemalonate (DEEMM) derivatization and reverse-phase high-performance liquid chromatography (RP-HPLC) as described by Kim et al.^82^ with minor modifications. First, 3 μL of post-fermentation media supernatant or GABA standard (with concentrations ranging from 100 μM to 100 mM) was mixed with 180 μL sodium borate (pH 9.0), 60 μL methanol, 74 μL water, and 3 μL of 400 mM DEEMM in methanol (total reaction volume 320 μL) and heated at 70 °C for 2 hours. DEEMM-derivatized samples were analyzed on an Agilent 1260 Infinity II HPLC system equipped with an analytical EC UHPLC Nucleodur C18 Htec 1.8 µm 100×2 mm column (Macherey Nagel, 760306.20) at room temperature, an injection volume of 5 μL, and using LC-MS grade water with 0.1% formic acid (buffer A) and LC-MS grade acetonitrile with 0.1% formic acid (buffer B) with the following gradient: 0 min at 20% B, 2 min at 25% B, 32 min at 60% B, and 40 min at 20% B with a 15 min hold for equilibration between injections. DEEMM-derivatives were analyzed by monitoring at 284 nm and DEEMM-derivatized GABA had a retention time of 6.1 min. The areas under the curves for DEEMM-derivatized GABA standards were used to construct a standard curve and derivatized media samples were interpolated to determine residual GABA concentration.

## 3 Results and Discussion

*S. cerevisiae* is a eukaryotic microorganism with simple, well-established methods for genetic and metabolic engineering; this yeast was chosen to be the heterologous microorganism host for verazine (**1**) biosynthesis. Engineering details for each *S. cerevisiae* strain constructed in this work can be found in Supplementary Table S7. The *S. cerevisiae* strain JWy601 was selected as the starting point for this work because it has been engineered previously to produce high titers of heterologous terpenoids.^83^ JWy601 contains the native mevalonate, triterpene, and sterol biosynthetic pathways from *S. cerevisiae* as well as an additional galactose-inducible copy of the genes encoding the enzymes that convert acetyl-coenzyme A (CoA) to isopentenyl diphosphate (IPP) and dimethylallyl diphosphate (DMAPP) (*i.e*., ERG10, ERG13, HMG1, HMG2, ERG12, ERG8, ERG19, and IDI1; Supplementary Figure S2). Biosynthetic flux in JWy601 can be diverted away from triterpenoid production and towards sesquiterpenoid production using the copper-repressible CTR3 promoter driving expression of its native ERG9 gene.^73^ Since verazine (**1**) is a biosynthetic derivative of triterpenoids, this CTR3 promoter in the strain JWy601 was reverted to the native ERG9 promoter in strain PW-1 via KanMX-based selection followed by removal of the KanMX marker via the CRISPR/Cas9 toolkit.

### 3.1 Inducible shift from ergosterol to cholesterol biosynthesis in *S. cerevisiae*

Sterol biosynthesis is tightly regulated in *S. cerevisiae*^84^ because the molecular structure, composition, and concentration of sterols can impact essential membrane properties (*e.g*., fluidity, permeability, and structure) and protein functions (*e.g*., efflux pumping, cation pumping, and receptor signaling).^85,86^ The endpoint of the native sterol biosynthetic pathway in *S. cerevisiae* is a relatively low titer (≤ 7.8 mg/g DCW) of ergosterol (**5**).^87^ In contrast, cholesterol (**6**) is not produced in native *S. cerevisiae*, but is a major sterol produced by plants^72^ that can be further transformed into bioactive natural products^18,88^ including verazine (**1**)^61,62^ and cyclopamine.^35^ Therefore, engineering *S. cerevisiae* to overproduce cholesterol (**6**) is an important prerequisite for the heterologous microbial production of these molecules.

The final common precursor in ergosterol (**5**) and cholesterol (**6**) biosynthesis is 7-dehydrodesmosterol (**4**). In native *S. cerevisiae*, 7-dehydrodesmosterol (**4**) is converted to ergosterol (**5**) by ERG6 (Δ24-methyltransferase), ERG4 (Δ24(28)-reductase), and ERG5 (22(23)-desaturase); and in cholesterol (**6**)-producing organisms, 7-dehydrodesmosterol (**4**) can be converted to cholesterol (**6**) by DHCR7 (Δ7(8)-reductase) and DHCR24 (Δ24(25)-reductase) (Figure 1, middle). Engineering *S. cerevisiae* by deletion of native genes for ERG6 (and sometimes ERG5) and heterologous expression of *Dr*DHCR7 and *Dr*DHCR24 from *Danio rerio* resulted in cholesterol (**6**) production.^89^ This approach also enabled the biosynthesis of the cholesterol (**6**) derivative diosgenin to be refactored in *S. cerevisiae*.^18^ However, these strains exhibited growth defects. Two strategies that partially solved this challenge were downregulating ergosterol (**5**) biosynthesis instead of removing it in a cholesterol (**6**)-producing strain^90^ as well as optimizing medium and fermentation conditions.^91^

We hypothesized that the growth defect in cholesterol (**6**)-producing *S. cerevisiae* could be avoided using a two-stage fermentation strategy involving a growth stage that utilizes the native ergosterol (**5**) biosynthetic pathway in *S. cerevisiae* to maximize cell mass (Figure 1, middle) and a production stage that utilizes an inducible engineered biosynthetic pathway to biosynthesize cholesterol (**6**) and, ultimately, verazine (**1**) (Figure 1, bottom). To test this hypothesis, the native promoters for ERG5 (the 500 bp upstream of the ERG5 gene were chosen) and ERG6 (the 171 bp upstream of the ERG6 gene were chosen) in strain PW-1 were sequentially replaced with the copper-repressible CTR3 promoter in strains PW-2 and PW-3, respectively. This strain engineering used KanMX-based selection followed by removal of the KanMX marker via the CRISPR/Cas9 toolkit. Next, galactose-inducible copies of the genes encoding the enzymes that convert IPP and DMAPP into lanosterol (**3**) (*i.e*., ERG20, ERG9, ERG1, and ERG7) were integrated into strain PW-3 at YPRCΔ15 using the CRISPR/Cas9 toolkit, creating strain PW-4 (Figure 1, top). The biosynthetic pathway from lanosterol (**3**) to 7-dehydrodesmosterol (**4**) was not engineered because it contains no reported bottlenecks in sterol biosynthesis in native *S. cerevisiae*.^86^ When strain PW-4 was incubated with shaking for 48 h in the growth stage (1× YP + glucose [2% w/v]) and 48 h in the production stage (1× YP + galactose [2% w/v]), it produced squalene (**2**), lanosterol (**3**), and ergosterol (**5**) that had matched gas chromatography-mass spectrometry (GC-MS) retention times and mass spectra as compared to commercial standards (Supplementary Figures S4, S5, and S6). Squalene (**2**), lanosterol (**3**), and ergosterol (**5**) were produced at 180 ± 11 mg/L (10.8 ± 0.6 mg/g DCW), 23 ± 3 mg/L (1.4 ± 0.2 mg/g DCW), and 16 ± 1 mg/L (0.97 ± 0.04 mg/g DCW), respectively (Supplementary Figures S15a-b and S16).

**Figure 2:**
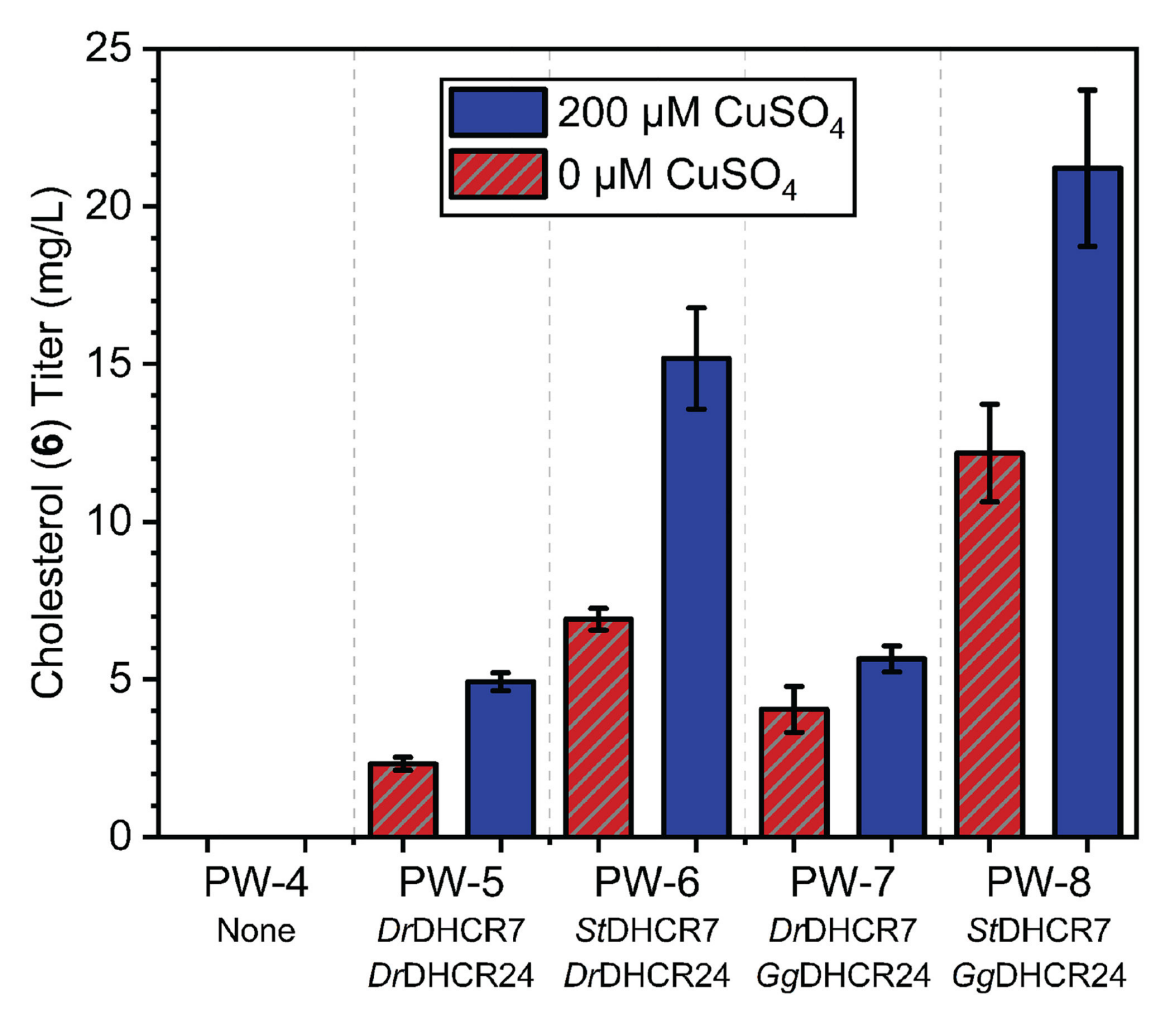
Titers for cholesterol (**6**) in strains PW-4, PW-5, PW-6, PW-7, and PW-8 as measured by GC-MS. The production stage medium contained either 0 µM (dashed red) or 200 µM (blue) copper (II) sulfate.

A similar strategy to a previous report was taken to complete cholesterol (**6**) biosynthesis in *S. cerevisiae*.^90^ Each combination of the cholesterol (**6**)-producing enzymes DHCR7 from *Danio rerio* and *Solanum tuberosum* with DHCR24 from *Danio rerio* and *Gallus gallus* were integrated into strain PW-4 at ARS1021 using the CRISPR/Cas9 toolkit (*i.e*., *Dr*DHCR7 and *Dr*DHCR24 in strain PW-5; *St*DHCR7 and *Dr*DHCR24 in strain PW-6; *Dr*DHCR7 and *Gg*DHCR24 in strain PW-7; and *St*DHCR7 and *Gg*DHCR24 in strain PW-8). These strains were incubated with shaking for 48 h in the growth stage (1× YP + glucose [2% w/v]) and 48 h in the production stage (1× YP + galactose [2% w/v] ± copper (II) sulfate [200 µM]). Cholesterol (**6**) was produced in each strain matched GC-MS retention times and mass spectra as compared to a commercial standard (Supplementary Figure S7). Repression of ERG6 and ERG5 expression during the production stage by including copper (II) sulfate in the production medium increased cholesterol (**6**) titers, with the highest titer of 21 ± 2 mg/L of (1.5 ± 0.2 mg/g DCW) found when *St*DHCR7 and *Gg*DHCR24 were utilized in strain PW-8 (Figure 2; Supplementary Figures S15c).

### 3.2 Refactoring verazine biosynthesis step-by-step in *S. cerevisiae*

**Figure 3:**
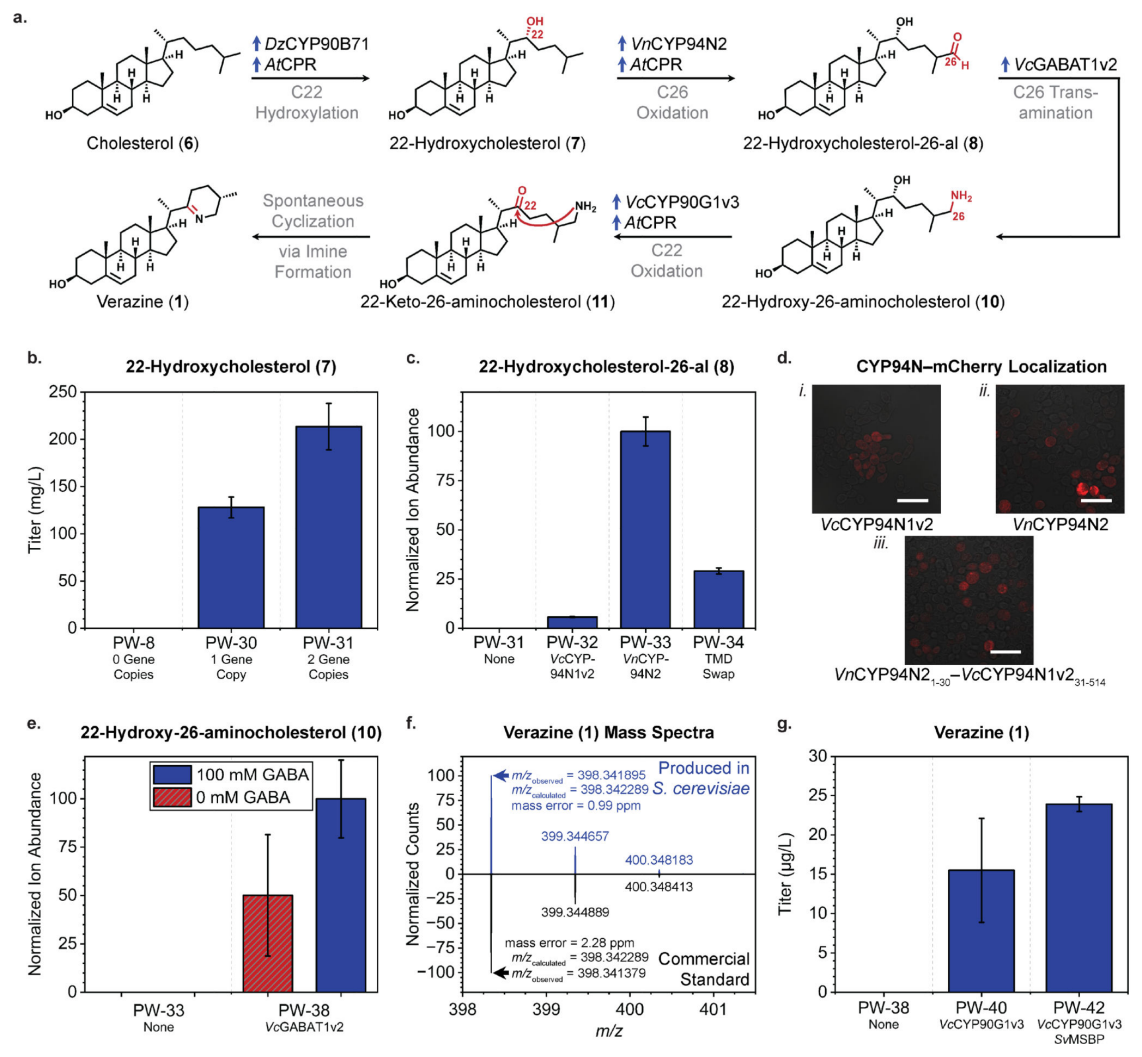
**a**. The cholesterol (**6**) to verazine (**1**) biosynthetic pathway was refactored step-by-step in *S. cerevisiae*. **b**. Titers for 22-hydroxycholesterol (**7**) as measured by GC-MS across a screen of 0, 1, and 2 gene copies of *Dz*CYP90B71/*At*CPR. **c**. Normalized ion abundances for 22-hydroxycholesterol-26-al (**8**) after derivatization to (**9**) as measured by LC-MS across a screen of different 26-oxidases. **d**. Confocal microscopy images characterizing the localization of *i. Vc*CYP94N1v2–mCherry *ii. Vn*CYP94N2–mCherry, and *iii. Vn*CYP94N2_1-30_–*Vc*CYP94N1v2_31-514_–mCherry. Larger versions of these images can be found in Supplementary Figure S21d-f. Scale bars are 20 µm. **e**. Normalized ion abundances for 22-hydroxy-26-aminocholesterol (**10**) as measured by LC-MS without (dashed red) and with GABA (blue). **f**. High resolution LC-MS characterization for verazine (**1**) produced in *S. cerevisiae* (top, blue) and from a commercial standard (bottom, black). **g**. Titers for verazine (**1**) as measured by LC-MS.

An inducible, engineered biosynthetic pathway to convert cholesterol (**6**) to verazine (**1**) (*i.e*., C22 hydroxylation of cholesterol [**6**], C26 oxidation of 22-hydroxycholesterol [**7**], C26 transamination of 22-hydroxycholesterol-26-al [**8**], C22 oxidation of 22-hydroxy-26-aminocholesterol [**10**], and spontaneous cyclization of 22-keto-26-aminocholesterol [**11**])^61,62^ was refactored step-by-step into the cholesterol (**6**)-producing *S. cerevisiae* strain PW-8 (Figure 3a). The details for each *S. cerevisiae* strain constructed towards the biosynthesis of verazine (**1**) are listed in Supplementary Table S7.

### 3.3 22-Hydroxycholesterol biosynthesis in *S. cerevisiae*

The first step to convert cholesterol (**6**) into verazine (**1**) is the cytochrome P450 (CYP450)-mediated C22 hydroxylation of cholesterol (**6**) (Figure 3a). The CYP450/cytochrome P450 reductase (CPR) pair used to refactor this step in *Spodoptera frugiperda* Sf9 insect cell culture^61^ and *Camelina sativa* seed^64^ (*i.e*., *Vc*CYP90B27v1 from *V. californicum* and *Ec*CPR from *Eschscholzia californica*) was selected as the starting point to construct a 22-hydroxycholesterol (**7**)-producing *S. cerevisiae* strain. Galactose-inducible copies of the genes encoding the enzymes *Vc*CYP90B27v1 and *Ec*CPR were integrated into strain PW-8 at ARS1014 using the CRISPR/Cas9 toolkit. When the resulting strain, PW-9, was incubated with shaking for 48 h in the growth stage (1× YP + glucose [2% w/v]) and 48 h in the production stage (1× YP + galactose [2% w/v] + copper (II) sulfate [200 µM]), it produced detectable amounts of 22-hydroxycholesterol (**7**) (Supplementary Figure S8).

To identify a CYP450/CPR pair that produces higher titers of 22-hydroxycholesterol (**7**), homologs of both *Vc*CYP90B27v1 and *Ec*CPR were screened. First, 12 CYP450s reported to perform C22 hydroxylation on sterols in plants (*i.e*., *Pp*CYP90B27, *Ar*CYP71D443, *At*CYP90B1, *Os*CYP90B2, *Sl*CYP90B3, *Tf*CYP90B51, *Pp*CYP90B52, *Dz*CYP90B71, *Ia*CYP708A15v2, *At*CYP724A1, *Os*CYP724B1, and *Sl*CYP724B2)^92^ were integrated along with *Ec*CPR into strain PW-8 at ARS1014 using the CRISPR/Cas9 toolkit, generating strains PW-11–PW-22. When the production of 22-hydroxycholesterol (**7**) in these strains was compared to strain PW-9, strain PW-18 containing *Dz*CYP90B71/*Ec*CPR produced the most 22-hydroxycholesterol (**7**) (Supplementary Figure S17a). Next, 5 different CPRs (*i.e*., *Ec*CPR, *Aa*CPR, *At*CPR, *Ps*CPR, and *Dz*CPR) were screened in combination with *Dz*CYP90B71 through plasmid-based expression in strains PW-24–PW-28. While each strain produced 22-hydroxycholesterol (**7**), strain PW-26 with *Dz*CYP90B71/*At*CPR showed the highest production titer (Supplementary Figure S17b). Importantly, confocal microscopy suggested that *Dz*CYP90B71–mCherry (strain PW-23) and *At*CPR–mCherry (strain PW-29) both localized to the endoplasmic reticulum (ER) membrane where we hypothesize that the cholesterol (**6**) to verazine (**1**) biosynthetic pathway occurs (Supplementary Figure S21a-c). With *Dz*CYP90B71/*At*CPR selected as the CYP450/CPR pair to move forward with in this work, galactose-inducible copies of the genes encoding this pair of enzymes were integrated into strain PW-8 at ARS1014 using the CRISPR/Cas9 toolkit creating strain PW-30, which produced 22-hydroxycholesterol (**7**) at titers of 128 ± 11 mg/L (8.3 ± 0.7 mg/g DCW; Figure 3b and Supplementary Figure S15d). Integrating a second gene copy of *Dz*CYP90B71 and *At*CPR into PW-30 at ARS416 using the same strategy generated strain PW-31, which produced the highest titers of 22-hydroxycholesterol (**7**) at 213 ± 25 mg/L (15 ± 2 mg/g DCW; Figure 3b). Importantly, the *S. cerevisiae*-produced 22-hydroxycholesterol (**7**) matched retention times and mass spectra via GC-MS compared to a commercial standard for 22-hydroxycholesterol (**7**) (Supplementary Figure S9).

### 3.4 22-Hydroxycholesterol-26-al biosynthesis in *S. cerevisiae*

The second step in this pathway is the CYP450-mediated C26 oxidation of 22-hydroxycholesterol (**7**) (Figure 3a). Galactose-inducible copies of the genes encoding the enzymes *Vc*CYP94N1v2 from *V. californicum* and an additional gene copy of *At*CPR were integrated into strain PW-31 at ARS911 using the CRISPR/Cas9 toolkit, constructing strain PW-32. This strain was incubated with shaking for 48 h in the growth stage (1× YP + glucose [2% w/v]) and 48 h in the production stage (1× YP + galactose [2% w/v] + copper (II) sulfate [200 µM]). However, no production of 22-hydroxycholesterol-26-al (**8**) was observed by GC-MS or liquid chromatography-mass spectrometry (LC-MS), likely due to lacking functional groups easily ionized by ESI. Therefore, aldehydes in the yeast extract were derivatized through a reaction with *o*-(carboxymethyl)hydroxylamine hemihydrochloride to increase their signal intensity on LC-MS^70^ (Supplementary Figure S11a). While the resulting derivatized 22-hydroxycholesterol-26-al (**9**) had no available commercial standard, this molecule was observed by LC-MS with samples sourced from both *S. cerevisiae* and *N. benthamiana* (Figure 3c; Supplementary Figure S10).

To identify a homolog of *Vc*CYP94N1v2 that produces higher titers of 22-hydroxycholesterol-26-al (**8**), we mined the transcriptome of *V. nigrum* and found an enzyme that proved to be identical to *Vn*CYP94N2 described in a recent report (Supplementary Figure S3).^62^ VnCYP94N2 is highly homologous to both CYP94N P450s from *V. californicum* described previously;^61^ however, its N-terminal transmembrane domain (TMD) is most homologous to that of *Vc*CYP94N2v2. We hypothesized that this alternate TMD may provide a better foothold for localization of these proteins in the ER membrane when expressed in *S. cerevisiae*. Galactose-inducible copies of the genes encoding the enzymes *Vn*CYP94N2 and an additional gene copy of *At*CPR were integrated into strain PW-31 at ARS911 using the CRISPR/Cas9 toolkit, constructing strain PW-33. Switching from *Vc*CYP94N1v2 in strain PW-32 to *Vn*CYP94N2 in strain PW-33 resulted in an increase by 17× in 22-hydroxycholesterol-26-al (**8**) titers (Figure 3c). Importantly, the *S. cerevisiae*-produced and derivatized 22-hydroxycholesterol-26-al (**9**) matched retention times and mass spectra via LC-MS compared to *N. benthamiana*-produced and derivatized 22-hydroxycholesterol-26-al (**9**) (Supplementary Figure S11).

We hypothesized that the difference in product titers between *Vc*CYP94N1v2 and *Vn*CYP94N2 can partially be explained by the N-terminal TMD-driven localization of these two enzymes. Confocal microscopy of *Vc*CYP94N1v2–mCherry (strain PW-35) suggested that this protein localized to the cytosol, while suggesting that *Vn*CYP94N2–mCherry (strain PW-36) was localized to the ER membrane (Figure 3d i-ii). When the N-terminal TMDs (predicted by DTU’s TMHMM-2.0 tool to be located within the first 30 amino acids of each enzyme)^93^ on these enzymes were swapped (*i.e*., *Vn*CYP94N2_1-30_–*Vc*CYP94N1v2_31-514_), utilization of the resulting enzyme (strain PW-34) led to an increase by 5× in 22-hydroxycholesterol-26-al (**8**) production compared to *Vc*CYP94N1 in strain PW-32 (Figure 3c). Additionally, confocal microscopy of *Vn*CYP94N2_1-30_–*Vc*CYP94N1v2_31-514_–mCherry (strain PW-37) suggested that this engineered enzyme localized to the ER membrane (Figure 3d iii). Together, the N-terminal tag of *Vn*CYP94N2_1-30_ helps these CYP450s localize to the ER membrane, and adding it to a CYP450 increased the titers of its product in *S. cerevisiae*.

### 3.5 22-Hydroxy-26-aminocholesterol biosynthesis in *S. cerevisiae*

The third step in this pathway is the γ-aminobutyric acid (GABA) aminotransferase C26 transamination of 22-hydroxycholesterol-26-al (**8**) (Figure 3a). A galactose-inducible copy of the gene encoding the enzyme *Vc*GABAT1v2 from *V. californicum* was integrated into strain PW-33 at ARS720 using the CRISPR/Cas9 toolkit, constructing strain PW-38. This strain produced 22-hydroxy-26-aminocholesterol (**10**), and this molecule matched retention times and mass spectra via LC-MS compared to *N. benthamiana*-produced 22-hydroxy-26-aminocholesterol (**10**) (Supplementary Figure S12). Feeding GABA increased production of 22-hydroxy-26-aminocholesterol (**10**) by 2× in strain PW-38 (Figure 3e), indicating that GABA levels are a limiting factor in the biosynthesis of this molecule. Plasmid-based expression of the fusion *Vc*GABAT1v2–mCherry (strain PW-39) suggested localization to the ER membrane (Supplementary Figure S21g) despite there being no predicted transmembrane domain in this enzyme.

### 3.6 Verazine biosynthesis in *S. cerevisiae*

The fourth and fifth steps in this pathway are the CYP450-mediated C22 oxidation of 22-hydroxy-26-aminocholesterol (**10**) and the spontaneous cyclization through imine formation of 22-keto-26-aminocholesterol (**11**) (Figure 3a). Galactose-inducible copies of the genes encoding the enzymes *Vc*GABAT1v2 and *Vc*CYP90G1v3 from *V. californicum* were integrated into strain PW-33 at ARS720 using the CRISPR/Cas9 toolkit, constructing strain PW-40. While no 22-keto-26-aminocholesterol (**11**) was detected, this *S. cerevisiae* strain produced verazine (**1**) that was indistinguishable from *Nicotiana benthamiana* (*N. benthamiana*)-produced verazine (**1**), and a commercial standard (*Veratrum* spp. plant-produced) of verazine (**1**) as confirmed by LC-MS (Figure 3f; Supplementary Figures S13 and S14). To the best of our knowledge, this is the first report describing the heterologous biosynthesis of a steroidal alkaloid in an engineered yeast. Plasmid-based expression of the fusion *Vc*CYP90G1v3–mCherry (strain PW-41) suggested localization to the ER membrane (Supplementary Figure S21h). Strain PW-40 produced verazine (**1**) at a titer of 16 ± 7 µg/L (0.92 ± 0.38 µg/g DCW; Figure 3g; Supplementary Figure S18).

### 3.7 DBTL scale-up of verazine production in *S. cerevisiae*

**Figure 4:**
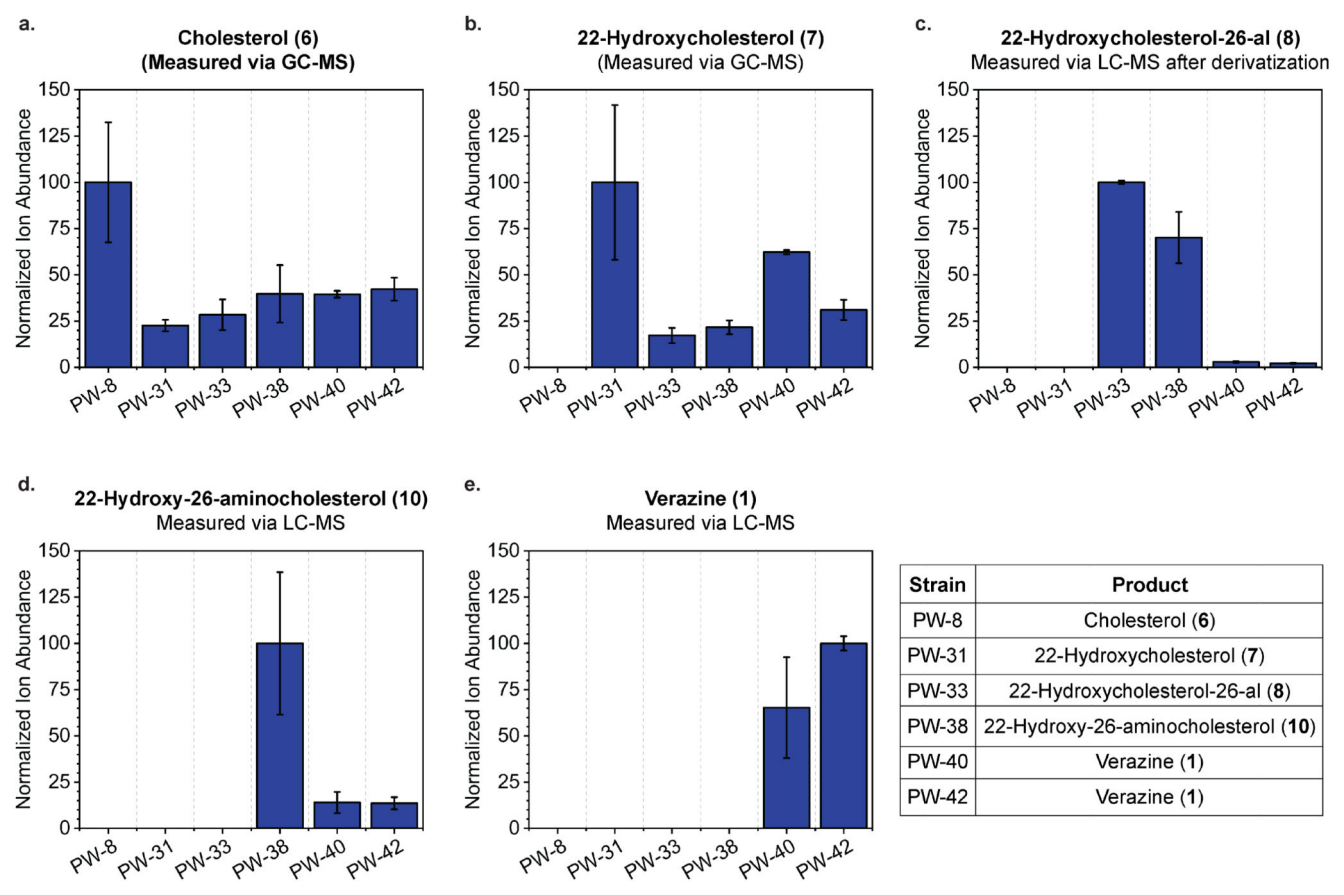
**a.-e**. For strains containing partially and fully refactored cholesterol (**6**) to verazine (**1**) biosynthetic pathways, the relative production of each individual molecule between different strains was quantified by GC-MS for cholesterol (**6**) and 22-hydroxycholesterol (**7**) as well as LC-MS for 22-hydroxycholesterol-26-al (**8**) after derivatization to (**9**), 22-hydroxy-26-aminocholesterol (**10)**, and verazine (**1**). Ion abundances were normalized independently for each molecule to the highest ion abundance for that molecule in this dataset.

In the refactored cholesterol (**6**) to verazine (**1**) biosynthetic pathway, each heterologous enzyme was successfully expressed based on proteomics characterization (Supplementary Figure S22) and localized to the ER membrane based on confocal microscopy (Supplementary Figure S21). To look for bottlenecks in verazine (**1**) biosynthesis, we looked at the production of verazine (**1**) and each of its precursors across strains with partially and fully refactored pathways (Figure 4). When ion abundances were independently normalized for each molecule, we observed that the amount of each molecule decreased when the enzyme that uses it as a substrate was introduced. Interestingly, 22-hydroxycholesterol-26-al (**8**) was the only verazine (**1**) precursor that was quantitatively utilized once later steps in the pathway were introduced, which suggests that the rate-limiting step in verazine (**1**) biosynthesis is the C26 oxidation by *Vn*CYP94N2 (Figure 4).

To try to increase verazine (**1**) titers, a gene copy of *Sv*MSBP, the membrane steroid binding protein (MSBP) from *Saponaria vaccaria*, under control of a galactose-inducible promoter into strain PW-40 at ARS511 via the CRISPR/Cas9 toolkit, creating strain PW-42. This increased verazine (**1**) titers to 24 ± 1 µg/L (1.4 ± 0.04 µg/g DCW), 1.5× (Figure 3g) more than strain PW-40. While multiple inductions did not result in increased verazine (**1**) titers in strain PW-42, extending the production time for this strain from 48 h to 120 h led to slight increase in verazine (**1**) titer to 27 ± 2 µg/L (1.7 ± 0.1 µg/g DCW) The slight increase in verazine production with the longer production phase suggests that verazine is stable in the *S. cerevisiae* cellular environment. Despite the reported anti-fungal properties of verazine (**1**), no growth defect was observed as strains were engineered from PW-4 to PW-42. Interestingly, while there was no detectable utilization of GABA (Supplementary Figure S20), increasing GABA concentration led to a slight increase in verazine (**1**) production (Supplementary Figure 19a). Surprisingly, the removal of copper (II) sulfate from the production stage medium led to a 2× increase in verazine (**1**) titers to 83 ± 3 µg/L (4.1 ± 0.1 µg/g DCW) (Supplementary Figure S19b), an amount that compares favorably to the 5.11 µg/g dry weight production in *N. benthamiana*.^62^ Additional improvements on verazine (**1**) titers are expected with additional metabolic engineering of strain PW-42.

## 4 Conclusion

In conclusion, *S. cerevisiae* was engineered to refactor the biosynthesis of verazine, a biosynthetic precursor to cyclopamine, from *V. californicum* and *V. nigrum* plants. The inducible upregulation of the mevalonate and sterol biosynthetic pathways, diversion of biosynthetic flux from ergosterol to cholesterol, and expression of eight heterologous proteins sourced from seven different species were combined to produce verazine in *S. cerevisiae* at a final titer of 83 ± 3 µg/L (4.1 ± 0.1 µg/g DCW) in strain PW-42. Importantly, each *S. cerevisiae*-produced verazine and precursor was indistinguishable via GC-MS or LC-MS from commercial standards and/or standards from heterologous production in *N. benthamiana*. To the best of our knowledge, this is the first report of heterologous production of a steroidal alkaloid in an engineered yeast. This microbial production platform for verazine will not only reduce the time, cost, and environmental impact of verazine production, but will also lays the groundwork for future microbial biosynthesis of other steroidal alkaloid natural products like cyclopamine, potentially enabling the rapid diversification of steroidal alkaloids to generate libraries of drug candidates.

## Supplementary Material

Supplementary Information

## Data Availability

The generated mass spectrometry proteomics data have been deposited to the ProteomeXchange Consortium via the PRIDE partner repository with the dataset identifier PXD053024.^94^ Other data will be made available upon reasonable request.

## Highlights

- Biosynthesis of verazine (83 µg/L) from glucose and galactose in *S. cerevisiae*
- First heterologous steroidal alkaloid produced in an engineered yeast
- Biosynthesis of verazine via transient expression of its pathway in *N. benthamiana*
- *S. cerevisiae, N. benthamiana*, and commercial verazine were indistinguishable
