# Supplementary material for: Verazine Biosynthesis from Simple Sugars in Engineered Saccharomyces cerevisiae
Source: Metab Eng. Author manuscript; Available in PMC 2026 Feb 2. (PMC11421371; doi:10.1016/j.ymben.2024.07.011)
Supplement: Supplementary Information [file EMS212229-supplement-Supplementary_Information.pdf]

Verazine Biosynthesis from Simple Sugars in Engineered *Saccharomyces cerevisiae*

TABLE OF CONTENTS

1. CHEMICALS 2

1.1. Guide of chemicals 2

1.2. Guide of *S. cerevisiae*- and *N. benthamiana*-produced metabolites 4

2. GENES, PLASMIDS, *S. CEREVISIAE* STRAINS, AND *N. BENTHAMIANA* TRANSIENT EXPRESSION SYSTEMS 6

2.1. Guide of genes 6

2.2. Guide of plasmids 59

2.3. Guide of *S. cerevisiae* strains 62

2.4. Guide of *N. benthamiana* transient expression systems 67

3. SUPPLEMENTARY DATA 68

3.1. Native verazine biosynthetic pathways in *V. californicum and V. nigrum* 68

3.2. Native mevalonate, triterpene, and sterol biosynthetic pathways in *S. cerevisiae* ... 69

3.3. Gene mining for *V. nigrum* enzymes 70

3.4. GC-MS characterization 71

3.5. LC-MS characterization 77

3.6. DBTL optimization of titers 82

3.7. Confocal microscopy 88

3.8. Proteomics 89

4. REFERENCES 90

1. CHEMICALS

1.1. Guide of chemicals

Supplementary Table S1. Chemicals used in this study for feeding, analyte derivatization, or analytical standards. This table continues to page 3.

Chemical Name	CAS	Supplier	Catalog Number
Squalene	111-02-4	Thomas Scientific LLC	C955F39
Ergosterol	57-87-4	Millipore Sigma	PHR1512
Cholesterol	57-88-5	Millipore Sigma	C8667
22(R)-Hydroxycholesterol	17954-98-2	Millipore Sigma	H9384
22-Hydroxycholesterol-26-al	n/a	John Innes Center (Osbourn Lab)	n/a
22-Hydroxy-26-aminocholesterol	n/a	John Innes Center (Osbourn Lab)	n/a
Verazine	14320-81-1	MedChemExpress	HY-N11911
Verazine	14320-81-1	John Innes Center (Osbourn Lab)	n/a
o-(Carboxymethyl)hydroxylamine hemihydrochloride	2921-14-4	Millipore Sigma	C13408
Diethyl ethoxymethylenemalonate	87-13-8	Millipore Sigma	D94208
Betaine	107-43-7	Millipore Sigma	B2629
SOC medium	n/a	New England Biolabs	B9020S
LB medium	n/a	Research Products International	L24045
Carbenicillin solution	n/a	Teknova	C2136
Gibco yeast extract	n/a	Thermo Fisher Scientific	211929
Gibco polypeptone peptone	n/a	Thermo Fisher Scientific	211910

Chemical Name	CAS	Supplier	Catalog Number
D-(+)-Glucose	50-99-7	Millipore Sigma	G8270
D-(+)-Galactose	59-23-4	Millipore Sigma	G0625
G418 sulfate	108321-42-2	Thermo Fisher Scientific	A1720
Copper (II) sulfate	7758-98-7	Millipore Sigma	C1297
γ-Aminobutyric acid	56-12-2	Millipore Sigma	A2129
Complete minimal medium minus uracil	n/a	Sunrise Science	1306-030
Ammonium sulfate	7783-20-2	Millipore Sigma	A4418
BD Difco yeast nitrogen base without amino acids and ammonium sulfate	n/a	Fisher Scientific	DF0335-15-9
Potassium phosphate dibasic	7758-11-4	Millipore Sigma	P3786
Potassium phosphate monobasic	7778-77-0	Millipore Sigma	P5655
Potassium hydroxide	1310-58-3	Millipore Sigma	221473
100% Ethanol	64-17-5	Decon Labs, Inc.	V1001
GC-MS grade *n*-hexane	110-54-3	Millipore Sigma	100795
LC-MS grade methanol	67-56-1	VWR	JT9830-3
LC-MS grade water	7732-15-5	VWR	BJAH365-4
LC-MS grade acetonitrile	75-05-8	Fisher Scientific	A955
Formic acid	64-18-6	Thermo Fisher Scientific	94318

1.2. Guide of *S. cerevisiae*- and *N. benthamiana*-produced metabolites

Supplementary Table S2. Chemicals produced in this study. This table continues to page 5.

Number	Chemical Name	Structure
**1**	Verazine	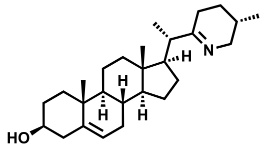
**2**	Squalene	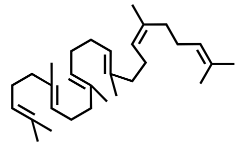
**3**	Lanosterol	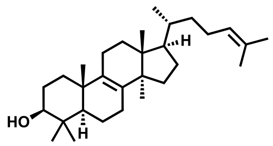
**4**	7-Dehydrodesmosterol	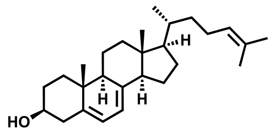
**5**	Ergosterol	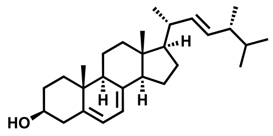
**6**	Cholesterol	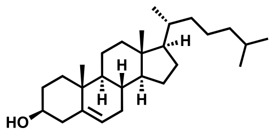
**7**	22-Hydroxycholesterol	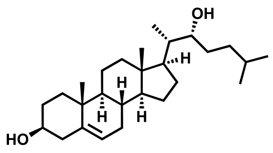

Number	Chemical Name	Structure
**8**	22-Hydroxycholesterol-26-al	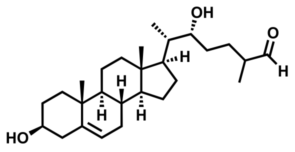
**9**	Derivatized 22-hydroxycholesterol-26-al	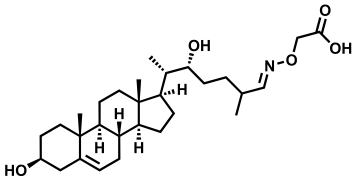
**10**	22-Hydroxy-26-aminocholesterol	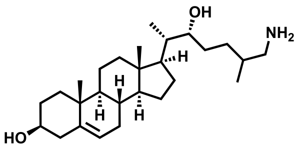
**11**	22-Keto-26-aminocholesterol	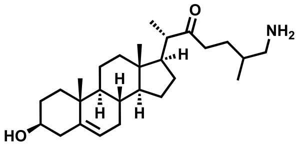

2. GENES, PLASMIDS, *S. CEREVISIAE* STRAINS, AND *N. BENTHAMIANA* TRANSIENT EXPRESSION SYSTEMS

2.1. Guide of genes

Supplementary Table S3. Genes used for homologous and heterologous expression in this study. This table continues to page 8.

Protein Name	Origin Species	Abbreviated protein name	GenBank Accession Code
Squalene epoxidase	*Saccharomyces cerevisiae*	ERG1	NP_011691.1
Farnesyl-diphosphate farnesyl transferase (squalene synthase)	*Saccharomyces cerevisiae*	ERG9	AAA34597.1
Farnesyl pyrophosphate synthetase	*Saccharomyces cerevisiae*	ERG20	AJP39545.1
Lanosterol synthase	*Saccharomyces cerevisiae*	ERG7	NP_011939.2
Δ7(8)-sterol reductase	*Danio rerio*	*Dr*DHCR7	NP_958487.2
Δ24(25)-sterol reductase	*Danio rerio*	*Dr*DHCR24	NP_001008645.1
Δ7(8)-sterol reductase	*Solanum tuberosum*	*St*DHCR7	XP_006362628.1
Δ24(25)-sterol reductase	*Gallus gallus*	*Gg*DHCR24	NP_001026459.1
Cholesterol 22-hydroxylase	*Veratrum californicum*	*Vc*CYP90B27v1	AJT59558.1
Cytochrome P450 reductase	*Eschscholzia californica*	*Ec*CPR	AAC05022.1
Cholesterol 22-hydroxylase–mCherry fusion	n/a	*Vc*CYP90B27v1–mCherry	n/a
Cholesterol 22-hydroxylase	*Paris polyphylla*	*Pp*CYP90B27	A0A5A4DV62.1
Cholesterol 22-hydroxylase	*Ajuga reptans*	*Ar*CYP71D443	BAS30379.1
Cholesterol 22-hydroxylase	*Arabidopsis thaliana*	*At*CYP90B1	NP_190635.1
Cholesterol 22-hydroxylase	*Oryza sativa*	*Os*CYP90B2	NP_001404356.1
Cholesterol 22-hydroxylase	*Solanum lycopersicum*	*Sl*CYP90B3	NP_001266259.2
Cholesterol 22-hydroxylase	*Trigonella foenum-graecum*	*Tf*CYP90B51	A0A517FNC6.1
Cholesterol 22-hydroxylase	*Paris polyphylla*	*Pp*CYP90B52	A0A517FNB9.1
Cholesterol 22-hydroxylase	*Dioscorea zingiberensis*	*Dz*CYP90B71	QPZ88854.1
Cholesterol 22-hydroxylase	*Iberis amara*	*Ia*CYP708A15v2	UAK14950.1

**Protein Name**	**Origin Species**	**Abbreviated protein name**	**GenBank Accession Code**
Cholesterol 22-hydroxylase	*Arabidopsis thaliana*	*At*CYP724A1	NP_001331288.1
Cholesterol 22-hydroxylase	*Oryza sativa*	*Os*CYP724B1	NP_001389305.1
Cholesterol 22-hydroxylase	*Solanum lycopersicum*	*Sl*CYP724B2	NP_001266072.1
Cholesterol 22-hydroxylase–mCherry fusion	n/a	*Dz*CYP90B71–mCherry	n/a
Cytochrome P450 reductase	*Artemisia annua*	*Aa*CPR	ABC47946.1
Cytochrome P450 reductase	*Arabidopsis thaliana*	*At*CPR	NP_194183.1
Cytochrome P450 reductase	*Papaver somniferum*	*Ps*CPR	B1NF18.1
Cytochrome P450 reductase	*Dioscorea zingiberensis*	*Dz*CPR	KAJ0985076.1
Cytochrome P450 reductase–mCherry fusion	n/a	*At*CPR–mCherry	n/a
22-Hydroxycholesterol 26-oxidase	*Veratrum californicum*	*Vc*CYP94N1v2	AJT59561.1
22-Hydroxycholesterol 26-oxidase	*Veratrum nigrum*	*Vn*CYP94N2	WRU85179.1
22-Hydroxycholesterol 26-oxidase	n/a	*Vn*CYP94N2$_{1\text{-}30}$–*Vc*CYP94N1v2$_{31\text{-}514}$	n/a
22-Hydroxycholesterol 26-oxidase–mCherry fusion	n/a	*Vc*CYP94N1v2–mCherry	n/a
22-Hydroxycholesterol 26-oxidase–mCherry fusion	n/a	*Vn*CYP94N2–mCherry	n/a
22-Hydroxycholesterol 26-oxidase–mCherry fusion	n/a	*Vn*CYP94N2$_{1\text{-}30}$–*Vc*CYP94N1v2$_{31\text{-}514}$–mCherry	n/a
22-Hydroxycholesterol-26-al 26-transaminase	*Veratrum californicum*	*Vc*GABAT1v2	AJT59569.1
22-Hydroxycholesterol-26-al 26-transaminase–mCherry fusion	n/a	*Vc*GABAT1v2–mCherry	n/a
22-Hydroxy-26-aminocholesterol 22-oxidase	*Veratrum californicum*	*Vc*CYP90G1v3	AJT59566.1
22-Hydroxy-26-aminocholesterol 22-oxidase–mCherry fusion	n/a	*Vc*CYP90G1v3–mCherry	n/a

Protein Name	Origin Species	Abbreviated protein name	GenBank Accession Code
Membrane steroid binding protein	*Saponaria vaccaria*	*Sv*MSBP	n/a[1]

Abbreviated protein name:

ERG1

Amino acid sequence:

MSAVNVAPELINADNTITYDAIVIGAGVIGPCVATGLARKGKKVLIVERDWAMPDRIVGELMQPG
GVRALRSLGMIQSINNIEAYPVTGYTVFFNGEQVDIPYPYKADIPKVEKLKDLVKDGNDKVLEDS
TIHIKDYEDDERERGVAFVHGRFLNNLRNITAQEPNVTRVQGNCIEILKDEKNEVVGAKVDIDGR
GKVEFKAHLTFICDGIFSRFRKELHPDHVPTVGSSFVGMSLFNAKNPAPMHGHVILGSDHMPILV
YQISPEETRILCAYNSPKVPADIKSWMIKDVQPFIPKSLRPSFDEAVSQGKFRAMPNSYLPARQN
DVTGMCVIGDALNMRHPLTGGGMTVGLHDVVLLIKKIGDLDFSDREKVLDELLDYHFERKSYDS
VINVLSVALYSLFAADSDNLKALQKGCFKYFQRGGDCVNKPVEFLSGVLPKPLQLTRVFFAVAFY
TIYLNMEERGFLGLPMALLEGIMILITAIRVFTPFLFGELIG-

DNA sequence from CEN.PK2-1C yeast for studies in *S. cerevisiae*:

atgtctgctgttaacgttgcacctgaattgattaatgccgacaacacaattacctacgatgcgattgtcatcggtgctggtgttatcggtcca
tgtgttgctactggtctagcaagaaagggtaagaaagttcttatcgtagaacgtgactgggctatgcctgatagaattgttggtgaattgat
gcaaccaggtggtgttagagcattgagaagtctgggtatgattcaatctatcaacaacatcgaagcatatcctgttaccggttataccgt
ctttttcaacggcgaacaagttgatattccataccсttacaaggccgatatccctaaagttgaaaaattgaaggacttggtcaaagatgg
taatgacaaggtcttggaagacagcactattcacatcaaggattacgaagatgatgaaagagaaaggggtgttgcttttgttcatggta
gattcttgaacaacttgagaaacattactgctcaagagccaaatgttactagagtgcaaggtaactgtattgagatattgaaggatgaa
aagaatgaggttgttggtgccaaggttgacattgatggccgtggcaaggtggaattcaaagcccacttgacatttatctgtgacggtatct
tttcacgtttcagaaaggaattgcacccagaccatgttccaactgtcggttcttcgtttgtcggtatgtctttgttcaatgctaagaatcctgct
cctatgcacggtcacgttattcttggtagtgatcatatgccaatcttggtttaccaaatcagtccagaagaaacaagaatcctttgtgcttac
aactctccaaaggtcccagctgatatcaagagttggatgattaaggatgtccaacctttcattccaaagagtctacgtccttcatttgatga
agccgtcagccaaggtaaatttagagctatgccaaactcctacttgccagctagacaaaacgacgtcactggtatgtgtgttatcggtg
acgctctaaatatgagacatccattgactggtggtggtatgactgtcggtttgcatgatgttgtcttgttgattaagaaaataggtgacctag
acttcagcgaccgtgaaaaggttttggatgaattactagactaccatttcgaaagaaagagttacgattccgttattaacgttttgtcagtg
gctttgtattctttgttcgctgctgacagcgataacttgaaggcattacaaaaaggttgtttcaaatatttccaaagaggtggcgattgtgtca
acaaacccgttgaatttctgtctggtgtcttgccaaagcctttgcaattgaccagggttttcttcgctgtcgctttttacaccatttacttgaaca
tggaagaacgtggtttcttgggattaccaatggctttattggaaggtattatgattttgatcacagctattagagtattcaccccatttttgtttg
gtgagttgattggttaa

Abbreviated protein name:

ERG9

Amino acid sequence:

MGKLLQLALHPVEMKAALKLKFCRTPLFSIYDQSTSPYLLHCFELLNLTSRSFAAVIRELHPELRN
CVTLFYLILRALDTIEDDMSIEHDLKIDLLRHFHEKLLLTKWSFDGNAPDVKDRAVLTDFESILIEFH
KLKPEYQEVIKEITEKMGNGMADYILDENYNLNGLQTVHDYDVYCHYVAGLVGDGLTRLIVIAKF
ANESLYSNEQLYESMGLFLQKTNIIRDYNEDLVDGRSFWPKEIWSQYAPQLKDFMKPENEQLGL
DCINHLVLNALSHVIDVLTYLASIHEQSTFQFCAIPQVMAIATLALVFNNREVLHGNVKIRKGTTCY
LILKSRTLRGCVEIFDYYLRDIKSKLAVQDPNFLKLNIQISKIEQFMEEMYQDKLPPNVKPNETPIF
LKVKERSRYDDELVPTQQEEEYKFNMVLSIILSVLLGFYYIYTLHRA-

DNA sequence from CEN.PK2-1C yeast for studies in *S. cerevisiae*:

atgggaaagctattacaattggcattgcatccggtcgagatgaaggcagctttgaagctgaagttttgcagaacaccgctattctccatc
tatgatcagtccacgtctccatatctcttgcactgtttcgaactgttgaacttgacctccagatcgtttgctgctgtgatcagagagctgcatc
cagaattgagaaactgtgttactctcttttatttgattttaagggctttggataccatcgaagacgatatgtccatcgaacacgatttgaaaat
tgacttgttgcgtcacttccacgagaaattgttgttaactaaatggagtttcgacggaaatgcccccgatgtgaaggacagagccgttttg
acagatttcgaatcgattcttattgaattccacaaattgaaaccagaatatcaagaagtcatcaaggagatcaccgagaaaatgggta
atggtatggccgactacatcttggatgaaaattacaacttgaatgggttgcaaaccgtccacgactacgacgtgtactgtcactacgtag
ctggtttggtcggtgatggtttgacccgtttgattgtcattgccaagtttgccaacgaatctttgtattctaatgagcaattgtatgaaagcatg
ggtcttttcctacaaaaaaccaacatcatcagagactacaatgaagatttggtcgatggtagatccttctggcccaaggaaatctggtca
caatacgctcctcagttgaaggacttcatgaaacctgaaaacgaacaactggggttggactgtataaaccacctcgtcttaaacgcatt
gagtcatgttatcgatgtgttgacttatttggccagtatccacgagcaatccactttccaattttgtgccattccccaagttatggccattgca
accttggctttggtattcaacaaccgtgaagtgctacatggcaatgtaaagattcgtaagggtactacctgctatttaattttgaaatcaag
gactttgcgtggctgtgtcgagatttttgactattacttacgtgatatcaaatctaaattggctgtgcaagatccaaatttcttaaaattgaaca
ttcaaatctccaagatcgaacaattcatggaagaaatgtaccaggataaattacctcctaacgtgaagccaaatgaaactccaattttct
tgaaagttaaagaaagatccagatacgatgatgaattggtcccaacccaacaagaagaagagtacaagttcaatatggttttatctat
catcttgtccgttcttcttgggttttattatatatacactttacacagagcgtga

Abbreviated protein name:

ERG20

Amino acid sequence:

MASEKEIRRERFLNVFPKLVEELNASLLAYGMPKEACDWYAHSLNYNTPGGKLNRGLSVVDTY
AILSNKTVEQLGQEEYEKVAILGWCIELLQAYFLVADDMMDKSITRRGQPCWYKVPEVGEIAIND
AFMLEAAIYKLLKSHFRNEKYYIDITELFHEVTFQTELGQLMDLITAPEDKVDLSKFSLKKHSFIVT
FKTAYYSFYLPVALAMYVAGITDEKDLKQARDVLIPLGEYFQIQDDYLDCFGTPEQIGKIGTDIQD
NKCSWVINKALELASAEQRKTLDENYGKKDSVAEAKCKKIFNDLKIDQLYHEYEESVAKDLKAKI
SQVDESRGFKADVLTAFLNKVYKRSK-

DNA sequence from CEN.PK2-1C yeast for studies in *S. cerevisiae*:

atggcttcagaaaaagaaattaggagagagagattcttgaacgttttccctaaattagtagaggaattgaacgcatcgcttttggcttac
ggtatgcctaaggaagcatgtgactggtatgcccactcattgaactacaacactccaggcggtaagttaaatagaggtttgtccgttgtg
gacacgtatgctattctctccaacaagaccgttgaacaattggggcaagaagaatacgaaaaggttgctattctaggttggtgcattga
gttgttgcaggcttacttcttggtcgccgatgatatgatggacaagtccattaccagaagaggccaaccatgttggtacaaggttcctga
agttggggaaattgccatcaatgacgcattcatgttagaggctgctatctacaagcttttgaaatctcacttcagaaacgaaaaatacta
catagatatcaccgaattgttccatgaagtcaccttccaaaccgaattgggccaattgatggacttaatcactgcacctgaagacaaag
tcgacttgagtaagttctccctaaagaagcactccttcatagttactttcaagactgcttactattctttctacttgcctgtcgcattggctatgt
acgttgccggtatcacagatgaaaaggatttgaaacaagccagagatgtcttgattccattgggtgaatatttccaaattcaagatgact
acttagactgcttcggtaccccagaacagatcggtaagatcggtacagatatccaagataacaaatgttcttgggtaatcaacaaggc
attagaacttgcttccgcagaacaaagaaagactttagacgaaaattacggtaagaaggactcagtcgcagaagccaaatgcaaa
aagattttcaatgacttgaaaatcgaccagttataccacgaatatgaagagtctgttgccaaggatttgaaggccaagatctcccaagt
cgacgagtctcgtggcttcaaagccgacgtcttaactgcgtttttgaacaaagtttacaagagaagcaaatag

Abbreviated protein name:

ERG7

Amino acid sequence:

MTEFYSDTIGLPKTDPRLWRLRTDELGRESWEYLTPQQAANDPPSTFTQWLLQDPKFPQPHPE
RNKHSPDFSAFDACHNGASFFKLLQEPDSGIFPCQYKGPMFMTIGYVAVNYIAGIEIPEHERIELI
RYIVNTAHPVDGGWGLHSVDKSTVFGTVLNYVILRLLGLPKDHPVCAKARSTLLRLGGAIGSPH
WGKIWLSALNLYKWEGVNPAPPETWLLPYSLPMHPGRWWVHTRGVYIPVSYLSLVKFSCPMT
PLLEELRNEIYTKPFDKINFSKNRNTVCGVDLYYPHSTTLNIANSLVVFYEKYLRNRFIYSLSKKK
VYDLIKTELQNTDSLCIAPVNQAFCALVTLIEEGVDSEAFQRLQYRFKDALFHGPQGMTIMGTNG
VQTWDCAFAIQYFFVAGLAERPEFYNTIVSAYKFLCHAQFDTECVPGSYRDKRKGAWGFSTKT
QGYTVADCTAEAIKAIIMVKNSPVFSEVHHMISSERLFEGIDVLLNLQNIGSFEYGSFATYEKIKAP
LAMETLNPAEVFGNIMVEYPYVECTDSSVLGLTYFHKYFDYRKEEIRTRIRIAIEFIKKSQLPDGS
WYGSWGICFTYAGMFALEALHTVGETYENSSTVRKGCDFLVSKQMKDGGWGESMKSSELHS
YVDSEKSLVVQTAWALIALLFAEYPNKEVIDRGIDLLKNRQEESGEWKFESVEGVFNHSCAIEYP
SYRFLFPIKALGMYSRAYETHTL-

DNA sequence from CEN.PK2-1C yeast for studies in *S. cerevisiae*:

atgacagaattttattctgacacaatcggtctaccaaagacagatccacgtctttggagactgagaactgatgagctaggccgagaaa
gctgggaatatttaacccctcagcaagccgcaaacgacccaccatccactttcacgcagtggcttcttcaagatcccaaatttcctcaa
cctcatccagaaagaaataagcattcaccagatttttcagccttcgatgcgtgtcataatggtgcatctttttcaaactgcttcaagagcct
gactcaggtatttttccgtgtcaatataaaggacccatgttcatgacaatcggttacgtagccgtaaactatatcgccggtattgaaattcct
gagcatgagagaatagaattaattagatacatcgtcaatacagcacatccggttgatggtggctggggtctacattctgttgacaaatcc
accgtgtttggtacagtattgaactatgtaatcttacgtttattgggtctacccaaggaccacccggtttgcgccaaggcaagaagcacat
tgttaaggttaggcggtgctattggatcccctcactggggaaaaatttggctaagtgcactaaacttgtataaatgggaaggtgtgaacc
ctgcccctcctgaaacttggttacttccatattcactgcccatgcatccggggagatggtgggttcatactagaggtgtttacattccggtca
gttacctgtcattggtcaaattttcttgcccaatgactcctcttcttgaagaactgaggaatgaaatttacactaaaccgtttgacaagatta
acttctccaagaacaggaataccgtatgtggagtagacctatattacccccattctactactttgaatattgcgaacagccttgtagtatttt
acgaaaaatacctaagaaaccggttcatttactctctatccaagaagaaggtttatgatctaatcaaaacggagttacagaatactgatt
ccttgtgtatagcacctgttaaccaggcgttttgcgcacttgtcactcttattgaagaaggggtagactcggaagcgttccagcgtctcca
atataggttcaaggatgcattgttccatggtccacagggtatgaccattatgggaacaaatggtgtgcaaacctgggattgtgcgtttgcc
attcaatactttttcgtcgcaggcctcgcagaaagacctgaattctataacacaattgtctctgcctataaattcttgtgtcatgctcaatttga
caccgagtgcgttccaggtagttatagggataagagaaagggggcttggggcttctcaacaaaaacacagggctatacagtggcag
attgcactgcagaagcaattaaagccatcatcatggtgaaaaactctcccgtctttagtgaagtacaccatatgattagcagtgaacgtt
tatttgaaggcattgatgtgttattgaacctacaaaacatcggatcttttgaatatggttcctttgcaacctatgaaaaaatcaaggcccca
ctagcaatggaaaccttgaatcctgctgaagtttttggtaacataatggtagaatacccatacgtggaatgtactgattcatccgttctggg
gttgacatattttcacaagtacttcgactataggaaagaggaaatacgtacacgcatcagaatcgccatcgaattcataaaaaaatctc
aattaccagatggaagttggtatggaagctggggtatttgttttacatatgccggtatgtttgcattggaggcattacacaccgtgggga
gacctatgagaattcctcaacggtaagaaaaggttgcgacttcttggtcagtaaacagatgaaggatggcggttgggggaatcaat
gaagtccagtgaattacatagttatgtggatagtgaaaaatcgctagtcgttcaaaccgcatgggcgctaattgcacttcttttcgctgaat
atcctaataaagaagtcatcgaccgcggtattgaccttttaaaaaatagacaagaagaatccggggaatggaaatttgaaagtgtag
aaggtgttttcaaccactcttgtgcaattgaatacccaagttatcgattcttattccctattaaggcattaggtatgtacagcagggcatatg
aaacacatacgctttaa

Abbreviated protein name:

*Dr*DHCR7

Amino acid sequence:

MMASDRVRKRHKGSANGAQTVEKEPSKEPAQWGRAWEVDWFSLSGVILLLCFAPFLVFFFIMA
CDQYQCSISHPLLDLYNGDATLFTIWNRAPSFTWAAAKIYAIWVTFQVVLYMCVPDFLHKILPGY
VGGVQDGARTPAGLINKYEVNGLQCWLITHVLWVLNAQHFHWFSPTIIIDNWIPLLWCTNILGYA
VSTFAFIKAYLFPTNPEDCKFTGNMFYNYMMGIEFNPRIGKWFDFKLFFNGRPGIVAWTLINLSY
AAKQQELYGYVTNSMILVNVLQAVYVVDFFWNEAWYLKTIDICHDHFGWYLGWGDCVWLPFLY
TLQGLYLVYNPIQLSTPHAAGVLILGLVGYYIFRVTNHQKDLFRRTEGNCSIWGKKPTFIECSYRS
ADGAIHKSKLMTSGFWGVARHMNYTGDLMGSLAYCLACGGNHLLPYFYIVYMTILLVHRCIRDE
HRCSNKYGKDWERYTAAVSYRLLPNIF-

Codon-optimized DNA sequence for studies in *S. cerevisiae*:

atgatggcgtcagaccgtgtcaggaagcgtcacaaaggtagcgctaatggtgcacaaaccgtagagaaggagccctccaaagaa
ccagcgcaatggggtagggcttgggaggtagattggttttcattgagcggtgtcatccttctgctatgcttcgcaccatttttggtctttttcttt
atcatggcttgcgatcagtatcaatgcagcatctcacatccgttgctagacctgtataatggcgatgcaaccctgttcacaatctggaatc
gtgctccgtctttcacatgggctgccgctaaaatttacgctatatgggtaaccttccaggtcgtactgtacatgtgcgtccccgacttccttc
ataagatcttgccgggatatgtcgggggagtccaagatggggcaaggacaccggcaggtcttattaacaagtatgaagttaatgggc
tgcaatgttggctaattacacacgttctgtgggtcttaaatgctcaacatttccactggttctctcccacaataatcattgataattggatacc
acttttatggtgtactaatatccttggttacgcagtttcaacgttcgcgttcataaaggcctatttatttccgacgaacccagaggattgtaag
tttacagggaacatgttttataactacatgatggggattgagttcaatccgaggattggaaagtggttcgactttaaactgtttttaatggaa
ggccgggtattgtggcctggaccctaataaacttatcttatgcagcgaaacaacaggaattatatgggtacgtcacaaattcaatgata
ctggtcaatgttctacaagcggtctacgtagtggacttcttctggaacgaggcatggtatcttaaaaccatcgatatatgccatgaccactt
tgggtggtatcttggttgggggattgtgtgtggctaccattcctatatactctgcagggcctgtatttagtatacaaccctatccagttgagt
accccacatgcggcgggagtgcttatcctgggtttagtaggttattacatattcagagtaacaaatcaccagaaagacctattcaggag
aacggaaggaaattgttcaatctggggcaaaaaaccaaccttcatagaatgctcctatagatctgcagacggagctattcataagtcc
aaacttatgacaagtggtttttggggagttgctcgtcacatgaactatacaggcgatctgatggggtctcttgcatattgcctagcttgcgg
gggtaatcatctattaccatactttacatagtttatatgactatattattggtgcaccgttgcatcagggatgaacacaggtgttccaataag
tacggaaaagactgggagcgttacacagccgccgtgtcctataggctactaccgaacatattctaa

Abbreviated protein name:

*Dr*DHCR24

Amino acid sequence:

MDPLLYLGGLAVLFLIWIKVKGLEYVIIHQRWIFVCLFLLPLSVVFDVYYHLRAWIIFKMCSAPKQH
DQRVRDIQRQVREWRKDGGKKYMCTGRPGWLTVSLRVGKYKKTHKNIMINMMDILEVDTKRK
VVRVEPLANMGQVTALLNSIGWTLPVLPELDDLTVGGLVMGTGIESSSHIYGLFQHICVAFELVL
ADGSLVRCTEKENSDLFYAVPWSCGTLGFLVAAEIRIIPAQKWVKLHYEPVRGLDAICKKFAEES
ANKENQFVEGLQYSRDEAVIMTGVMTDHAEPDKTNCIGYYYKPWFFRHVESFLKQNRVAVEYI
PLRHYYHRHTRSIFWELQDIIPFGNNPLFRYVFGWMVPPKISLLKLTQGETIRKLYEQHHVVQDM
LVPMKDIKAAIQRFHEDIHVYPLWLCPFLLPNQPGMVHPKGDEDELYVDIGAYGEPKVKHFEATS
STRQLEKFVRDVHGFQMLYADVYMERKEFWEMFDGTLYHKLREELGCKDAFPEVFDKICKSAR
H-

Codon-optimized DNA sequence for studies in *S. cerevisiae*:

atggatcctttgctatacttgggtgggttggcggtcttgtttctgatctggattaaggtcaagggactggaatatgtcataattcaccagagg
tggatctttgtgtgtctatttctattgccgttatccgtagtattcgacgtgtattatcaccttagagcctggatcatatttaagatgtgttctgcccc
gaagcaacatgaccaacgtgtaagggacatccagaggcaggttagggagtggaggaaggatggtggcaagaaatatatgtgcac
aggaagacccgggtggcttacggtatcattacgtgtaggcaaatacaaaaaaacgcacaaaaatatcatgatcaatatgatggatat
tctagaggttgatactaaaaggaaagtggtgagagtcgagcccttggcgaatatggggcaggtaacggcattattaaactccattggtt
ggaccctgccggtcctgcccgagttagacgacttaactgtcggtggcttggtaatgggtacgggtatagagtccagttcccatatctacg
gtctttttcaacatatttgtgtcgccttcgaattggtgttagcggatggaagtttagtaagatgtactgaaaaagaaaatagcgatctttttat
gctgtcccttggagctgtgggacgttaggcttccttgtagccgctgaaatcagaataatcccagctcagaagtgggtcaagttgcattatg
agccggtcagaggtctagacgccatttgtaagaaatttgccgaagagtccgcaaataaagagaatcaattcgttgagggcctacagt
actctcgtgacgaagcggtgattatgaccggcgtcatgacggatcatgccgagccagataagaccaattgtattggatactactacaa
accatggtttttcaggcatgttgagtcattcctaaaacaaaacagggtagccgtcgaatacataccattaagacactactaccaccgtc
acactagatccatattttgggagttacaagatatcattccctttgggaacaaccccctttttcgttatgtatttggctggatggtcccacccaa
aataagcctacttaaactgacccaaggcgagactattagaaagttatacgagcaacatcatgtggtgcaagacatgcttgttcccatg
aaagacattaaggccgccattcagcgtttccacgaggatattcatgtctatcccctttggctatgcccattcttactacctaatcagccggg
catggttcatcccaaaggtgatgaggatgaactatatgtagatataggggcgtatggagagccaaaggtcaagcattttgaagcaact
tctagtacaagacagttggagaaatttgttagggacgttcacggtttccagatgctatacgccgacgtctatatggagagaaaggaattt
tgggagatgtttgacggcactttgtaccataagcttagggaggaactgggttgcaaagacgcctttcccgaggtctttgataaaatctgta
agtctgcgcgtcattag

Abbreviated protein name:

*St*DHCR7

Amino acid sequence:

MVENKLVHSPLITYGSMLSLLSFTPPFVILMWYTNEHADGSILKTFNHLRENGLQGLIDIWPKPTA
IAGKLIICYALFEAALQLLLPGKTVEGPISPTGHRPVYKANGMAAYAVTLITYISLWWFGIFNPAIVY
DHLGEILSTLIFGSLVFCVLLYIKGHVAPSSTDSGSSGNIIVDFYWGMELYPRIGKHFDIKVFTNCR
FGMMSWAVLAVTYCIKQHEEYGRVSDSMLVNTILMLVYVTKFFWWEAGYWNTMDIAHDRAGF
YICWGCLVWVPSIYTSPGMYLVKQPVNLGLQLALYILVAGLLCIYINYDCDRQRQEFRRTNGKCT
VWGKTPSKIVAAYTTTSGEKKTSLLLTSGWWGLARHFHYVPEILAAFFWSVPALFNHFIPYFYVI
FLIILLLDRAKRDDDRCKAKYGKYWKLYCEKVPYRVIPGIY-

Codon-optimized DNA sequence for studies in *S. cerevisiae*:

atggtggagaacaagttagtccactccccgctgattacgtatgggtccatgttgtctctgctttcatttacacctccttttgtgatattaatgtgg
tatactaatgaacacgccgacggctctatcctgaaaacgttcaatcatttacgtgaaaacggtctacagggacttattgatatatggccc
aaaccaacagctattgcgggtaagctaataatatgctacgcgttatttgaggcggctttgcagctattgctgcccggcaaaacggtcga
gggaccaatatccccgacgggccacaggcctgtttataaagccaatggcatggcggcttacgcagtcacactgatcacctatatatcc
ctatggtggtttggcatttttaaccctgctatagtatacgaccacctgggtgaaatactgagcacgcttatattcggaagccttgtgttctgtg
tgctgttgtacattaaggggcacgtcgccccgtcaagtaccgactccgggtccagcggcaatataatcgtagacttctactggggaatg
gagttgtacccgaggatagggaagcacttcgatataaaggtttttaccaactgccgtttcggtatgatgagctgggctgttctagcagtca
catattgtattaagcagcacgaggaatatggcagagtgtctgatagtatgctagtcaacaccatcctaatgttagtatatgtgacgaaatt
cttttggtgggaggcgggatactggaatactatggacatcgcacacgatcgtgcaggattctacatctgttggggctgccttgtgtgggtc
ccctctatctacactagccccggaatgtatcttgttaaacagccggtgaacttgggattacagctagcactttacatactggtagcgggg
cttttgtgtatttatatcaattatgattgtgacagacagagacaggaattcaggcgtaccaatggcaaatgcacggtttggggtaagaccc
cgtctaaaatcgtagccgcttatacaactacgagtggtgaaaagaaaacgtcacttctactaacaagtggatggtggggcttggctag
acacttccattatgttcccgagatccttgcagcattcttttggagcgtccctgcattatttaatcattttattccttacttttatgtcatattcctgatc
attttgctgctagacagagcgaagcgtgacgatgataggtgtaaggcaaaatacggaaagtactggaagctatactgtgagaaagta
ccctatagggtcattccgggcatctactaa

Abbreviated protein name:

*Gg*DHCR24

Amino acid sequence:

MSAVWSLGAGLLLLLLWVRHRGLEAVLVHHRWIFVCFFLMPLSILFDVYYQLRAWAVRRMHSAP
RLHGQRVRHIQEQVREWKEEGGRRYMCTGRPGWLTVSLRVGKYKKTHKNIMINLMDVLEVDS
ERQVVRVEPLVTMGQLTAYLNPMGWTIPVVPELDDLTVGGLIMGTGIESSSHIYGLFQHTCMAY
ELVLADGSLVRCSPTENSDLFYAVPWSCGTLGFLVAAEIKMIPAKKYIRLHYEPVRGLRSICEKFT
EESKNKENSFVEGLVYSLEEAVIMTGVLTDEAEPSKINRIGNYYKPWFFKHVEKYLKANKTGIEYI
PSRHYYHRHTRSIFWELQDIIPFGNNPVFRYLFGWMVPPKISLLKLTQGEAIRKLYEQHHVVQD
MLVPMKSLEKSIQTFHVDLNVYPLWLCPFLLPNNPGMVHPKGDETELYVDIGAYGEPKTKQFEA
RASMRQMEKFVRSVHGFQMLYADCYMTREEFWDMFDGSLYHSLREQMNCKDAFPEVYDKIC
KAARH-

Codon-optimized DNA sequence for studies in *S. cerevisiae*:

atgagtgcggtatggtccttgggtgcaggactgcttctgttattattgtgggtaagacaccgtggtttagaggcggtgttagttcatcacag
gtggatatttgtttgcttctttcttatgccgttgtccatcctgttcgacgtatactatcaattaagagcttgggccgtccgtaggatgcatagcgc
ccccaggctgcatgggcaaagggtaagacatatccaggagcaggtcagagaatggaaggaggaaggcggacgtaggtacatgt
gtaccggacgtccggggtggctgactgtgtccttgagagttggaaaatataaaaaaacacataaaaacattatgataaatttgatggat
gttcttgaggtggatagcgagaggcaagtggttagggttgaaccgcttgtaaccatgggacaacttacggcatatttaaaccctatggg
ctggacaattccggtggtgcctgaacttgacgaccttactgtaggaggactgatcatgggaaccgggattgaatcttcaagtcatatata
tggtttattccaacatacgtgcatggcttacgaattagtattagcggatggctcattggtgaggtgtagccctacggagaattccgacctttt
ttacgcggtaccttggagctgcggtacactgggcttcttagtggcagctgaaataaagatgattccagcaaagaagtatataaggcttc
actacgaacctgtgaggggattgcgtagtatatgtgaaaagtttacggaggagtccaaaaataaagaaaattcattcgtggaaggtct
agtttatagcctagaggaggctgttataatgactggtgttctgacggatgaagcagaaccgtccaagattaatagaatcgggaactatt
acaagccatggtttttcaaacatgtagaaaaatacttaaaagctaataaaacgggtatagagtacataccgtcaagacattattaccat
cgtcatactagatccatattttgggagttacaggatataattccttttggtaataacccggtgttccgttacctatttggatggatggttccacc
caaaatatccctgctaaaactaacccaaggtgaggccataagaaaattgtacgaacaacatcacgtagtccaggatatgctagtccc
aatgaaaagtttagaaaaatctatccaaacattccacgtcgaccttaatgtttatccactgtggctatgtccatttttgcttccaaacaaccc
aggcatggtccatccgaaaggagatgaaactgaattgtacgtcgatattggtgcctacggagaacctaaaactaaacaattcgaagc
tagggcgtctatgaggcaaatggaaaaattcgtgaggagtgtgcacgggtttcaaatgctgtacgccgactgctatatgacacgtgaa
gagttctgggatatgttcgatggatcactatatcattctctaagggagcagatgaactgcaaagatgccttccctgaagtgtatgataaaa
tctgcaaggcggccagacattaa

Abbreviated protein name:

*Vc*CYP90B27v1

Amino acid sequence:

MAMELLLLIPAFIVAIIIFFSFKSTNGTSTKPLKLPPGQMGWPFIGHTIPFMQPHSSASLGPYIDLN
TARYGTIFRMNLLAKPTIVSADPEFNRYILQNEGRLFENSCPTSIAEIMGRWSMLALTGDVHREM
RSIAVSFMSNVKLRTYFIGDIEQQAIKVLASWAGRDAPFSAQDEGKKFAFNLMVKHLMSMEPGM
KETEQLRSEYHAFMKGMASIPINLPGTAYRKALQSRSIILKIMGEKLDERIKQVKEGCEGLEQDD
LLASVSKHPNLAKEQILDLILSMLFAGHETSSAAIALAIYFLESCPKAVEQLREEHKEIARQKKER
GETGLNWDDYKKMEFTHCVINETLRMGNIVKFLHRRAIKDVQFKGYDIPCGWEVVPIISAAHLD
SSIYDDPQRYDPWRWQAILAGNTKNNNVTSIMSFSGGPRLCPGAELAKLEIAVFLHHLVQKYQ
WEMAEHDYPVSFPFLGFPKRLPIKVRPLGD-

Native DNA sequence cloned directly from *V. californicum* for studies in *N. benthamiana*:[1]

atggcgatggagctcttattgttgatccctgcgacgttgatcgtcgccatcatcatcttcttcagcttcaaatcgacaaacgggacgtcgac
caaaccgctcaaactcccgccgggccaaatgggttggcctttcatcggccacaccatcccсttcatgcagccccactcctctgcatccc
ttggtccctacatcgacctcaacaccgctaggtatgggactatctttcggatgaacttgttggcgaaaccgacgattgtgtcggcggatc
cggagttcaaccggtacatactgcagaacgagggccggcttttccagaacagctgcccgacgagcatcgcggagatcatggcccgt
tggtcgatgctcgcgctcaccggagacgtccaccgcgaaatgaggtccatcgccgtcagcttcatgagtaacgtcaagctccgaactt
acttcatcggcgacatcgagcagcaggcccttaaagtcctcgcctcgtgggccggacgagacgctcccttctcggcccaagatgaag
gaaaaaagtttgcattcaatctaatggtgaagcatctaatgagcatggaaccgggcatgaaggagaccgagcagttaaggagcga
atatcacgctttcatgaaggggatggcgtcgatccccatcaacttgcccggcaccgcctacagaaaagcgttgcagtcgaggtccata
atcctgaagatcatgggcgagaagctcgacgagcggatcaagcaagtgaaggaaggctgcgagggcctcgagcaggacgacct
cctcgcctcggtctccaagcatcccaacctcgccaaggagcagattctcgacctcattctcagcatgcttttcgccgggcacgaaacct
cttctgccgccatcgccctcgccatctacttcctcgagtcttgccctaaagccgtcgagcagcttcgggaggagcacaaggagatcgc
cagacagaagaaggaacgcggagaaaccgggctcaactgggatgactacaagaaaatggagttcacccattgtgtcatcaatga
aaccctaagattggggaacattgtgaagttcttgcataggagagccatcaaggatgtgcagttcaaagggtatgacatcccatgtgggt
gggaagtggttccgatcatctcagccgcccatctggactcctcgatctacgacgacccacagcggtacgatccttggagatggcagg
cgattttggctggaaataccaagagcaacgcgatgtcaatcatgtcattcagcggcggaccccggctttgccctggcgccgagctggc
gaagctggagatcgccgtattccttcaccacctcgtccagaagtatcggtgggagctggcggagcacgattaccccgtgtcgttcccgt
tcctcggcttccccaagcgcttaccgatcaaagttcgcccсctcggagactaa

[1]This gene for studies in *N. benthamiana* transient was clonded directly from *V. californicum* and is 98% identical to the published sequence for *Vc*CYP90B27v1.

Codon-optimized DNA sequence for studies in *S. cerevisiae*:

atggcaatggaactattactgctaattcccgcttttatcgtggctataatcatctttttcagtttcaagtcaactaacgggacatcaactaaac
ccttaaagcttccccctggtcaaatgggttggccgtttataggacataccatcccctttatgcaaccgcactcttccgccagccttggacc
ctatattgatctaaacacagctaggtatgggacaatatttcgtatgaaccttctggcaaaacccaccatcgtttcagcagaccccgaatt
caataggtatatacttcagaacgaaggaagattatttgaaaattcctgccccacgtctatcgctgaaataatgggtaggtggtcaatgct
agccctaacaggcgacgtgcatcgtgaaatgcgttccattgctgtttcatttatgagtaatgtgaagctaaggacctatttcataggagat
atagagcagcaggccatcaaggtgcttgcaagttgggccgggcgtgatgcgcctttcagcgcacaggatgagggcaagaagtttgc
tttcaaccttatggtgaagcatcttatgagtatggaacctgggatgaaggagacagagcaattgagaagtgaataccacgcctttatga
aaggtatggcgagtatccccattaaccttccgggcacggcataccgtaaagcgctgcaatcacgttcaattatactgaaaattatgggc
gaaaaattagacgaaaggattaagcaggtgaaggagggatgcgaggggttagagcaagacgatttgcttgcgagtgtaagcaag
catccgaatttagcgaaggagcagattttggatttgatactgtctatgttattcgcaggccatgagaccagttctgccgccatcgccttagc
gatatactttttagagtcctgtccgaaagctgttgagcaactaagagaggaacacaaggagattgctagacagaagaaagagagag
gtgaaaccggtttgaattgggatgattacaaaaagatggagttcacccactgcgtaattaatgagacactgcgtatgggcaatatagta
aagttccttcatcgtagagcaattaaggacgtacagttcaaggggtatgacattccatgcgggtgggaggtagttcctatcatctcagca
gcccaccttgacagctcaatctacgatgacccacagaggtacgacccttggaggtggcaggcgatattggcgggcaacactaaaa

ataataatgtaacgtctattatgtctttcagcggaggaccgagactatgtccaggagcggagctggctaaacttgaaatagctgtcttctt
acatcatcttgtacagaaataccagtgggagatggcagaacacgactatccagttagttttcccttttttaggatttccaaagcgtttaccca
tcaaggttaggcccttgggcgattaa

Abbreviated protein name:

*Ec*CPR

Amino acid sequence:

MEQTAVKVSLFDLFSSILNGKLDPSNFSSDSSAAILIENREILMILTTAIAVFIGCGFLYVWRRSSNK
SSKIVETQKLIVEKEPEPEVDDGKKKVTIFFGTQTGTAEGFAKALAEEAKARYEKAIFKVIDLDDY
GADDDEFEEKLKKETIALFFLATYGDGEPTDNAARFYKWFTEGKEREMWLQNLQFGVFGLGN
RQYEHFNKVAKEVDEILTEQGGKRIVPVGLGDDDQCIEDDFTAWRELVWPELDQLLLDESDKTS
VSTPYTAIVPEYRVVFHDATDASLQDKNWSNANGYTVYDVQHPCRANVVVKKELHTPVSDRSC
IHLEFDISGTGLTYETGDHVGVYSENCVEVVEEAERLLGYSSDTVFSIHVDKEDGSPISGSALAP
PFPTPCTLRTALTRYADLLNSPKKAALHALAAYASDPKEAERLRYLASPAGKDEYAQWIVASQRS
LLVVMAEFPSAKAPIGVFFAAVAPRLLPRYYSISSSNRMVPSRIHVTCALVHEKTPAGRVHKGVC
STWMKNSVSLEENHDCSSWAPIFVRQSNFKLPADSTVPIIMIGPGTGLAPFRGFMQERLALKNS
GVELGPAILFFGCRNRQMDYIYEEELNNFVKEGAISEVVVAFSREGATKEYVQHKMAEKASYIW
EMISQGAYLYVCGDAKGMARDVHRTLHTIAQEQGSLDNSKTESLVKNLQMDGRYLRDVW-

Codon-optimized DNA sequence for studies in *S. cerevisiae*:

atggaacaaacggctgttaaagtaagtctgttcgacctgtttagttccattcttaacggaaaacttgatcctagtaatttctcaagtgactcc
agtgcagccatattgattgagaatagggagatcctaatgattcttacaacggccatagctgtgtttatcggctgcggattcttatacgtatg
gcgtagatcctctaataaaagtagtaaaattgtagaaacgcagaaattaattgtggagaaggagccagagcctgaagtcgacgacg
ggaaaaagaaagtgacgatctttttggtacccaaactgggactgcggaaggatttgcgaaagctcttgcagaagaggcgaaggcc
agatacgagaaagcgatctttaaggtcattgacctagatgactacggagccgatgatgatgagtttgaagaaaaattaaagaaggaa
actatcgctttattctttctagccacgtatggggatggagaaccaactgataatgctgccagattttataagtggttcactgagggcaaag
agagagaaatgtggttacagaatttacagttcggcgtgtttggtttgggaaacagacaatacgagcactttaataaggtcgctaaggag
gttgacgaaatactaacggagcaagggggcaaaagaattgtcccagtcggcttaggggacgacgatcagtgtatagaggacgattt
cacagcgtggcgtgaacttgtatggcctgagttagatcaattgttactagacgagtcagacaagacttcagtcagcactccgtataccg
cgatcgttcctgaatatcgtgtggtcttccacgatgcgacagacgcatcactacaagataagaattggtcaaatgctaatggctatacgg
tctacgatgtacaacacccatgccgtgcgaatgtagtcgttaagaaggagctgcacacgcccgtaagcgatcgtagctgcatccattt
ggaatttgacatatccgggactggtctgacctatgaaaccggagaccatgtaggggtatactctgaaaactgtgttgaggtggtcgagg
aagctgaacgtctgcttggttacagtagtgacaccgtgttctctatacatgtggacaaagaggatgggtcacctatctccggtagtgcact
tgcgccgccttttcctactccctgcaccctaaggacggcactgactaggtacgcggatcttcttaatagcccaaaaaaagctgcattgc
atgcgttggcagcgtacgcgtccgatcccaaagaggctgaacgtttaagatacttagcttcaccggcaggtaaggatgagtacgctca
atggattgttgcctcacaaagaagtctactggtcgtgatggctgaattcccttctgcgaaggcgcccataggtgtatttttcgcggccgtgg
caccgaggttacttcctaggtactactccattagttcttccaacaggatggtcccaagcaggatacatgttacatgtgccttggtgcatga
gaaaactcccgcaggcagggtgcataagggtgtgtgttccacgtggatgaaaaattcagtctcacttgaggaaaaccacgattgctc
aagttgggcccccatattcgtaaggcaatctaactttaagctacccgccgattccacagttccgataatcatgatcggccccggcacag
gcctggctccgtttcgtggctttatgcaggaaaggctggctttgaaaaattcaggtgtggagctgggtccggccatattattcttcgggtgc
agaaatagacagatggactatatctacgaagaagaacttaataacttcgtcaaggaaggcgcaataagtgaggtagtagttgcattct
ccagagaaggggctactaaggagtacgtacaacacaagatggcggaaaaggccagttatatatgggagatgatttcccaaggtgc
atacctttacgtgtgtggtgacgcgaaaggcatggctagagacgtgcatcgtacgttgcataccatcgctcaggaacaaggctctttgg
acaacagtaaaacagagagcctggttaaaaatctgcaaatggacggacgttacttacgtgatgtctggtaa

Abbreviated protein name:

*Vc*CYP90B27v1–mCherry

Amino acid sequence:

MAMELLLLIPAFIVAIIIFFSFKSTNGTSTKPLKLPPGQMGWPFIGHTIPFMQPHSSASLGPYIDLN
TARYGTIFRMNLLAKPTIVSADPEFNRYILQNEGRLFENSCPTSIAEIMGRWSMLALTGDVHREM
RSIAVSFMSNVKLRTYFIGDIEQQAIKVLASWAGRDAPFSAQDEGKKFAFNLMVKHLMSMEPGM
KETEQLRSEYHAFMKGMASIPINLPGTAYRKALQSRSIILKIMGEKLDERIKQVKEGCEGLEQDD
LLASVSKHPNLAKEQILDLILSMLFAGHETSSAAIALAIYFLESCPKAVEQLREEHKEIARQKKER
GETGLNWDDYKKMEFTHCVINETLRMGNIVKFLHRRAIKDVQFKGYDIPCGWEVVPIISAAHLD
SSIYDDPQRYDPWRWQAILAGNTKNNNVTSIMSFSGGPRLCPGAELAKLEIAVFLHHLVQKYQ
WEMAEHDYPVSFPFLGFPKRLPIKVRPLGDGSAGSAAGSGEFMVSKGEEDNMAIIKEFMRFKV
HMEGSVNGHEFEIEGEGEGRPYEGTQTAKLKVTKGGPLPFAWDILSPQFMYGSKAYVKHPADI
PDYLKLSFPEGFKWERVMNFEDGGVVTVTQDSSLQDGEFIYKVKLRGTNFPSDGPVMQKKTM
GWEASSERMYPEDGALKGEIKQRLKLKDGGHYDAEVKTTYKAKKPVQLPGAYNVNIKLDITSH
NEDYTIVEQYERAEGRHSTGGMDELYK-

Codon-optimized DNA sequence for studies in *S. cerevisiae*:

atggcaatggaactattactgctaattcccgcttttatcgtggctataatcatctttttcagtttcaagtcaactaacgggacatcaactaaac
ccttaaagcttccccctggtcaaatgggttggccgtttataggacataccatcccctttatgcaaccgcactcttccgccagccttggacc
ctatattgatctaaacacagctaggtatgggacaatatttcgtatgaaccttctggcaaaacccaccatcgtttcagcagaccccgaatt
caataggtatatacttcagaacgaaggaagattatttgaaaattcctgccccacgtctatcgctgaaataatgggtaggtggtcaatgct
agccctaacaggcgacgtgcatcgtgaaatgcgttccattgctgtttcatttatgagtaatgtgaagctaaggacctatttcataggagat
atagagcagcaggccatcaaggtgcttgcaagttgggccgggcgtgatgcgcctttcagcgcacaggatgagggcaagaagtttgc
tttcaaccttatggtgaagcatcttatgagtatggaacctgggatgaaggagacagagcaattgagaagtgaataccacgcctttatga
aaggtatggcgagtatccccattaaccttccgggcacggcataccgtaaagcgctgcaatcacgttcaattatactgaaaattatgggc
gaaaaattagacgaaaggattaagcaggtgaaggagggatgcgaggggttagagcaagacgatttgcttgcgagtgtaagcaag
catccgaatttagcgaaggagcagattttggatttgatactgtctatgttattcgcaggccatgagaccagttctgccgccatcgccttagc
gatatactttttagagtcctgtccgaaagctgttgagcaactaagagaggaacacaaggagattgctagacagaagaaagagagag
gtgaaaccggtttgaattgggatgattacaaaaagatggagttcacccactgcgtaattaatgagacactgcgtatgggcaatatagta
aagttccttcatcgtagagcaattaaggacgtacagttcaaggggtatgacattccatgcgggtgggaggtagttcctatcatctcagca
gcccaccttgacagctcaatctacgatgacccacagaggtacgacccttggaggtggcaggcgatattggcgggcaacactaaaa
ataataatgtaacgtctattatgtctttcagcggaggaccgagactatgtccaggagcggagctggctaaacttgaaatagctgtcttctt
acatcatcttgtacagaaataccagtgggagatggcagaacacgactatccagttagttttccctttttaggatttccaaagcgtttaccca
tcaaggttaggcccttgggcgatgggagcgccggtagtgcagcaggttccggggagtttatggtaagtaaaggtgaagaggacaat
atggccattattaaagaatttatgcgtttcaaagtacacatggaagggtctgttaacggtcatgagtttgagatagagggcgaggggga
agggcgtccgtacgaaggaacacaaaccgcaaagctgaaagtaacgaaagggggcctctaccatttgcttgggatattttgtcccc
gcaattcatgtatgggagcaaagcgtacgtcaagcatcccgccgacatacctgattacctaaaacttagttttcctgaaggcttcaaatg
ggaaagagtgatgaatttcgaagacggcggcgtagtcacggtcacacaggatagttccttacaggatggtgagttcatatacaaagt
aaagttgcgtgggactaatttcccctctgatgggccagtaatgcaaaaaaagacgatggggtgggaggcttctagtgaacgtatgtac
ccggaggacggggctctgaagggggagatcaaacagaggttaaaacttaaagacgggggtcactacgacgccgaagttaaaact
acctataaggctaagaagccagtgcaattacccggagcctataatgtgaacattaaattagacataacatcacataacgaagattac
actatagttgagcaatatgaaagagctgagggcagacatagtacgggaggaatggacgaactttacaaatag

Abbreviated protein name:

*Pp*CYP90B27

Amino acid sequence:

MALELILVLSSLIVILIIFFSFKSNGKSENKLAKLPPGQMGWPFIGQTIPFMQPHSSASLGLFMDQ
NIAKYGRIFRTNLLAKPTIVSADPDFNRYILQNEGRLFENSCPTSIKEIMGPWSMLALAGDIHREM
RSIAVNFMSNVKLRTYFLPDIEQQAIKVLASWENTPEAFSAQEQGKKFAFNLMVKHLMSMDPG
MPETEKLRTEYHAFMKGMASIPLNLPGTAYRKALQSRSIILKIMGQKLDERIRQVRDGCEGLEQ
DDLLASVSKHPHLTKEQILDLILSMLFAGHETSSAAIALAIYFLDSCPKAAQQLREEHVEIARQKA
ERGETGLNWDDYKQMEFTHCVINETLRLGNIVKFLHRKTLKDVQFKGYDIPCGWEVVTIISAAH
LDPSVYDEPQRYNPWRWQNISATASKNNSIMSFSGGPRLCPGAELAKLEMAVFLHHLVRKFHW
ELAEHDYPVSFPFLGFPKGLPIKVRPLEKSEA-

Codon-optimized DNA sequence for studies in *S. cerevisiae*:

atggctttggagttaattttggtcttgagcagccttatagttatattgatcatatttttctcattcaagtctaatggtaaaagcgagaataaactg
gccaaattaccacccgggcaaatgggctggcctttcattggtcagactattccattcatgcaaccccattcatcagcgtcattgggtttattt
atggaccaaaatatagcgaaatatgggcgtatttttaggaccaacctgttagcaaaaccgacgatagtaagtgcggatccggacttca
acagatacattctacagaatgaaggtaggttattcgagaactcctgccctaccagtataaaggaaatcatgggaccttggtctatgttag
cacttgcaggggatatacaccgtgagatgaggagtattgcggtaaattttatgtctaacgtcaagttaagaacctactttttaccggatatt
gaacagcaggcaataaaagtgcttgcgtcttgggagaacactcctgaggctttttcagcgcaggagcaggggaagaagtttgctttca
atttaatggtaaagcatctgatgtccatggaccccgggatgcctgagacggagaagctacgtacagagtaccatgcttttatgaaagg
catggcaagtatcccgctgaatctgcccggtacagcatacagaaaggctttgcagtcacgtagcatcattcttaagatcatgggacaa
aagctggacgaacgtattaggcaggtgagggacggatgcgaagggttggaacaggacgatttgttggccagtgttagtaaacatcc
acacttgacaaaagagcagattctagatcttattttgtcaatgttattcgcagggcacgaaaccagttctgcggcaattgccttggcaattt
attttttagactcttgtccaaaagcagcacagcagttaagagaagagcacgttgaaattgcgcgtcaaaaggcagaacgtggcgaga
caggtttgaattgggacgattacaaacaaatggaattcacacattgcgttataaatgaaaccttaaggcttggtaatattgtaaagtttctg
cataggaagactttgaaggatgtccaatttaagggctatgacataccgtgtgggtgggaagtggtgactattatcagtgcagcgcattta
gaccctagcgtttatgacgaacctcaacgttacaacccctggcgttggcaaaatatatccgcgaccgcatctaagaacaatagcattat
gtccttttctggtgggcccaggctgtgtccgggggctgagttagccaagttagaaatggctgtctttttacaccatctggttcgtaaattccat
tgggaattggcagaacacgattatccagtcagtttccccttccttggtttccctaaaggtttacctattaaggtcagaccacttgagaaaag
tgaggcatga

Abbreviated protein name:

*Ar*CYP71D443

Amino acid sequence:

MEFTYYFSLFLLFLLSCFVFVLIFSKTKQNLPPGPRKLPIIGHLHHLAGTAPPHHTLRHLADKHGP
LMHLQLGECGYVIASSTEIATHFFKTHDALFASRPSILASEIGAYNNTDISFAPYGKFWRQLRRIC
SVELLSAKRVKSFQPVREEEATELCKWIAQREGSAINLGEKVQQMNYHIMGRAVLGKKTGEQA
AFIALVKEGLDLMSGLDIVDLYPSYRILRLFSRLKRRIEKHHHAMDRIAHNIIEDRKRSDNGEHRD
HDLLDVLLGLQDDKSLEIPLTTDNIKAVLGDLFGAGVETSSTTVEWAMAEMLKHPKVLKKAQDE
VRMVFDAKNGVVDECYFDELKYLKLIVKESLRLHPPGPLLLPRVSSERCEINGYEIPAKTRLLVN
VYAIARDPKCWEDGESFKPERFLEKSVDFMGSSIELIPFGAGRRICPGITFGVATVEIALAMLLYY
FDWVLPEGMKAEDLDMTDWPGIAARKKDNLWAVPLVRRTLPA-

Codon-optimized DNA sequence for studies in *S. cerevisiae*:

atggaattcacctactactttagcctttttttacttttcctattgtcctgtttcgtgttcgttttgattttcagtaaaacaaagcagaatttgcctcccg
gcccacgtaaactacctattatagggcatctgcatcacctggcggggacagcacctcctcatcatacgttgcgtcaccttgctgacaaa
catggaccgttgatgcatttacaacttggtgaatgtggatacgttatagcttcatccactgaaattgcgactcatttttttaagacccatgac
gctctgttcgcctcccgtccttccatactagcaagtgaaatcggggcctacaacaatacggatatatccttcgcaccctacggcaagtttt
ggaggcaactaagaaggatctgtagtgtggagcttctgtccgctaaacgtgtcaaaagttttcagcctgtaagggaggaagaggcaa
ctgaactttgcaaatggatcgcacagagggaagggtccgctataaatcttggcgagaaggtgcagcaaatgaattaccacattatgg
gtagggctgttctgggcaagaagacgggagagcaagctgcgttcattgcgcttgtgaaggaagggctggatcttatgtctgggctaga
catcgttgacctttacccgtcataccgtatactgaggcttttttcacgtctgaagaggagaattgagaagcatcatcatgccatggatagg
atagcccataatatcatagaagatagaaagcgttctgataatggggagcatcgtgatcacgacttactagatgtacttttgggattacaa
gatgataaatcactggaaatcccccctaaccactgataatataaaagctgtgctaggcgatttgttcggcgcaggcgttgaaactagtag
cacaactgttgagtgggcaatggccgagatgttgaaacatcccaaggtcctgaaaaaggcacaggacgaggtgagaatggtgttcg
acgcgaagaacggggtagttgatgaatgctactttgatgaattgaagtatcttaagttgatcgtcaaagaatccttacgtctgcatcctcc
cggacctttgctgcttcccagggtatcctccgagagatgtgaaataaacggctacgagatacccgcaaagactagactactggttaac
gtatatgccatcgctagggaccctaaatgttgggaagatggagagtcttttaagccggagagatttttagaaaagagcgtggactttatg
ggtagctctatcgagttaattccatttggcgcgggcaggagaatctgtcccgggattacgttcggcgtggcaacagtcgagattgctttg
gccatgttactatattatttcgattgggtcctgccagagggtatgaaggcggaagacctagatatgactgattggcccggtatcgccgca
agaaagaaagataatctttgggcggttcccctagtcagaagaactcttccggcctaa

Abbreviated protein name:

*At*CYP90B1

Amino acid sequence:

MFETEHHTLLPLLLLPSLLSLLLFLILLKRRNRKTRFNLPPGKSGWPFLGETIGYLKPYTATTLGD
FMQQHVSKYGKIYRSNLFGEPTIVSADAGLNRFILQNEGRLFECSYPRSIGGILGKWSMLVLVG
DMHRDMRSISLNFLSHARLRTILLKDVERHTLFVLDSWQQNSIFSAQDEAKKFTFNLMAKHIMS
MDPGEEETEQLKKEYVTFMKGVVSAPLNLPGTAYHKALQSRATILKFIERKMEERKLDIKEEDQ
EEEEVKTEDEAEMSKSDHVRKQRTDDDLLGWVLKHSNLSTEQILDLILSLLFAGHETSSVAIALAI
FFLQACPKAVEELREEHLEIARAKKELGESELNWDDYKKMDFTQCVINETLRLGNVVRFLHRKA
LKDVRYKGYDIPSGWKVLPVISAVHLDNSRYDQPNLFNPWRWQQQNNGASSSGSGSFSTWG
NNYMPFGGGPRLCAGSELAKLEMAVFIHHLVLKFNWELAEDDKPFAFPFVDFPNGLPIRVSRIL-

Codon-optimized DNA sequence for studies in *S. cerevisiae*:

atgttcgagaccgagcatcatactttgttaccactgctgctgttgccttctctgctgtctttacttttatttctgatcctattgaaacgtaggaatc
gtaagactcgtttcaacctgccacctggcaaatccggctggccgtttttaggtgaaaccataggataccttaaaccgtacactgccaca
actttaggggactttatgcagcaacacgtcagcaagtacgggaaaatctatagatctaatctttttggagaacccacaatcgtcagtgcc
gatgcgggacttaacagattcatactacaaaacgagggaagactattcgagtgctcatatcctaggagcataggcgggatccttgga
aagtggtcaatgttggttttggtaggtgatatgcatcgtgacatgaggagcatctcactaaacttcctgagccatgctcgtctgcgtactat
attactgaaagacgtggaaaggcatactctatttgtacttgatagctggcagcaaaatagcattttctcagcacaagacgaagctaaga
aatttacgttcaacctgatggccaaacacattatgtccatggatccgggggaagaggaaaccgaacagctaaaaaaagagtacgtg
acatttatgaaaggtgttgtttctgccccattaaacttgcctggaactgcataccacaaagcattacagtcccgtgcaaccatattaaagtt
tattgagaggaagatggaggaaaggaaactggacattaaggaagaggatcaagaggaagaggaggttaaaaccgaggacgag
gcagagatgagcaagagcgaccatgtaaggaaacagagaactgatgacgatctattgggctgggtgttgaagcactctaaccttag
caccgaacaaattctagatcttatactgtctttattgtttgcagggcacgagacttcctcagtagcgattgcgttggcaatattcttcctgcag
gcgtgtcctaaagcagtcgaagagttgagggaggaacatcttgaaattgccagagcgaagaaagaattaggtgagtctgaattgaat
tgggatgactataagaaaatggactttacacaatgcgttataaacgagacccttagacttggaaacgtcgtgagattccttcacagaaa
ggcattaaaagacgtaaggtacaaagggtatgatattcctagcggatggaaggtgttacctgtgattagcgcagtacacctggataac
agccgttatgaccagccgaatttgtttaacccttggagatggcagcaacagaataatggagccagcagttccggctccggttccttttct
acctgggggaataactacatgcccttcggtggtggacctagactgtgcgcgggatctgaacttgccaagctagaaatggctgtattcatt
caccacctggtcttaaagttcaactgggaactggctgaggatgataaacctttcgcctttccgttcgtcgattttccgaacggattgccgat
acgtgtgtccaggatattataa

Abbreviated protein name:

*Os*CYP90B2

Amino acid sequence:

MAAMMASITSELLFFLPFILLALLTFYTTTVAKCHGGHWWRGGTTPAKRKRMNLPPGAAGWPLV
GETFGYLRAHPATSVGRFMEQHIARYGKIYRSSLFGERTVVSADAGLNRYILQNEGRLFECSYP
RSIGGILGKWSMLVLVGDPHREMRAISLNFLSSVRLRAVLLPEVERHTLLVLRAWPPSSTFSAQH
QAKKFTFNLMAKNIMSMDPGEEETERLRREYITFMKGVVSAPLNLPGTPYWKALKSRAAILGVI
ERKMEERVEKLSKEDASVEQDDLLGWALKQSNLSKEQILDLLLSLLFAGHETSSMALALAIFFLE
GCPKAVQELREEHLGIARRQRLRGECKLSWEDYKEMVFTQCVINETLRLGNVVRFLHRKVIKD
VHYKGYDIPSGWKILPVLAAVHLDSSLYEDPQRFNPWRWKSSGSSGGLAQSSSFMPYGGGTR
LCAGSELAKLEMAVFLHHLVLNFRWELAEPDQAFVFPFVDFPKGLPIRVHRIAQDDEQE-

Codon-optimized DNA sequence for studies in *S. cerevisiae*:

atggctgcgatgatggcgagcatcacgtcagagctgctgttctttttacccttcattttgttagcacttctaactttctataccactaccgtcgct
aaatgtcatgggggccattggtggagagggggaactacgcccgcaaagcgtaaaaggatgaatctgccgccaggagcagccgg
ctggcctcttgtcggcgagaccttcggatacctgagggctcatccggcaacttccgtaggcagatttatggagcagcatatcgccagat
atggaaaaatataccgtagttctttgtttggtgaacgtaccgttgtctctgcagacgcgggactgaaccgttatatactacaaaacgaag
gccgtctgttcgaatgtagttacccaaggtctataggtggtatattaggaaagtggagtatgctggttcttgtgggtgaccctcacagga
aatgagagcgatctccttaaattttttgtcctcagttaggcttagagcggtattacttccggaggtggaaaggcacaccttgcttgttctaag
agcttggccgccgtcttcaacattctctgcacagcaccaagccaagaagtttacctttaatttgatggccaagaacattatgtctatggat
cctggtgaggaagaaaccgaacgtttgaggagagaatacatcacttttatgaagggggtggtttctgcgcctttaaatctgccaggtac
gccgtattggaaggcgctaaagagcagggctgctattcttggcgtaatagaacgtaaaatggaagagagagtggagaaattgtctaa
agaggacgctagtgttgagcaagatgacctgcttggatgggcactgaagcagtccaatctttccaaggaacagatccttgatctgttgc
tgtccttacttttcgccggacacgagacaagctccatggccttggccttggctattttttttttggaggggtgtcctaaggcagtccaggagtt
aagagaagagcatttaggcatagccaggcgtcaacgtttaagaggagaatgtaaactgtcctgggaggactacaaggagatggtct
tcacgcagtgcgtgataaatgagacgcttagactaggcaatgtggtccgtttcctacatagaaaagtcataaaggatgttcactacaag
ggctacgatattccgtccggatggaaaatcttgcccgtcttagcggcagtgcacctggattctagtttgtatgaagatccccaaagattca
atccttggagatggaaatcaagtggctcttcaggagggctagctcaatcttctagtttcatgccctacggaggcgggacccgtctgtgtg
ctggttccgaacttgccaagttagaaatggcggtttttcttcatcacctggtattaaactttaggtgggagcttgcggagcccgatcaagcg
ttcgtatttccatttgtggacttccctaaggggctgcctatacgtgtccataggatcgctcaagacgatgaacaagagtaa

Abbreviated protein name:

*Sl*CYP90B3

Amino acid sequence:

MSDLEFFLFLIPPILAVLIILNLFKRKHNFQNLPPGDMGWPFLGETIGYLRPYSATTIGDFMQDHIS
RYGKIFKSNLFGEPTIVSADAGLNRYILQNEGRLFECNYPRSIGGILGKWSMLVQVGQMHRDMR
MISLNFLSNARLRNQLLSEVEKHTLLVLGSWKQDSVVCAQDEAKKLTFNFMAEHIMSLQPGNPE
TEKLKKEYITFMKGVVSAPLNFPGTAYRKALQSRSTILGFIERKMEERLKEMNRNENDLLGWVL
KNSNLSKEQILDLLLSLLFAGHETSSVAIALSIFLLESCPAAVQQLTEEHLEISRAKKQSGETELNW
DDYKKMEFTQCVINETLRLGNVVRFLHRKAVKDVRYKGYDIPCGWKVLPVISAAHLDPSLFDRP
HDFDPWRWQNAEESPSGKGGSTGTSSTTKSSNNFMPFGGGPRLCAGSELAKLEMAIFIHYLV
LNFHWKLAATDQAFAYPYVDFPNALPINIQHRSLNKLHD-

Codon-optimized DNA sequence for studies in *S. cerevisiae*:

atgtcagatttagaattctttttgttcttaattccgcccatcctagctgtcctaataattcttaacctattcaagaggaagcacaatttccagaa
cttaccacccggagatatgggctggcctttcctgggagagactattggatacctgaggccctattctgccaccacgataggagacttcat
gcaggaccacatcagtaggtatggtaagattttcaaaagcaatctgtttggtgagcctaccatcgtctctgctgatgctggactgaaccgt
tacattctgcaaaacgagggccgtttattcgaatgcaattatccgagatcaatagggggcattctaggtaaatggtcaatgcttgtacag
gttggtcagatgcacagggatatgaggatgatatcactaaactttttgtctaacgctcgtctgagaaaccaacttttgtctgaggtcgaaa
aacatacactgctagtgctgggcagttggaaacaggactctgttgtgtgtgctcaggacgaagcgaagaagctaacttttaacttcatg
gcagagcatattatgtccctgcagcctggaaatcctgaaaccgagaaattaaagaaggagtatatcacgtttatgaaaggcgtggtct
ccgcccctctgaatttccctgggacggcttatcgtaaggcgctacagagtcgtagtaccatactgggattcattgaaaggaagatggag
gagagattaaaagagatgaacaggaacgagaacgatttacttggatgggtattgaaaaactccaatctttcaaaggagcaaatcctt
gatttactattaagccttctattcgcagggcacgagacttctagcgtggctatagccttgtcaatcttcctacttgaatcttgtccggcagca
gtccaacagctaaccgaagaacatctggaaatttcaagagccaaaaaacaaagcggtgagactgagctaaattgggacgattaca
agaagatggagttcacccaatgtgtcataaatgagactctgcgtttgggtaatgtagtaaggttccttcatcgtaaggctgtgaaggatgt
tcgttacaaaggctatgacattccttgcggctggaaagtgctacctgtaattagcgcagctcacttggatccaagtctgttcgatagaccc
catgacttcgatccttggaggtggcaaaatgcggaagagtcaccgagtgggaagggaggtagcacgggaacttcaagcacaaca
aagtcctctaataattttatgccgtttggaggtggtccacgtctatgtgctgggtctgaattggcgaaactggagatggccatatttatccatt
atcttgttttaaactttcactggaagttggctgccacagaccaagctttcgcctatccgtatgtagattttccgaatgctcttccgataaacat
acaacatcgttcccttaacaagcttcatgactaa

Abbreviated protein name:

*Tf*CYP90B51

Amino acid sequence:

MSDSDITFYCLSSILSVLLIFIFILIKRKQAKPKLNLPPGKMGWPFLGETIGYLKPYSATTLGEFMD
QHIARYGKIYKSKLFGEPAIVSADAGLNRFILQNEGKLFECSYPRSIGGILGKWSMLVLVGDMHR
DMRLISLNFLSHARLRTHLLKEVEKHTRLVISSWKENSTFAAQDEAKKFTFNLMAEHIMSLQPGK
IETEKLKKEYVTFMKGVVSAPLNFPGTAYWKALKSRGTILKFIEGKMEERIKRMKEGNENLEEDD
LLNWVLKHSNLSTEQILDLILSLLFAGHETSSVSIALAIYFLPGCPQAILQLREEHKEIARAKKQAG
ETELTWEDYKKMEFTHCVVNETLRLGNVVRFLHRKALKDVRYKGYDIPCGWKVLPVIAAVHLDP
LLFDQPQHFNPWRWQNNGNCPNFSGASSNSNNIFLPFGGGPRLCAGSELAKLEMAVFIHHLIL
NYHWELTDNNDQAFAYPFVDFPKGLQIRVQSHSLI-

Codon-optimized DNA sequence for studies in *S. cerevisiae*:

atgtccgactctgacataacctttattgtttgagttcaatcctgtcagttttgcttatttttattttcattctgattaaacgtaaacaagccaagcc
gaagctgaatctaccgcctggtaagatgggttggcccttttggggaaacaatagggtacttaaagccctacagtgcaacgacactg
ggggagtttatggaccagcatatagcgagatacggcaagatttacaagtccaagttgttcggcgaaccggcgatagtaagtgcagac
gcgggcctaaaccgtttcattttacaaaatgaaggcaagctttttgagtgtagttacccccgttctataggcgggattctgggtaagtggtc
tatgctggtgctggtcggagacatgcatagggacatgaggctgatcagcctaaatttcttatctcacgcccgtcttagaacgcatcttctta
aagaggttgaaaaacacacgcgtttggtaataagctcatggaaagagaacagtacgtttgcagcccaggacgaagcaaaaaaatt
tactttcaacttgatggcagagcatataatgtctttgcagcctggtaagattgaaacagagaaattaaaaaaagaatatgtaacgtttatg
aagggcgtagtaagtgctccgctgaacttccctggaaccgcgtactggaaggctcttaaaagccgtgggaccattttgaaattcataga
ggggaagatggaagaaaggataaaacgtatgaaggaagggaacgaaaaccttgaagaagatgatctattaaattgggtgcttaaa
cacagtaacttatctacggagcaaatattggacctaatactttccctactattcgcgggtcatgaaacatcatccgtttctattgcccttgca
atttactttctaccgggatgtccacaagcgatcttgcagctacgtgaggagcataaggagatagcgagggccaaaaaacaagcggg
ggagaccgagcttacgtgggaggattataagaaaatggagttcacacactgtgtggtgaacgaaacattacgtctggggaatgttgtg
agatttctgcatagaaaagctctgaaagacgtgcgttacaaaggctacgacatcccgtgtggttggaaggtattgcccgtcatagcgg
cggttcatctagatccgttgttgttcgatcagccacagcactttaacccgtggcgttggcaaaataacggaaactgccctaacttttcagg
cgctagtagcaactccaacaatatattcctacctttcggaggaggtcctagactatgcgcaggttcagagcttgccaaacttgagatgg
ccgtcttcatacaccatctaatcttgaactaccattgggaattaacggataacaatgaccaagcatttgcataccctttcgttgattttccca
aaggcttgcagattagggtccaaagccacagtctgatttaa

Abbreviated protein name:

*Pp*CYP90B52

Amino acid sequence:

MEGLLLLLPTAIIALYLYISLIRRSRKKHNLPPGSDGWPFLGETFSYLKPHSAISIGRFMEDHISRY
GKIYRSNLFGEPTIVSADAELNRFVLQNEGRLFECSYPRSIGGILGKWSMLVLVGDMHRDMRMI
SLNFMSAARLRTRLMPEVERQTLLVLRSWREGSTFSAQEEAKKFTFNLMAKHIMSMDPGEPET
EMLRREYITFMKGVVSAPLNFPGTPYWKALKSRSSILAVIERKMEERIGRRDRGDGGVEDDDLL
GWAMNQSNLLKEQILDLLLSLLFAGHETSSMALALAIYFLESCPEAVRDLRDEHLAISMSGKEGE
CGLSWDQYKQMEFTHCVINESLRLGNVVRFVHRKAIQDVQYKGYDIPCGWKVLPVFAAVHLDS
TLYSDPHRFNPWRWQSSSSKTTAANFMPYGGGLRLCTGSELAKLEMAVFLHHLVLNYQWKLA
EPEQAFAYPFLDFPKGLQIKVRAIT-

Codon-optimized DNA sequence for studies in *S. cerevisiae*:

atggaaggattgctactgttgctgccaactgccattattgctttgtacttatatatatctctaatcaggagaagtaggaagaaacacaacct
tccccccggcagcgacggctggccttttctaggggaaacattctcatacttaaaacctcactccgcaatatcaattgggagatttatgga
agaccatatttccagatacggcaaaatatatcgtagtaatcttttcggcgaaccaaccatcgtttctgcagacgctgaattaaatcgtttcg
tcttgcagaatgaggggcgtttgttcgagtgttcttaccctagaagcataggcggcatactggggaagtggtcaatgcttgtattagtggg
agatatgcaccgtgacatgcgtatgatcagtttgaatttcatgtctgcagctaggttacgtaccaggctaatgcccgaagtcgagagac
aaacacttttggtcctgcgtagctggagggagggcagtacgttcagtgcccaggaggaagctaagaagttcactttcaaccttatggct
aaacacattatgtccatggatccaggcgaaccagagacagaaatgttgcgtcgtgagtatattactttcatgaaaggagtcgtgagcg
ccccttttaaactttccaggtacaccgtattggaaagcactaaaatctaggtcaagcatactggccgtgatagagagaaaaatggaag
agagaatcggaagacgtgatcgtggcgacggtggagtggaggatgacgaccttcttggatgggcgatgaaccaaagcaatttactt
aaagaacagattctggatttactattgtctcttttgtttgcgggtcacgaaactagttccatggctttagcactagcgatctactttctggaaa
gttgcccagaggccgtcagagacctaagagatgagcacttggcaattagcatgagcggaaaggaaggggagtgcggtttaagctg
ggaccaatataaacagatggaatttactcactgtgtgattaatgaaagtcttcgtttaggtaacgtggtgaggtttgtccatagaaaagcc
atacaagatgtgcagtacaaaggatacgatatcccatgcggatggaaagtattacctgtgtttgcagcggtgcatctggatagcaccct
atactcagacccccataggttcaatccttggcgttggcagtcatctagttctaagactacggcggctaacttcatgccgtatggaggcgg
gctaagactttgtaccggctcagaactggcgaagttagagatggctgtttttcttcaccacctggtgcttaactaccaatggaaattagca
gaacccgagcaagctttcgcctaccccttttttagacttcccaaaaggcttacagattaaagtcagggccattacttaa

Abbreviated protein name:

*Dz*CYP90B71

Amino acid sequence:

MAPMELLLIVSPLVLALIIFFSFRGTSKGSDKAEKIPPGTMGWPLIGHTIPFMQPHSSASLGLFVD
QNIAKHGRIFRMNLLGKPTIVSADADFNRFILQSEGRMFENSCPTSIAEIMGRWSMLALAGDVH
REMRSIAVNFMSNVKLRTYFLPDVEQQAIKILSAWRHGSTFSAQEEGKKFAFNLMVKHLMSMD
PGMPETEQLRKEYITFMKGMASIPLNLPGTAYRKALQSRSIILKIMGQKLDERVEKVKRGCEGLE
EDDLLASVAAQSNITRDQILDLILSMLFAGHETSSAAICLAIYFLESSPKALQQLREEHINIAKMKK
EKGETGLTWDDYKQMEFTHCVINETLRLGNIVKFLHRKAIKDVQYKGYDIPCGWEVVPIISSAHL
DPSIYDDPQSYNPWRWQTISTATSKNNNIMSFSGGPRLCPGAELAKMEMAVFLHHLVQKFNWE
LAEHDYPVSFPFLGFPKHLPIKVHAIDHKASA-

Codon-optimized DNA sequence for studies in *S. cerevisiae*:

atggcacctatggagctgttactgattgtctctccgctggtcctagctcttatcatcttcttcagtttccgtgggaccagtaaggggtctgata
aagcggaaaaaatccctccgggaacgatggggtggccgcttatcggccacacaattcccttcatgcaaccacactcctcagccagct
taggattgttcgttgatcagaacatcgcgaagcacggacgtatttttcgtatgaatctgttggggaaacccacaatcgtctccgctgacgc
tgattttaaccgttttatattgcagtccgaggggagaatgtttgagaatagttgtccaactagtatcgccgagataatgggcaggtggagc
atgctagctctagccggagacgtccacagggagatgcgtagcattgccgtaaatttcatgtccaatgttaaactaagaacgtactttttgc
cagacgtagagcagcaagcgattaagatactatccgcctggaggcacggtagtaccttctcagcacaagaagaaggtaagaagttt
gcattcaatttgatggtaaaacatttaatgagcatggaccctggtatgccagagaccgagcagttgagaaaggaatacataacctttat
gaaagggatggcttccatccccctaaaccttcctggtacggcttacagaaaggctttgcaatctaggagcatcattctaaagattatggg
gcaaaagttagacgaaagagtagaaaaggttaagcgtgggtgcgaagggttagaggaagatgatctactagcctcagtggcggct
caatctaatataaccagagaccagatcctggatctgatattatcaatgctgtttgccgggcacgaaacttccagcgcagccatatgtcta
gccatttatttttagagagcagcccaaaggctctacagcaactgagagaggagcatataaacatcgctaagatgaagaaggagaa
gggtgagactgggttaacatgggacgattacaagcaaatggaattcacccattgtgtgataaacgagaccctaaggctgggcaatatt
gtgaagttcttgcatcgtaaagctataaaggatgttcaatacaagggctacgacataccatgtggatgggaggttgtgcccatcatctca
tccgcccaccttgatccatccatctacgacgacccccagagctacaatccatggcgttggcagactatttctacggccacctctaagaa
caataatattatgtcttttagtgggggcccgagactttgtccgggcgccgagttagctaagatggagatggcggtctttttacatcacctag
tacagaaatttaattgggaattagcggaacacgattatccggtgtcattcccttttttagggtttccgaaacacctaccaattaaggtacat
gctattgatcataaagcatcagcataa

Abbreviated protein name:

*Ia*CYP708A15v2

Amino acid sequence:

MNLVWTAAFGAIALLVVKISQWCYRWSNPKCNGKLPPGSMGFPIIGETFDFFKPHDMYEISPFV
MKRMLRYGPIFRTNVLGLKTVVSTDTDVNHEILKQENTSFVFRMPGSFEKMLGKDSLAVNHGKI
HKHIKQITLNFLGSDSLKRNMIKEMDRLTRELLESKGAEGIFDVKDEVSRLVVRHLTPKMLSNLP
QKTQEKLMDDVKSFNYDWFRPHFTLATLKKLFRSLRGRRGVMKVIVDAFKKRRETKEKYGDFL
DTMLDNLDKDDSLLTEDSAQSLVFFLYFASHESTSTTTSLTVKLLSKHPRVLQELKKEHMAILES
RENKESGITYEEYRHKMIFTNMVINEVLRLINLAPLMFRMAVEDVEIKGYTIPAGWNVLVTPPMV
HYDPKVYENPMEFNPWRWEGKDVRSVSKTFMAFGGGIRQCAGADFARLQISLFLHYLVTNYN
FSLAKDNDVMRVLGLYSPKEMPINVSPFPK-

Codon-optimized DNA sequence for studies in *S. cerevisiae*:

atgaacctagtgtggacagcggcgttcggcgctatcgcattgttggtcgtcaaaattagccaatggtgttacagatggtctaatccgaag
tgtaacggcaaactgccgccaggaagtatgggatttccgattataggcgaaacgttcgactttttaagcctcatgacatgtacgagata
tcacccttcgtcatgaagagaatgttgagatatggtccgatttttaggacaaatgttcttggtttgaaaactgtagttagcacagatacgga
cgtcaaccacgagatcctaaaacaggagaatacaagcttcgtcttcaggatgccgggcagtttcgagaaaatgttaggaaaggact
cacttgccgtgaatcacggtaagatccacaagcatatcaaacaaattaccttgaacttccttggctcagattcattaaagaggaatatga
ttaaagagatggacagacttacccgtgagctattagagagcaaaggcgcggagggcatcttcgatgtgaaagatgaggtctcaagg
ctggttgtcagacatttaacacctaaaatgctgtccaatctaccccaaaaaactcaagaaaagctaatggatgatgtgaaatccttcaat
tacgactggtttcgtccccatttcacactagccactttaaaaaagctattcaggtctcttcgtggccgtagaggcgtcatgaaagtgatagt
tgatgcctttaagaaaagacgtgagactaaagaaaagtacggagactttctggacacgatgctagataatttagataaggatgactca
ctacttactgaggatagtgcccaatcattagtcttcttcttgtattttgcgagccacgaatcaacaagtacgacaaccagtctaacggtca
aactgttatcaaagcaccctagagtactgcaggaactaaaaaaagaacacatggcgatcctagaatcaagggaaaacaaggaat
cagggatcacttatgaagaatacaggcacaaaatgattttcacaaacatggttattaacgaagtgctgaggcttattaacttagcccccct
tgatgttcagaatggccgtggaggacgtcgagatcaaggggtacactatccccgccggatggaacgtgttagtgacaccgcctatggt
tcattacgatccgaaggtgtacgagaatcccatggaatttaacccctggagatgggagggcaaagatgtaagatctgtcagtaaaact
tttatggccttcgggggtggtatccgtcaatgcgctggggcagattttgcaaggttacagatttcactgttccttcattacctagtgaccaatt
ataacttctctcttgcaaaggataatgacgtgatgcgtgtgttaggtttgtatagcccgaaggaaatgccgatcaacgttagcccgtttccc
aagtaa

Abbreviated protein name:

*At*CYP724A1

Amino acid sequence:

MGWPFIGETISFFKPHRSDSIGTFLQQRVSRYGKVFKSNICGGKAVVSCDQELNMFILQNEGKL
FTSDYPKAMHDILGKYSLLLATGEIHRKLKNVIISFINLTKSKPDFLHCAENLSISILKSWKNCREV
EFHKEVKIFTLSVMVNQLLSIKPEDPARLYVLQDFLSYMKGFISLPIPLPGTGYTNAIKARKRLSA
RVMGMIKEREREEEDMNNAIREEDFLDSIISNEDLNYEEKVSIVLDILLGGFETSATTLSLVVYFLA
KSPNLLHKLKEEHAAIRAKKGDGELLNWEDYQKMEFTQCVISEALRCEYVIPKGWKVFPIFTAV
HLDPSLHENPFEFNPMRWTDKAKMNKKTTAFGGGVRVCPGGELGKLQIAFFLHHLVLSYRWKI
KSDEMPIAHPYVEFKRGMLLEIEPTKFLED-

Codon-optimized DNA sequence for studies in *S. cerevisiae*:

atgggctggccgttcataggagagacaataagcttcttcaagccccaccgttccgattcaatcggcactttcttacaacaaagggtctct
aggtacggcaaagtattcaaatcaaatatatgtggcggcaaggctgttgtgagctgcgaccaagagttgaacatgttcatactacaga
acgaagggaaattattcacgtctgattatccgaaagccatgcacgacattctgggaaagtattctctattactagcgaccggggagatt
cacagaaagcttaaaaatgtcataataagttttattaaccttactaaaagtaagcctgacttcttgcattgcgcagagaaccttagtatatc
aattctaaaatcatggaagaactgcagggaggtggagttccacaaagaagtgaaaatttttacgctaagtgtcatggttaatcaactac
ttagcataaagcctgaagaccccgcaaggctgtacgtgcttcaagatttcttatcttatatgaagggattcatttcacttcccatccccctac
ctggaacagggtacactaacgcaatcaaagccaggaagagattatctgcgagggtcatgggtatgatcaaggagagggagcgtg
aagaagaagatatgaataacgcaatacgtgaggaggatttcttagattctattatatccaatgaagacttaaattacgaagaaaaagt
aagcattgttctggatattttactagggggggtttgagacctctgccacaacgttgagcctggtggtgtattttcttgctaagagtcccaacctt
ttacataagctgaaagaggaacacgctgccataagggccaaaaaaggcgacggcgaacttttaaattgggaggattatcagaaaa
tggagttcacgcagtgtgttatcagtgaagcacttcgttgtgagtatgtcatacctaaaggatggaaggtcttcccaatattcacagctgtg
catctggatccctccttgcacgaaaatccgttcgagttcaatcctatgcgttggacagacaaggcgaagatgaacaagaaaacaacc
gcgtttggaggcggagttcgtgtatgccctggtggcgaactgggaaagcttcaaattgcatttttcttacatcacttggtcttatcatatcgtt
ggaaaataaagagcgatgaaatgccaatagcacatccttatgtggagttcaaacgtgggatgttattggaaattgagccaactaaatt
ccttgaagactaa

Abbreviated protein name:

*Os*CYP724B1

Amino acid sequence:

MVGGELVLAALVILLALLLTLVLSHFLPLLLNPKAPKGSFGWPLLGETLRFLSPHASNTLGSFLED
HCSRYGRVFKSHLFCTPTIVSCDQELNHFILQNEERLFQCSYPRPIHGILGKSSMLVVLGEDHKR
LRNLALALVTSTKLKPSYLGDIEKIALHIVGSWHGKSKDKGMVNVIAFCEEARKFAFSVIVKQVLG
LSPEEPVTAMILEDFLAFMKGLISFPLYIPGTPYAKAVQARARISSTVKGIIEERRNAGSSNKGDFL
DVLLSSNELSDEEKVSFVLDSLLGGYETTSLLISMVVYFLGQSAQDLELVKREHEGIRSKKEKDE
FLSSEDYKKMEYTQHVINEALRCGNIVKFVHRKALKDVRYKEYLIPSGWKVLPVFSAVHLNPLLH
GNAQQFQPCRWEGASQGTSKKFTPFGGGPRLCPGSELAKVEAAFFLHHLVLNYRWRIDGDDI
PMAYPYVEFQRGLPIEIEPLCSES-

Codon-optimized DNA sequence for studies in *S. cerevisiae*:

atggtcggaggggagttagtcttggctgcgctggtaattttactagccctgttactgacgcttgttttgtcacattttcttcctctactattgaatc
ctaaagcaccaaaaggttctttcggctggcccctactgggcgagacactaaggtttctgtctccacacgcctccaataccttaggcagc
ttccttgaggaccattgcagcagatatgggagagtctttaagagtcatctattctgcacaccgactattgtttcatgtgatcaggagttaaa
ccactttatcttacagaacgaagaaaggttatttcaatgtagttacccgagaccgatccacggcatattagggaaatcctccatgctggt
agttctaggggaagatcataaaaggcttaggaatttagcattggcgctggtgacatccacaaaactgaagccgagctacctgggga
catagaaaagatagcgttacacatagttggatcctggcacggtaaatcaaaagataaaggtatggtaaatgtgatagcattttgtgagg
aagccaggaaatttgcattttccgtaattgtaaagcaggtgttggggctttcccccgaggagcctgtcaccgccatgatcttagaggattt
cttggcatttatgaagggtttgattagttttcccctttacataccagggacgccgtacgcgaaggcagtgcaagcgagagcgcgtatctc
cagtaccgtaaagggcataattgaagaacgtaggaatgcgggaagcagtaacaaaggagactttctagacgttctgctttccagcaa
cgaattatcagatgaagaaaaggtttctttcgtacttgacagtttgctaggaggatatgagacaaccagtttattaatatcaatggtagtgt
attttttaggtcagtccgctcaagacttggagcttgtaaaacgtgaacatgagggcatcaggtctaagaaagaaaaggacgaatttctg
tcttctgaagattacaaaaaaatggagtacacacaacacgtcataaacgaagcattaagatgtggcaatatcgtcaagtttgtgcaca
gaaaggctttgaaggacgtaagatataaggagtaccttattccgtcaggttggaaggtgctgccggtcttcagcgcggtccatctaaat
ccgttgctacacggcaatgcacaacaatttcagccttgcaggtgggaaggagcctctcagggtaccagtaagaagtttactccttttgg
gggaggccccagactttgcccaggttcagagttggcgaaagttgaagcagctttcttcttacaccacctggtgttgaattataggtggag
gatcgacggggatgatattccgatggcgtatccttatgttgaattccaaaggggcctgcccattgaaatagagcctctttgcagtgaatcc
taa

Abbreviated protein name:

*Sl*CYP724B2

Amino acid sequence:

MGEEGSLLIIVITLVFSFVIGITLNHFWPLFFNNYGTTLHVIPKGTFGWPLLGETLSFLKPHPSNSI
GTFLQQHCSRYGKVFKSHLFFSPTVVSCDQDLNYFILQNEDKLFQCSYPKPIHGILGKVSLLVAV
GDTHKRLRNVSLSLISTIKSKPEFINDVETLALQILQSWKDKHQVRYWEEARKFSFNVIVKQVLG
LTPDNPQSALILQDFLAFMRGLISLPLYIPGTPYARAVQARSRISSTIKAIIEERRRKHVVDGDGKK
NDFLEILLCVDTLSEEEKVSFVLDSLLGGYETTSLLMAMVVFFIGQSQTAFDRLKEEHDNIRSTK
EKELLNWEDYQKMDFTQKVINEALRYGNVVKFVHRKALKDVKFKDYVIPAGWKVLPVFSAVHL
DPSVHPNALHFNPWRWESDEQISKKLTPFGGGSRCCPGFELAKVEVAFFLHHLVQKYRWEVE
EGEQPIAYPYVEFKNGLTIRLHKNST-

Codon-optimized DNA sequence for studies in *S. cerevisiae*:

atgggagaagagggaagcttacttatcatcgtgatcactctagtgttctcattcgtgatcggtattactttaaaccacttctggccgctgttctt
caacaactatggcactactttgcacgttatacccaaaggcaccttcgggtggcctttacttggcgaaaccctatcattcttgaagccacat
ccctctaacagcatcggcacatttctacaacagcattgcagtaggtatggcaaggttttcaaatcccacttgtttttcagccccacagtcgt
ttcctgtgaccaggatttgaactacttcatactacagaatgaagacaagctgttccaatgttcttaccccaagccaatacacgggattctt
ggcaaagtttctttgctagtggcggttggggacactcataaacgtctaaggaacgtcagcctgagtttgattagcacgatcaagagtaa
gcctgagttcattaacgatgtggaaactttggcgttgcagattttacaatcctggaaagacaaacaccaggtccgttactgggaggaag
caaggaagttttcatttaacgtcatcgttaagcaggttctgggtctgaccccagacaacccccaatccgcgttaatccttcaagattttctg
gcattcatgcgtggcttgatttcccttcctctgtacattcccgggacgccgtatgcaagagcggttcaagcaagatctagaatcagtagta
ccatcaaggctattatcgaagaaaggcgtaggaagcatgtagtggacggagatggaaagaagaacgatttttggaaatcctactat
gcgttgatacgttgagtgaggaggagaaagttagtttcgtgttagactcactgctaggcggctacgagaccacgagtcttctgatggcta
tggtagtgttcttcatcggacagtctcagactgcctttgacagattgaaagaagagcacgacaatatacgttctacgaaagagaagga
actgttgaattgggaagactaccagaagatggattttacgcagaaggtgataaacgaagcgttaaggtatggtaatgtagttaagttcg
ttcaccgtaaggcgctgaaggatgtcaagttcaaagattatgttatacctgcagggtggaaagtcttaccagtttttccgcggtccatctt
gatccgtcagtacacccaaatgctcttcacttcaacccttggaggtgggaaagcgacgaacaaatttccaagaagctaaccccgttcg
gagggggtctcgttgttgtccaggctttgaactggcaaaagtggaagtcgcgtttttttgcatcatctagtccaaaaataccgttgggaa
gtagaggaaggagagcagccgatagcctatccgtacgtcgaatttaagaatggcttgacaataaggttacacaaaaactcaaccta
a

Abbreviated protein name:

*Dz*CYP90B71–mCherry

Amino acid sequence:

MAPMELLLIVSPLVLALIIFFSFRGTSKGSDKAEKIPPGTMGWPLIGHTIPFMQPHSSASLGLFVD
QNIAKHGRIFRMNLLGKPTIVSADADFNRFILQSEGRMFENSCPTSIAEIMGRWSMLALAGDVH
REMRSIAVNFMSNVKLRTYFLPDVEQQAIKILSAWRHGSTFSAQEEGKKFAFNLMVKHLMSMD
PGMPETEQLRKEYITFMKGMASIPLNLPGTAYRKALQSRSIILKIMGQKLDERVEKVKRGCEGLE
EDDLLASVAAQSNITRDQILDLILSMLFAGHETSSAAICLAIYFLESSPKALQQLREEHINIAKMKK
EKGETGLTWDDYKQMEFTHCVINETLRLGNIVKFLHRKAIKDVQYKGYDIPCGWEVVPIISSAHL
DPSIYDDPQSYNPWRWQTISTATSKNNNIMSFSGGPRLCPGAELAKMEMAVFLHHLVQKFNWE
LAEHDYPVSFPFLGFPKHLPIKVHAIDHKASAGSAGSAAGSGEFMVSKGEEDNMAIIKEFMRFK
VHMEGSVNGHEFEIEGEGEGRPYEGTQTAKLKVTKGGPLPFAWDILSPQFMYGSKAYVKHPAD
IPDYLKLSFPEGFKWERVMNFEDGGVVTVTQDSSLQDGEFIYKVKLRGTNFPSDGPVMQKKTM
GWEASSERMYPEDGALKGEIKQRLKLKDGGHYDAEVKTTYKAKKPVQLPGAYNVNIKLDITSH
NEDYTIVEQYERAEGRHSTGGMDELYK-

Codon-optimized DNA sequence for studies in *S. cerevisiae*:

atggcacctatggagctgttactgattgtctctccgctggtcctagctcttatcatcttcttcagtttccgtgggaccagtaaggggtctgata
aagcggaaaaaatccctccgggaacgatggggtggccgcttatcggccacacaattcccttcatgcaaccacactcctcagccagct
taggattgttcgttgatcagaacatcgcgaagcacggacgtatttttcgtatgaatctgttggggaaacccacaatcgtctccgctgacgc
tgattttaaccgttttatattgcagtccgaggggagaatgtttgagaatagttgtccaactagtatcgccgagataatgggcaggtggagc
atgctagctctagccggagacgtccacagggagatgcgtagcattgccgtaaatttcatgtccaatgttaaactaagaacgtactttttgc
cagacgtagagcagcaagcgattaagatactatccgcctggaggcacggtagtaccttctcagcacaagaagaaggtaagaagttt
gcattcaatttgatggtaaaacatttaatgagcatggaccctggtatgccagagaccgagcagttgagaaaggaatacataacctttat
gaaagggatggcttccatcccccctaaaccttcctggtacggcttacagaaaggctttgcaatctaggagcatcattctaaagattatggg
gcaaaagttagacgaaagagtagaaaaggttaagcgtgggtgcgaagggttagaggaagatgatctactagcctcagtggcggct
caatctaatataaccagagaccagatcctggatctgatattatcaatgctgtttgccgggcacgaaacttccagcgcagccatatgtcta
gccatttatttttagagagcagcccaaaggctctacagcaactgagagaggagcatataaacatcgctaagatgaagaaggagaa
gggtgagactgggttaacatgggacgattacaagcaaatggaattcacccattgtgtgataaacgagaccctaaggctgggcaatatt
gtgaagttcttgcatcgtaaagctataaaggatgttcaatacaagggctacgacataccatgtggatgggaggttgtgcccatcatctca
tccgcccaccttgatccatccatctacgacgacccccagagctacaatccatggcgttggcagactatttctacggccacctctaagaa
caataatattatgtcttttagtgggggcccgagactttgtccgggcgccgagttagctaagatggagatggcggtctttttacatcacctag
tacagaaatttaattgggaattagcggaacacgattatccggtgtcattcccttttttagggtttccgaaacacctaccaattaaggtacat
gctattgatcataaagcatcagcagggagcgccggtagtgcagcaggttccggggagtttatggtaagtaaaggtgaagaggacaa
tatggccattattaaagaatttatgcgtttcaaagtacacatggaagggtctgttaacggtcatgagtttgagatagagggcgaggggga
agggcgtccgtacgaaggaacacaaaccgcaaagctgaaagtaacgaaagggggcctctaccatttgcttgggatattttgtcccc
gcaattcatgtatgggagcaaagcgtacgtcaagcatcccgccgacatacctgattacctaaaacttagttttcctgaaggcttcaaatg
ggaaagagtgatgaatttcgaagacggcggcgtagtcacggtcacacaggatagttccttacaggatggtgagttcatatacaaagt
aaagttgcgtgggactaatttcccctctgatgggccagtaatgcaaaaaaagacgatggggtgggaggcttctagtgaacgtatgtac
ccggaggacggggctctgaaggggggagatcaaacagaggttaaaacttaaagacgggggtcactacgacgccgaagttaaaact
acctataaggctaagaagccagtgcaattacccggagcctataatgtgaacattaaattagacataacatcacataacgaagattac
actatagttgagcaatatgaaagagctgagggcagacatagtacgggaggaatggacgaactttacaaatag

Abbreviated protein name:

*Aa*CPR

Amino acid sequence:

MQSTTSVKLSPFDLMTALLNGKVSFDTSNTSDTNIPLAVFMENRELLMILTTSVAVLIGCVVVLVW
RRSSSAAKKAAESPVIVVPKKVTEDEVDDGRKKVTVFFGTQTGTAEGFAKALVEEAKARYEKAV
FKVIDLDDYAAEDDEYEEKLKKESLAFFFLATYGDGEPTDNAARFYKWFTEGEEKGEWLDKLQ
YAVFGLGNRQYEHFNKIAKVVDEKLVEQGAKRLVPVGMGDDDQCIEDDFTAWKELVWPELDQL
LRDEDDTSVATPYTAAVGEYRVVFHDKPETYDQDQLTNGHAVHDAQHPCRSNVAVKKELHSPL
SDRSCTHLEFDISNTGLSYETGDHVGVYVENLSEVVDEAEKLIGLPPHTYFSVHTDNEDGTPLG
GASLPPPFPPCTLRKALASYADVLSSPKKSALLALAAHATDSTEADRLKFFASPAGKDEYAQWIV
ASHRSLLEVMEAFPSAKPPLGVFFASVAPRLQPRYYSISSSPKFAPNRIHVTCALVYEQTPSGRV
HKGVCSTWMKNAVPMTESQDCSWAPIYVRTSNFRLPSDPKVPVIMIGPGTGLAPFRGFLQERL
AQKEAGTELGTAILFFGCRNRKVDFIYEDELNNFVETGALSELVTAFSREGATKEYVQHKMTQK
ASDIWNLLSEGAYLYVCGDAKGMAKDVHRTLHTIVQEQGSLDSSKAELYVKNLQMAGRYLRDV
WVDMEQKLISEEDLE-

Codon-optimized DNA sequence for studies in *S. cerevisiae*:

atgcagtcaactacatcagtcaaattaagtccttttgatttgatgaccgctctgttaaacggaaaagtgtcatttgatacttcaaatacatct
gataccaacataccettggctgtattcatggagaatagggaattgttaatgattctgaccacgagtgtagccgtacttatcggttgtgttgtt
gttctggtatggaggagaagttcatccgctgctaagaaggccgcggaatcaccagtaatagtggttccgaaaaaggttacagaggac
gaagttgatgacggtcgtaaaaaggtgaccgttttcttcggaacccagactggtacggccgagggcttcgcgaaggcactggttgag
gaggcaaaggcgaggtatgagaaggctgtcttcaaggtcattgatctagacgactacgctgccgaagatgatgaatatgaagaaaa
actgaagaaggaatcattagcttttttttttctggccacctatggcgatggggaacccacagataatgcagctcgtttttataaatggttcac
agaaggcgaagaaaagggtgagtggctggataaactacagtatgccgtgtttggattaggtaatcgtcagtatgagcatttcaataaa
attgcgaaggtcgtggacgaaaaactggttgagcaaggggccaaaagacttgtaccagtcggaatgggtgatgatgatcaatgcatt
gaagatgactttacggcctggaaagaacttgtttggcccgagttagaccagttattaagggacgaagacgacacgtctgtcgcgacg
ccgtacacggctgcggtcggggaatatagagttgtatttcacgataagccggagacttacgatcaggaccagttaacaaatggacat
gcagtgcatgacgctcaacacccttgtaggtctaatgtcgccgttaaaaaggagctacactccccgttatcagacaggtcttgcacgca
cctagaattcgatatctcaaacacgggtttatcttacgagactggtgaccatgttggggtatatgtggagaacctatccgaagtggtaga
cgaagcggagaaattgattggcttaccgccccatacttacttttccgttcatactgataatgaagacggtactccccttggtggggcctctc
tgccaccaccctttccaccgtgcactctgcgtaaggctttggcatcatatgccgatgtcctatcatcccctaaaaaaagcgcacttctagc
gcttgccgcgcacgctacggacagcacagaagcagataggctaaagttcttcgcctctcctgcagggaaagacgaatacgcgcaat
ggattgtagcgagtcacagatctctattagaagtaatggaggcctttccatcagctaagcctcctctgggcgtattttttgcgtccgttgcac
ctcgtctacagccaaggtactattccataagttcaagtcctaagtttgcgccaaacaggattcacgttacttgcgcattggtctacgagca
aacaccaagcggtagggtgcacaaggggggtatgcagtacatggatgaaaaatgcggtgcccatgaccgagtcacaggactgttctt
gggcaccaatctacgtcagaactagtaattttaggctgcccagcgaccctaaggtgcctgtaattatgataggcccgggaacgggact
tgcaccgtttcgtgggttcctacaagagcgtctagcgcagaaagaggccgggactgagctggggacggcgattttattctttggctgta
gaaacaggaaggttgactttatttatgaggatgaattgaataacttcgtggagaccggcgctttgtcagaactagtgacagcgttttcca
gagaaggggctacgaaggaatatgtccaacacaaaatgactcaaaaggcgagcgatatttggaatctgttgagcgagggcgcata
tttatatgtctgcggcgacgctaaaggtatggcgaaggacgttcatcgtaccttacatacgatcgttcaggagcaggggagcttagact
ctagcaaagcggaattgtatgtcaagaatttacaaatggccggcagatacctgagagacgtttgggtagacatggaacaaaagctta
tttccgaagaggacttggaatga

Abbreviated protein name:

*At*CPR

Amino acid sequence:

MTSALYASDLFKQLKSIMGTDSLSDDVVLVIATTSLALVAGFVVLLWKKTTADRSGELKPLMIPKS
LMAKDEDDDLDLGSGKTRVSIFFGTQTGTAEGFAKALSEEIKARYEKAAVKVIDLDDYAADDDQ
YEEKLKKETLAFFCVATYGDGEPTDNAARFYKWFTEENERDIKLQQLAYGVFALGNRQYEHFN
KIGIVLDEELCKKGAKRLIEVGLGDDDQSIEDDFNAWKESLWSELDKLLKDEDDKSVATPYTAVIP
EYRVVTHDPRFTTQKSMESNVANGNTTIDIHHPCRVDVAVQKELHTHESDRSCIHLEFDISRTGI
TYETGDHVGVYAENHVEIVEEAGKLLGHSLDLVFSIHADKEDGSPLESAVPPPFPGPCTLGTGL
ARYADLLNPPRKSALVALAAYATEPSEAEKLKHLTSPDGKDEYSQWIVASQRSLLEVMAAFPSAK
PPLGVFFAAIAPRLQPRYYSISSSPRLAPSRVHVTSALVYGPTPTGRIHKGVCSTWMKNAVPAE
KSHECSGAPIFIRASNFKLPSNPSTPIVMVGPGTGLAPFRGFLQERMALKEDGEELGSSLLFFG
CRNRQMDFIYEDELNNFVDQGVISELIMAFSREGAQKEYVQHKMMEKAAQVWDLIKEEGYLYV
CGDAKGMARDVHRTLHTIVQEQEGVSSSEAEAIVKKLQTEGRYLRDVW-

Codon-optimized DNA sequence for studies in *S. cerevisiae*:

atgacgtcagctctgtatgcctcagacctgtttaaacagctgaagtcaatcatgggcacggacagcttaagtgacgatgttgtgcttgtg
atcgctacgacctcattagccttagtcgctggctttgtagtcctgctgtggaagaaaacgacggcggaccgttctggtgagcttaaaccc
ctaatgataccgaaatctcttatggccaaggacgaggatgatgatctggatttgggcagcggcaaaacacgtgttagtatattcttcggt
acccagacggggacggctgaagggtttgcgaaagccctgagtgaagaaatcaaagccagatacgagaaagccgcggttaaagt
gatcgatcttgacgattacgcggcggacgatgaccaatacgaagagaagctaaagaaagagacgctggcgtttttttgcgttgctacg
tatggggacggcgagccgaccgataacgcagcacgtttttacaagtggtttacagaggagaatgagagagacataaagctgcaac
aattagcctacggggtttttgctctgggaaataggcaatacgagcactttaacaagataggtatcgtgttggacgaagagctatgcaaa
aaaggcgctaaacgtttaatcgaggtgggtttaggagacgatgatcaatccatagaagatgatttcaatgcatggaaggagtccttgtg
gtcagagttagacaaattactaaaggacgaggacgacaagagtgttgccactccgtacacagcggtcatacctgagtacagggtag
tgactcacgatcccaggttcaccactcagaagtctatggagtccaatgtagccaacggcaataccacaatcgacatacaccatccttg
cagggtagacgtggctgtgcagaaggaattacacacccatgaatctgaccgttcatgcatccacttagagttcgatattagccgtaccg
ggattacctatgaaacaggtgatcacgtaggcgtctatgccgaaaaccacgttgaaatcgttgaggaagcaggaaaattactgggtc
acagtctggatttggtttttcaatacatgcggataaagaagacggttccccccttgagtctgcagttcccccaccttttccgggaccgtgta
cgttaggtactggccttgcaagatatgcggatctattgaatcccccccaggaaatctgcgctagtagctctggcagcctatgcgaccgaa
cccagcgaggcggaaaagttaaagcatctaacttcccctgacggcaaggacgagtacagtcaatggattgtggcgtcccaaagaa
gtctgctagaagtcatggcagcattcccttctgcgaaacccccctcttggggtgtttttgcagccatcgcccctcgtctacaaccgcgttact
acagcatctcatcttctcccagattggcaccaagccgtgtccacgttacgtctgcattggtgtatggcccgactccaaccggtaggatcc
ataagggtgtctgctctacctggatgaagaatgcggtgccagctgagaaatcacacgagtgcagcggcgccccgatttttatcagagc
gtcaaacttcaaattgcccagtaatccgagtacccctatcgtaatggttggccctgggacaggtctagcaccctttagaggtttcctacag
gagaggatggcattaaaggaagatggtgaagaacttggaagctctttactgttcttcgggtgccgtaataggcagatggatttcatatac
gaggatgagttaaacaatttcgtagatcaaggggttatatccgaactaattatggcattctcaagagagggcgcgcagaaggagtac
gtacaacataaaatgatggagaaggcggcgcaagtctgggacttaatcaaagaagaaggttatctttacgtctgcggagatgcaaa
aggaatggcaagagatgtgcacaggactttgcacacaatagttcaagaacaagaaggcgtcagttcctccgaagccgaagcgatc
gtgaaaaagttacagacagagggtaggtatttgcgtgatgtctggtga

Abbreviated protein name:

*Ps*CPR

Amino acid sequence:

MAPINIEGNDFWMIACTVIIVFALVKFMFSKISFYQSANTTEWPAGPKTLPIIGNLHQLGGGVPLQ
VALANLAKVYGGAFTIWIGSWVPMIVISDIDNAREVLVNKSADYSARDVPDILKIITANGKNIADCD
SGPFWHNLKKGLQSCINPSNVMSLSRLQEKDMQNLIKSMQERASQHNGIIKPLDHAKEASMRL
LSRVIFGHDFSNEDLVIGVKDALDEMVRISGLASLADAFKIAKYLPSQRKNIRDMYATRDRVYNLI
QPHIVPNLPANSFLYFLTSQDYSDEIIYSMVLEIFGLGVDSTAATAVWALSFLVGEQEIQEKLYREI
NNRTGGQRPVKVVDLKELPYLQAVMKETLRMKPIAPLAVPHVAAKDTTFKGRRIVKGTKVMVNL
YAIHHDPNVFPAPYKFMPERFLKDVNSDGRFGDINTMESSLIPFGAGMRICGGVELAKQMVAFA
LASMVNEFKWDCVSEGKLPDLSEAISFILYMKNPLEAKITPRTKPFRQ-

Codon-optimized DNA sequence for studies in *S. cerevisiae*:

atggcaccaatcaatatcgagggtaacgacttctggatgattgcttgtaccgtgataattgtattcgcactagtcaaatttatgttttcaaaa
atttccttttaccaatcagcaaataccactgaatggcctgctggtcctaaaaccctgccaatcatagggaatctgcaccagctaggtggc
ggggttcctctacaggtcgccctagcgaatttagccaaggtgtacggcggtgcatttacgatctggattggtagttgggtaccgatgattg
taatctcagatatagataacgcgagggaggtgctggtaaacaagtctgcggactactctgcgagagatgttccggacatcttgaagat
cataaccgccaatggaaagaatatcgccgactgcgatagtggaccattctggcataatctaaagaagggacttcaatcctgcattaat
ccgtctaacgttatgtctttatcaagactacaagagaaagatatgcaaaacctaatcaaatctatgcaagagagagcgagccaacac
aacgggatcattaagcccctagatcacgcgaaagaggcatcaatgcgtcttctttcaagggtcatcttcgggcacgacttcagtaacg
aagacttagttataggagtaaaagacgcacttgacgaaatggtaaggattagtgggctagcgtccttggccgacgcatttaaaatcgct
aagtaccttcctagccaacgtaagaacatcagggacatgtatgccaccagggacagagtctacaatctgattcagccccacatcgtc
cctaatctacctgccaacagtttcctttacttcctaaccagtcaggattatagtgacgagattatctatagcatggtcttagaaatattcgga
ctaggtgtcgacagcacggctgctaccgcggtgtgggctctaagttttcttgttggtgagcaggagatccaagaaaagctttacagaga
gatcaataataggactggaggtcaacgtccagtcaaggtcgttgatttgaaagaactgccttacctgcaagctgtcatgaaagaaactt
tgagaatgaaaccaattgccccccttagcagtcccacatgttgcggctaaagatacgacttttaagggaagacgtatcgtgaaggggga
ctaaggttatggttaacctgtatgcaattcatcacgatccgaatgtctttcctgcgccctacaaatttatgccagagaggttcttgaaagatg
tcaacagtgatggcaggtttggagatattaacacgatggagtccagcctgattccctttggcgcgggtatgagaatatgtggcggagtc
gagttggcaaaacagatggtagcctttgcgttggcttccatggtgaatgagttcaagtgggattgcgtcagcgagggaaagctaccag
atctgtccgaggcgattagcttcattttgtacatgaaaaacccccttagaggccaagattacgcctaggactaagccgtttcgtcaataa

Abbreviated protein name:

*Dz*CPR

Amino acid sequence:

MKLSALDLVTATLTGGEGIPLEAVGRERVLAMLATAVALLIGFVALLFWRRSAGQKQNRPAEPMK
PLVVRDEAEDVDDGKKRVTIFFGTQTGTAEGFAKALAEEARARYEKATFKIVDLDDYAADDDEY
EEKMKKETLALFFLATYGDGEPTDNAARFYKWFTEGKERGVWLQNLQYTVFGLGNRQYEHFN
KVAKVVDEILSDQGGKRLVPVGLGDDDQCIEDDFNAWKELVWPELDQLLRNEDDASGASTPYT
AVIPEYRVVFIDSTEASYLEKNWSLANGHSVHDIQHPCRANVAVKRELHTPASDRSCIHLEFDIA
GTGLGYETGDHVGVFSENCIETVEEAERLLGYSSDTFFSIHANQEDGSPRSGGSLAPPFPSPC
TLRTALSRYADLLSSPKKAALLALAAHASDPSEAERLKFLASPAGKDEYSQWIVASQRSLLEVMA
EFPSAKPPLGVFFAAISPRLQPRYYSISSSPKMAPSRIHVTCALVYGPTPTGRIHKGVCSTWMK
HAVPYEESQDCSWAPIFVRQSNFKLPADTSVPIIMIGPGTGLAPFRGFLQERLAMKKSGIELGHA
ILFFGCRNRKMDFIYEDELNNFVETGALSELIVAFSREGPTKDYVQHKMVEKASELWNIISKGGYI
YVCGDAKGMARDVHRVLHTIAQQQGSLDNSKAESMVKSLQMDGRYLRDVW-

Codon-optimized DNA sequence for studies in *S. cerevisiae*:

atgaaactgagtgccctggacctagtcacggctaccctgaccggtggcgaaggcatcccactggaggcagttggaagagagagag
tcctggccatgttagcgaccgccgttgccttacttattggctttgtggccttgttattttggaggcgttctgcaggtcaaaaacagaacagac
ccgctgagcctatgaaacctttagtggttagggacgaggctgaagacgttgatgacggaaaaaagagggttaccattttttggaacg
caaacgggaacagccgagggatttgcgaaggctcttgcagaagaagctagagcgagatatgagaaggcaacattcaagatagta
gaccttgacgattatgcggccgacgatgatgagtatgaggagaagatgaaaaaggaaacccttgccctgttctttttagcgacctacg
gggacggcgaacccacggataacgctgccaggttttacaagtggtttaccgagggtaaagaaagaggagtctggctgcagaactta
cagtacactgtttttggacttgggaatcgtcagtatgagcatttcaataaagtggcgaaagttgtagacgagattctaagtgatcaagga
ggcaagaggttagtcccggtcggacttggagatgatgatcagtgtatcgaggacgactttaacgcttggaaagaattggtttggcccga
gcttgaccagctgttacgtaacgaagacgacgcgagtggtgcctctaccccatatacagcagtgatccccgagtacagggtggtttta
ttgactctaccgaagcctcctacctagaaaagaattggtctctagccaatggtcattcagtccacgatattcaacacccgtgccgtgcaa
acgtggcggttaagagagagttgcacacgcccgcgagtgacaggtcttgcatccatttagagtttgacatagccggtaccgggttagg
atacgagactggggaccacgtcggtgtttttagcgagaattgcatcgagactgtcgaagaagctgaacgtctactgggatattctagtg
acacattcttctccattcatgccaaccaagaggacggctcccccaggagtggtgggagcctggcgcctccctttcctagtccctgcactt
taaggactgcactaagcagatatgcggaccttctgtcctctccgaaaaaagccgctctgttagcattagctgcccacgcgtcagaccc
atccgaagcggagcgtttgaagtttttggcgtcccctgcaggaaaagacgaatatagtcagtggattgtcgcgagtcaaagaagcctt
ctagaggtgatggccgaatttccaagtgcaaagcccccactaggagtcttcttcgcggcgatctcccccaggctgcaacctcgttatta
cagcatcagcagttccccgaagatggcccctctcgtatccatgttacttgcgccttggtctacggtcctactccgactggaaggatacat
aagggagtgtgctccacgtggatgaagcacgcggtaccttacgaggagagtcaggattgctcctgggcgccgattttcgtccgtcagt
ccaactttaagttaccggcggacacttccgttcccattatcatgataggtccggggacaggtctagccccattccgtgggtttttgcagga
gaggctggccatgaaaaagagcggtatcgagctgggccatgctattttgtttttggatgccgtaacaggaagatggactttatttatgaa
gatgagttaaacaacttcgtagaaaccggggcactttctgagttgatcgtagcgtttagcagggaagggccgacgaaggattacgtcc
aacacaagatggtagaaaaagcttcagaactatggaacatcatatccaaaggagggtacatttatgtttgcggagatgctaagggca
tggcgagagacgtgcatagagtccttcatactattgcgcaacagcagggaagtttagataattccaaggccgaatcaatggtcaaatc
cttacaaatggacggaaggtatctacgtgatgtctggtaa

Abbreviated protein name:

*At*CPR–mCherry

Amino acid sequence:

MTSALYASDLFKQLKSIMGTDSLSDDVVLVIATTSLALVAGFVVLLWKKTTADRSGELKPLMIPKS
LMAKDEDDDLDLGSGKTRVSIFFGTQTGTAEGFAKALSEEIKARYEKAAVKVIDLDDYAADDDQ
YEEKLKKETLAFFCVATYGDGEPTDNAARFYKWFTEENERDIKLQQLAYGVFALGNRQYEHFN
KIGIVLDEELCKKGAKRLIEVGLGDDDQSIEDDFNAWKESLWSELDKLLKDEDDKSVATPYTAVIP
EYRVVTHDPRFTTQKSMESNVANGNTTIDIHHPCRVDVAVQKELHTHESDRSCIHLEFDISRTGI
TYETGDHVGVYAENHVEIVEEAGKLLGHSLDLVFSIHADKEDGSPLESAVPPPFPGPCTLGTGL
ARYADLLNPPRKSALVALAAYATEPSEAEKLKHLTSPDGKDEYSQWIVASQRSLLEVMAAFPSAK
PPLGVFFAAIAPRLQPRYYSISSSPRLAPSRVHVTSALVYGPTPTGRIHKGVCSTWMKNAVPAE
KSHECSGAPIFIRASNFKLPSNPSTPIVMVGPGTGLAPFRGFLQERMALKEDGEELGSSLLFFG
CRNRQMDFIYEDELNNFVDQGVISELIMAFSREGAQKEYVQHKMMEKAAQVWDLIKEEGYLYV
CGDAKGMARDVHRTLHTIVQEQEGVSSSEAEAIVKKLQTEGRYLRDVWGSAGSAAGSGEFMV
SKGEEDNMAIIKEFMRFKVHMEGSVNGHEFEIEGEGEGRPYEGTQTAKLKVTKGGPLPFAWDI
LSPQFMYGSKAYVKHPADIPDYLKLSFPEGFKWERVMNFEDGGVVTVTQDSSLQDGEFIYKVK
LRGTNFPSDGPVMQKKTMGWEASSERMYPEDGALKGEIKQRLKLKDGGHYDAEVKTTYKAKK
PVQLPGAYNVNIKLDITSHNEDYTIVEQYERAEGRHSTGGMDELYK-

Codon-optimized DNA sequence for studies in *S. cerevisiae*:

atgacgtcagctctgtatgcctcagacctgtttaaacagctgaagtcaatcatgggcacggacagcttaagtgacgatgttgtgcttgtg
atcgctacgacctcattagccttagtcgctggctttgtagtcctgctgtggaagaaaacgacggcggaccgttctggtgagcttaaaccc
ctaatgataccgaaatctcttatggccaaggacgaggatgatgatctggatttgggcagcggcaaaacacgtgttagtatattcttcggt
acccagacggggacggctgaagggtttgcgaaagccctgagtgaagaaatcaaagccagatacgagaaagccgcggttaaagt
gatcgatcttgacgattacgcggcggacgatgaccaatacgaagagaagctaaagaaagagacgctggcgtttttttgcgttgctacg
tatggggacggcgagccgaccgataacgcagcacgtttttacaagtggtttacagaggagaatgagagagacataaagctgcaac
aattagcctacggggtttttgctctgggaaataggcaatacgagcactttaacaagataggtatcgtgttggacgaagagctatgcaaa
aaaggcgctaaacgtttaatcgaggtgggtttaggagacgatgatcaatccatagaagatgatttcaatgcatggaaggagtccttgtg
gtcagagttagacaaattactaaaggacgaggacgacaagagtgttgccactccgtacacagcggtcatacctgagtacagggtag
tgactcacgatcccaggttcaccactcagaagtctatggagtccaatgtagccaacggcaataccacaatcgacatacaccatccttg
cagggtagacgtggctgtgcagaaggaattacacacccatgaatctgaccgttcatgcatccacttagagttcgatattagccgtaccg
ggattacctatgaaacaggtgatcacgtaggcgtctatgccgaaaaccacgttgaaatcgttgaggaagcaggaaaattactgggtc
acagtctggatttggtttttcaatacatgcggataaagaagacggttccccccttgagtctgcagttcccccaccttttccgggaccgtgta
cgttaggtactggccttgcaagatatgcggatctattgaatccccccaggaaatctgcgctagtagctctggcagcctatgcgaccgaa
cccagcgaggcggaaaagttaaagcatctaacttcccctgacggcaaggacgagtacagtcaatggattgtggcgtcccaaagaa
gtctgctagaagtcatggcagcattcccttctgcgaaaccccctcttggggtgtttttgcagccatcgcccctcgtctacaaccgcgttact
acagcatctcatcttctcccagattggcaccaagccgtgtccacgttacgtctgcattggtgtatggcccgactccaaccggtaggatcc
ataagggtgtctgctctacctggatgaagaatgcggtgccagctgagaaatcacacgagtgcagcggcgccccgatttttatcagagc
gtcaaacttcaaattgcccagtaatccgagtacccctatcgtaatggttggccctgggacaggtctagcaccctttagaggtttcctacag
gagaggatggcattaaaggaagatggtgaagaacttggaagctctttactgttcttcgggtgccgtaataggcagatggatttcatatac
gaggatgagttaaacaatttcgtagatcaaggggttatatccgaactaattatggcattctcaagagagggcgcgcagaaggagtac
gtacaacataaaatgatggagaaggcggcgcaagtctgggacttaatcaaagaagaaggttatctttacgtctgcggagatgcaaa
aggaatggcaagagatgtgcacaggactttgcacacaatagttcaagaacaagaaggcgtcagttcctccgaagccgaagcgatc
gtgaaaaagttacagacagagggtaggtatttgcgtgatgtctgggggagcgccggtagtgcagcaggttccggggagtttatggtaa
gtaaaggtgaagaggacaatatggccattattaaagaatttatgcgtttcaaagtacacatggaagggtctgttaacggtcatgagtttg
agatagagggcgagggggaagggcgtccgtacgaaggaacacaaaccgcaaagctgaaagtaacgaaagggggggcctctac
catttgcttgggatattttgtccccgcaattcatgtatgggagcaaagcgtacgtcaagcatcccgccgacatacctgattacctaaaact
tagttttcctgaaggcttcaaatgggaaagagtgatgaatttcgaagacggcggcgtagtcacggtcacacaggatagttccttacagg
atggtgagttcatatacaaagtaaagttgcgtgggactaatttcccctctgatgggccagtaatgcaaaaaaagacgatggggtggga
ggcttctagtgaacgtatgtacccggaggacggggctctgaagggggagatcaaacagaggttaaaacttaaagacgggggtcact

acgacgccgaagttaaaactacctataaggctaagaagccagtgcaattacccggagcctataatgtgaacattaaattagacataa
catcacataacgaagattacactatagttgagcaatatgaaagagctgagggcagacatagtacgggaggaatggacgaactttac
aaatag

Abbreviated protein name:

*Vc*CYP94N1v2

Amino acid sequence:

MDLPSASAAVAAATAAVIFLLTIYLLPKKKSPASTGKNGSTSLESYPVIGNLPHFVKNRNRFLDWV
AEIISQSPTGTVIAAPLVFTSNPENVEHTAKSRFDAYARGPAATAVLHDFLGSGILNVDGDSWRA
QRKTASSEFTTRSLRAFILDAVDGEAAGRLLPLLSRAAASGEVFDLQDVLERFAFDNICSIIFDAD
PNCLNDTHDGVGERFYHAFHDATLLSTGRYYYPFHWVWRLLRWLNLGTEKRLRDAVSDVHKAI
DELVGSRKTEVGTTVRRQGGGSDLLSRFAEGGDYSDDVLRDVLINFVLAGRDTTPSALTWFFF
MISSRPDVVDQILDEIRSIRDHQDRSNPNGGGGGFTLEELREMNYLHAAITESLRLNPPVPLMP
KMCMEDDVLPDGTVVRRGWTVMYSAFAMGRKAEIWGEDCMEFKPERWLDDGGCFKSASAY
RLPAFHAGPRICLGKDMAYIQMKAVASSLLERFEVEVVEKRGKPELSITMRMDRGLPVRVKERK
RGC-

Native DNA sequence cloned directly from *V. californicum* for studies in *N. benthamiana*:[1]

atggatctaccctccgcctcagccgccgtcgccgccgctaccgctgccgtaatcttcctcctcactatctacctcctcccgaagaaaaa
atctccggcgtcgaccggaaagaacggatccacatccctgaagagctacccagtaatcggcaacctcccccacttcgtcaagaacc
gcaatcgcttcctcgactgggtggcggagataatatccctgagccccaccggcaccgtcatcgccgccccttggtcttcacctccaac
cccgagaacgtcgagcacaccgccaagtcccgcttcgacgcctacgcccgcggccccgccgccaccgccgtcctccacgacttcc
tcggcagcggcatcctcaacgtcgacggcgacggctggcgggcccagcggaagaccgccagctccgagttcaccacccgctccct
ccgcgccttcatcctcgacgccgtcgacggcgaggccgccggccggctcctcccccctcctctcccgcgccgctgcctccggcgaggt
gttcgacctccaggacgtcctcgagcggttcgccttcgacaacatctgcagcatcatcttcgacgccgaccccaactgcctcaacgac
acccacgacggcgtcggcgagcgcttctaccacgccttccacgacgccaccctgctctccaccgggaggtactactacccсttccact
gggtctggaggctcctgcgttggctcaacctcgggacagaaaagcggctgcgcgatgctgtgtcggacgttcacaaggcgatcgac
gaattggtcgggtcccggaagacggaggtcgggacgacggtgcggcggcagggcggcgggagcgatctcctctcgaggttcgcg
gagggcggggattactccgacgacgtcctccgcgacgtgctcatcaacttcgtcctcgccggccgcgacaccacccccctcggcgctc
acctggttctttttcatgatctcgtcgcggcccgacgtggtggacaagatcctcgacgagatccgatcgatccgagatcgtcaggaccgt
agcaacccgaacggcggaggcggtggtttcacgctggaggagctgagggagatgaattacctccacgcggcgataacggagtcg
ctccggctcaacccgccggtgccactgatgcctaagatgtgcatggaggacgacgtgctgccggacgggaccgtggtgcggcggg
ggtggacggtgatgtacagcgcgttcgcgatggggaggaaggcggaaatttgggggggaggattgcatggagttcaagccggagcg
ttggctggacgacggaggctgttttaagtcggcgagcgcgtatcggcttccggcgttccacgcgggtccgaggatttgcttggggaagg
acatggcctacatccagatgaaggcagtggcatcgagcttgctcgagaggttcgaggtcgaggtggtggagaagcgagggaagcc
cgagctgtcgatcacgatgaggatggatagggggctgccggtgagggtcaaggagaggaagagagggtgttag

[1]This gene for studies in *N. benthamiana* transient was clonded directly from *V. californicum* and is 99% identical to the published sequence for *Vc*CYP94Nv2.

Codon-optimized DNA sequence for studies in *S. cerevisiae*:

atggatcttccttcagcatcagcagcagtagcagcagcgactgccgcggtaattttcttactgactatatatttgttgccgaagaagaaga
gtcctgcttccaccggaaagaatggttctacttcccttgaatcctatccagtgatcggcaaccttccacacttcgtaaagaacagaaata
ggtttcttgactgggtggcagaaataattagccaaagccccactggtacagttatagccgcgccgttggttttcacgtcaaatcccgaaa
acgttgagcacaccgcgaagtctaggttcgatgcctatgcccgtggaccggccgcgaccgcagtgttacacgatttcttaggcagtgg
gatcttgaacgtagacggcgattcttggagagcacaaaggaagacagcttcatccgaattcactaccaggtctcttcgtgcgtttatattg
gacgcagtcgacggggaagcggcgggtagactgctgccccttttaagcagagcagctgcttcaggcgaagttttcgatcttcaggac
gtacttgaaaggttcgccttcgataacatctgttcaataatattcgacgcagatcccaactgccttaatgatacacacgacggggttggtg
agaggttttatcatgccttccatgacgctacacttctatccacggggcgttattactatcctttttcactgggtgtggagactgttgcgttggttg
aaccttggtacggaaaaaagattaagagacgctgtatccgatgttcacaaagcaattgacgaactggtaggctccagaaaaacaga
agtgggtactaccgtccgtcgtcaaggtggcggctctgatttgctaagcaggttcgcggaaggtggcgactacagcgacgacgttcta
agggatgtgctgatcaatttcgttttggctggtagagacacaacgcctagtgcgcttacttggtttttctttatgatttcttcaagacccgatgt
ggtagaccagatacttgatgagatcaggagcataagggaccaccaggatcgttccaatccaaatggtggcggaggaggcttcacct

tggaggagttacgtgaaatgaactacttgcacgccgctatcacagagagtttaaggctaaacccgcctgtgcccttaatgcccaaaat
gtgtatggaggacgacgttttgccagacggaactgtggtacgtcgtggctggacagtaatgtactctgcgtttgctatgggtagaaaggc
agagatctggggagaggactgtatggaatttaagcccgagagatggcttgatgatggtggttgttttaagtccgcctctgcttatcgtttac
cggcttttcatgccggtcctagaatttgtctaggcaaagatatggcgtatatccagatgaaagccgttgccagctccttgctggagcgtttt
gaggttgaggtcgtggaaaaaaggggtaaaccggaactgtccatcacgatgcgtatggacaggggacttccagtccgtgtaaaaga
aagaaagaggggatgctag

Abbreviated protein name:

*Vn*CYP94N2

Amino acid sequence:

MDLPSATSAAVVIILLTIYLLTKQKSPATTGNSGSTSLKSYPIIGNLPHFVKNRNRFLDWVAEIIALS
PTDTVIAAPLVFTANPDNVEHTAKSRFDTYSRGPAATAVLHDFLGSGILNVDGDGWRAQRKTAS
SEFTTRSLRAFILDAVDGEAAGRLLPLLSRAAASGEVLDLQDVLERFAFDNICSIIFDADPNCLND
THDGVGERFYHAFHDATLLSTGRYYYPFHWVWRLLRWLNLGNEKRLRNAVSDVHKAIDELVGS
RRTEVGTTVRRQGGGSDLLSRFAEGGDYSDDVLRDVLINFVLAGRDTTPSALTWFFFSISSRPD
VVEKILDEIRSIRERQDRNNPDGGGGGFTLEELREMNYLHAAITESLRLNPPVPLMPKMCMEDD
VLPDGTVVRRGWTVMYSAFVMGRKAEIWGEDCLEFKPERWLDDGGCFKSASAYRLPAFHAG
PRICLGKDMAYIQMKAVASSMLERFEVEVVEKRGKPELSITMRMDRGLPVRVKERKRG-

Codon-optimized DNA sequence for studies in *S. cerevisiae*:

atggatctgcctagcgccacgagtgcagctgttgtgataatattacttaccatttatttgttgacaaagcagaaaagtccagcgaccaca
ggtaacagcgggagcacgtccttgaagtcctacccgatcatagggaatctgccacatttcgtcaagaaccgtaatagatttctggattg
ggtcgctgaaatcatcgcattgagtcccaccgataccgtcatagcagctcctctagtttttacggcgaatccggacaatgtggaacatac
cgccaagagccgttttgacacgtattccagagggccagccgcgaccgcggtattacacgatttccttggcagcggaatacttaatgttg
acggcgacggatggagagcacagaggaagaccgcttcctctgagtttaccacaaggtcattgagggcctttattctggacgctgttga
cggcgaagcggctgggaggttgctgccccttttgtcaagagccgctgcgtcaggcgaggtacttgatttgcaggacgttttggagagat
tcgcttttgataacatctgttccataatttttgatgccgacccgaattgcctaaacgatacacacgatggagtaggggagcgtttttatcacg
ccttccacgacgccacccttttaagcaccggccgttactattatccatttcattgggtttggagattgcttaggtggttgaacttgggaaacg
aaaaaaggcttcgtaacgctgtctccgatgttcataaggccatagacgagttggtgggaagtaggagaactgaagtcggtactacagt
acgtaggcagggtggtggctctgaccttttatctagattcgcggagggtggagattactccgatgacgttctgcgtgacgtcttgataaatt
tcgtgctggcgggacgtgacaccaccccgtccgctctaacctggttcttctttagtatttcttctaggccggatgttgttgagaaaattttaga
tgaaataaggtctattcgtgagcgtcaggacaggaacaatccagatggcggaggtggaggttttactttggaggagttgagggaaatg
aactacttacacgcagcgatcacggaatccctaaggctgaaccctcccgtccccttaatgcccaaaatgtgcatggaggatgatgttct
accagatggaacagtagttagaagggggtggacagtaatgtacagcgcgttcgtcatgggtagaaaagcagaaatctggggcgag
gattgcctagagttcaagcctgaaagatggctggatgatggtgggtgctttaagtcagcttctgcataccgtcttccggcattccacgcgg
gaccaaggatatgtctgggcaaggacatggcatacatccaaatgaaagccgttgcatcctctatgttagaaaggttcgaggtagaagt
ggtagagaaaagaggtaagccagagttgagcattacgatgagaatggatcgtggcttacctgtcagagttaaagagaggaagaga
ggttaa

Abbreviated protein name:

*Vn*CYP94N2$_{1\text{-}30}$–*Vc*CYP94N1v2$_{31\text{-}514}$

Amino acid sequence:

MDLPSATSAAVVIILLTIYLLTKQKSPATTSPASTGKNGSTSLESYPVIGNLPHFVKNRNRFLDWVA
EIISQSPTGTVIAAPLVFTSNPENVEHTAKSRFDAYARGPAATAVLHDFLGSGILNVDGDSWRAQ
RKTASSEFTTRSLRAFILDAVDGEAAGRLLPLLSRAAASGEVFDLQDVLERFAFDNICSIIFDADP
NCLNDTHDGVGERFYHAFHDATLLSTGRYYYPFHWVWRLLRWLNLGTEKRLRDAVSDVHKAI
DELVGSRKTEVGTTVRRQGGGSDLLSRFAEGGDYSDDVLRDVLINFVLAGRDTTPSALTWFFF
MISSRPDVVDQILDEIRSIRDHQDRSNPNGGGGGFTLEELREMNYLHAAITESLRLNPPVPLMP
KMCMEDDVLPDGTVVRRGWTVMYSAFAMGRKAEIWGEDCMEFKPERWLDDGGCFKSASAY
RLPAFHAGPRICLGKDMAYIQMKAVASSLLERFEVEVVEKRGKPELSITMRMDRGLPVRVKERK
RGC-

Codon-optimized DNA sequence for studies in *S. cerevisiae*:

atggatctgcctagcgccacgagtgcagctgttgtgataatattacttaccatttatttgttgacaaagcagaaaagtccagcgaccaca
agtcctgcttccaccggaaagaatggttctacttcccttgaatcctatccagtgatcggcaaccttccacacttcgtaaagaacagaaat
aggtttcttgactgggtggcagaaataattagccaaagccccactggtacagttatagccgcgccgttggttttcacgtcaaatcccgaa
aacgttgagcacaccgcgaagtctaggttcgatgcctatgcccgtggaccggccgcgaccgcagtgttacacgatttcttaggcagtg
ggatcttgaacgtagacggcgattcttggagagcacaaaggaagacagcttcatccgaattcactaccaggtctcttcgtgcgtttatatt
ggacgcagtcgacggggaagcggcgggtagactgctgccccttttaagcagagcagctgcttcaggcgaagttttcgatcttcagga
cgtacttgaaaggttcgccttcgataacatctgttcaataatattcgacgcagatcccaactgccttaatgatacacacgacggggttggt
gagaggttttatcatgccttccatgacgctacacttctatccacggggcgttattactatcctttttcactgggtgtggagactgttgcgttggtt
gaaccttggtacggaaaaaagattaagagacgctgtatccgatgttcacaaagcaattgacgaactggtaggctccagaaaaacag
aagtgggtactaccgtccgtcgtcaaggtggcggctctgatttgctaagcaggttcgcggaaggtggcgactacagcgacgacgttct
aagggatgtgctgatcaatttcgttttggctggtagagacacaacgcctagtgcgcttacttggtttttctttatgatttcttcaagacccgatg
tggtagaccagatacttgatgagatcaggagcataagggaccaccaggatcgttccaatccaaatggtggcggaggaggcttcacc
ttggaggagttacgtgaaatgaactacttgcacgccgctatcacagagagtttaaggctaaacccgcctgtgcccttaatgcccaaaat
gtgtatggaggacgacgttttgccagacggaactgtggtacgtcgtggctggacagtaatgtactctgcgtttgctatgggtagaaaggc
agagatctggggagaggactgtatggaatttaagcccgagagatggcttgatgatggtggttgttttaagtccgcctctgcttatcgtttac
cggcttttcatgccggtcctagaatttgtctaggcaaagatatggcgtatatccagatgaaagccgttgccagctccttgctggagcgtttt
gaggttgaggtcgtggaaaaaaggggtaaaccggaactgtccatcacgatgcgtatggacaggggacttccagtccgtgtaaaaga
aagaaagaggggatgctag

Abbreviated protein name:

*Vc*CYP94N1v2–mCherry

Amino acid sequence:

MDLPSASAAVAAATAAVIFLLTIYLLPKKKSPASTGKNGSTSLESYPVIGNLPHFVKNRNRFLDWV
AEIISQSPTGTVIAAPLVFTSNPENVEHTAKSRFDAYARGPAATAVLHDFLGSGILNVDGDSWRA
QRKTASSEFTTRSLRAFILDAVDGEAAGRLLPLLSRAAASGEVFDLQDVLERFAFDNICSIIFDAD
PNCLNDTHDGVGERFYHAFHDATLLSTGRYYYPFHWVWRLLRWLNLGTEKRLRDAVSDVHKAI
DELVGSRKTEVGTTVRRQGGGSDLLSRFAEGGDYSDDVLRDVLINFVLAGRDTTPSALTWFFF
MISSRPDVVDQILDEIRSIRDHQDRSNPNGGGGGFTLEELREMNYLHAAITESLRLNPPVPLMP
KMCMEDDVLPDGTVVRRGWTVMYSAFAMGRKAEIWGEDCMEFKPERWLDDGGCFKSASAY
RLPAFHAGPRICLGKDMAYIQMKAVASSLLERFEVEVVEKRGKPELSITMRMDRGLPVRVKERK
RGCGSAGSAAGSGEFMVSKGEEDNMAIIKEFMRFKVHMEGSVNGHEFEIEGEGEGRPYEGTQ
TAKLKVTKGGPLPFAWDILSPQFMYGSKAYVKHPADIPDYLKLSFPEGFKWERVMNFEDGGVV
TVTQDSSLQDGEFIYKVKLRGTNFPSDGPVMQKKTMGWEASSERMYPEDGALKGEIKQRLKL
KDGGHYDAEVKTTYKAKKPVQLPGAYNVNIKLDITSHNEDYTIVEQYERAEGRHSTGGMDELYK
-

Codon-optimized DNA sequence for studies in *S. cerevisiae*:

atggatcttccttcagcatcagcagcagtagcagcagcgactgccgcggtaattttcttactgactatatatttgttgccgaagaagaaga
gtcctgcttccaccggaaagaatggttctacttcccttgaatcctatccagtgatcggcaaccttccacacttcgtaaagaacagaaata
ggtttcttgactgggtggcagaaataattagccaaagccccactggtacagttatagccgcgccgttggttttcacgtcaaatcccgaaa
acgttgagcacaccgcgaagtctaggttcgatgcctatgcccgtggaccggccgcgaccgcagtgttacacgatttcttaggcagtgg
gatcttgaacgtagacggcgattcttggagagcacaaaggaagacagcttcatccgaattcactaccaggtctcttcgtgcgtttatattg
gacgcagtcgacggggaagcggcgggtagactgctgccccttttaagcagagcagctgcttcaggcgaagttttcgatcttcaggac
gtacttgaaaggttcgccttcgataacatctgttcaataatattcgacgcagatcccaactgccttaatgatacacacgacggggttggtg
agaggttttatcatgccttccatgacgctacacttctatccacggggcgttattactatccttttcactgggtgtggagactgttgcgttggttg
aaccttggtacggaaaaaagattaagagacgctgtatccgatgttcacaaagcaattgacgaactggtaggctccagaaaaacaga
agtgggtactaccgtccgtcgtcaaggtggcggctctgatttgctaagcaggttcgcggaaggtggcgactacagcgacgacgttcta
agggatgtgctgatcaatttcgttttggctggtagagacacaacgcctagtgcgcttacttggtttttctttatgatttcttcaagacccgatgt
ggtagaccagatacttgatgagatcaggagcataagggaccaccaggatcgttccaatccaaatggtggcggaggaggcttcacct
tggaggagttacgtgaaatgaactacttgcacgccgctatcacagagagtttaaggctaaacccgcctgtgcccttaatgcccaaaat
gtgtatggaggacgacgttttgccagacggaactgtggtacgtcgtggctggacagtaatgtactctgcgtttgctatgggtagaaaggc
agagatctggggagaggactgtatggaatttaagcccgagagatggcttgatgatggtggttgttttaagtccgcctctgcttatcgtttac
cggcttttcatgccggtcctagaatttgtctaggcaaagatatggcgtatatccagatgaaagccgttgccagctccttgctggagcgtttt
gaggttgaggtcgtggaaaaaaggggtaaaccggaactgtccatcacgatgcgtatggacaggggacttccagtccgtgtaaaaga
aagaaagaggggatgcgggagcgccggtagtgcagcaggttccggggagtttatggtaagtaaaggtgaagaggacaatatggc
cattattaaagaatttatgcgtttcaaagtacacatggaagggtctgttaacggtcatgagtttgagatagagggcgagggggaagggc
gtccgtacgaaggaacacaaaccgcaaagctgaaagtaacgaaagggggcctctaccatttgcttgggatattttgtccccgcaatt
catgtatgggagcaaagcgtacgtcaagcatcccgccgacatacctgattacctaaaacttagttttcctgaaggcttcaaatgggaaa
gagtgatgaatttcgaagacggcggcgtagtcacggtcacacaggatagttccttacaggatggtgagttcatatacaaagtaaagtt
gcgtgggactaatttcccctctgatgggccagtaatgcaaaaaaagacgatggggtgggaggcttctagtgaacgtatgtacccgga
ggacggggctctgaagggggagatcaaacagaggttaaaacttaaagacgggggtcactacgacgccgaagttaaaactacctat
aaggctaagaagccagtgcaattacccggagcctataatgtgaacattaaattagacataacatcacataacgaagattacactata
gttgagcaatatgaaagagctgagggcagacatagtacgggaggaatggacgaactttacaaatag

Abbreviated protein name:

*Vn*CYP94N2–mCherry

Amino acid sequence:

MDLPSATSAAVVIILLTIYLLTKQKSPATTGNSGSTSLKSYPIIGNLPHFVKNRNRFLDWVAEIIALS
PTDTVIAAPLVFTANPDNVEHTAKSRFDTYSRGPAATAVLHDFLGSGILNVDGDGWRAQRKTAS
SEFTTRSLRAFILDAVDGEAAGRLLPLLSRAAASGEVLDLQDVLERFAFDNICSIIFDADPNCLND
THDGVGERFYHAFHDATLLSTGRYYYPFHWVWRLLRWLNLGNEKRLRNAVSDVHKAIDELVGS
RRTEVGTTVRRQGGGSDLLSRFAEGGDYSDDVLRDVLINFVLAGRDTTPSALTWFFFSISSRPD
VVEKILDEIRSIRERQDRNNPDGGGGGFTLEELREMNYLHAAITESLRLNPPVPLMPKMCMEDD
VLPDGTVVRRGWTVMYSAFVMGRKAEIWGEDCLEFKPERWLDDGGCFKSASAYRLPAFHAG
PRICLGKDMAYIQMKAVASSMLERFEVEVVEKRGKPELSITMRMDRGLPVRVKERKRGGSAGS
AAGSGEFMVSKGEEDNMAIIKEFMRFKVHMEGSVNGHEFEIEGEGEGRPYEGTQTAKLKVTKG
GPLPFAWDILSPQFMYGSKAYVKHPADIPDYLKLSFPEGFKWERVMNFEDGGVVTVTQDSSLQ
DGEFIYKVKLRGTNFPSDGPVMQKKTMGWEASSERMYPEDGALKGEIKQRLKLKDGGHYDAE
VKTTYKAKKPVQLPGAYNVNIKLDITSHNEDYTIVEQYERAEGRHSTGGMDELYK-

Codon-optimized DNA sequence for studies in *S. cerevisiae*:

atggatctgcctagcgccacgagtgcagctgttgtgataatattacttaccatttatttgttgacaaagcagaaaagtccagcgaccaca
ggtaacagcgggagcacgtccttgaagtcctacccgatcatagggaatctgccacatttcgtcaagaaccgtaatagatttctggattg
ggtcgctgaaatcatcgcattgagtcccaccgataccgtcatagcagctcctctagtttttacggcgaatccggacaatgtggaacatac
cgccaagagccgttttgacacgtattccagagggccagccgcgaccgcggtattacacgatttccttggcagcggaatacttaatgttg
acggcgacggatggagagcacagaggaagaccgcttcctctgagtttaccacaaggtcattgagggcctttattctggacgctgttga
cggcgaagcggctgggaggttgctgccccttttgtcaagagccgctgcgtcaggcgaggtacttgatttgcaggacgttttggagagat
tcgcttttgataacatctgttccataatttttgatgccgacccgaattgcctaaacgatacacacgatggagtaggggagcgtttttatcacg
ccttccacgacgccacccttttaagcaccggccgttactattatccatttcattgggtttggagattgcttaggtggttgaacttgggaaacg
aaaaaaggcttcgtaacgctgtctccgatgttcataaggccatagacgagttggtgggaagtaggagaactgaagtcggtactacagt
acgtaggcagggtggtggctctgaccttttatctagattcgcggagggtggagattactccgatgacgttctgcgtgacgtcttgataaatt
tcgtgctggcgggacgtgacaccaccccgtccgctctaacctggttcttctttagtatttcttctaggccggatgttgttgagaaaattttaga
tgaaataaggtctattcgtgagcgtcaggacaggaacaatccagatggcggaggtggaggttttactttggaggagttgagggaaatg
aactacttacacgcagcgatcacggaatccctaaggctgaaccctcccgtccccttaatgcccaaaatgtgcatggaggatgatgttct
accagatggaacagtagttagaagggggtggacagtaatgtacagcgcgttcgtcatgggtagaaaagcagaaatctggggcgag
gattgcctagagttcaagcctgaaagatggctggatgatggtgggtgctttaagtcagcttctgcataccgtcttccggcattccacgcgg
gaccaaggatatgtctgggcaaggacatggcatacatccaaatgaaagccgttgcatcctctatgttagaaaggttcgaggtagaagt
ggtagagaaaagaggtaagccagagttgagcattacgatgagaatggatcgtggcttacctgtcagagttaaagagaggaagaga
ggtgggagcgccggtagtgcagcaggttccggggagtttatggtaagtaaaggtgaagaggacaatatggccattattaaagaattta
tgcgtttcaaagtacacatggaagggtctgttaacggtcatgagtttgagatagagggcgagggggaagggcgtccgtacgaagga
acacaaaccgcaaagctgaaagtaacgaaagggggcctctaccatttgcttgggatattttgtccccgcaattcatgtatgggagca
aagcgtacgtcaagcatcccgccgacatacctgattacctaaaacttagttttcctgaaggcttcaaatgggaaagagtgatgaatttcg
aagacggcggcgtagtcacggtcacacaggatagttccttacaggatggtgagttcatatacaaagtaaagttgcgtgggactaatttc
ccctctgatgggccagtaatgcaaaaaaagacgatggggtgggaggcttctagtgaacgtatgtacccggaggacggggctctgaa
gggggagatcaaacagaggttaaaacttaaagacgggggtcactacgacgccgaagttaaaactacctataaggctaagaagcc
agtgcaattacccggagcctataatgtgaacattaaattagacataacatcacataacgaagattacactatagttgagcaatatgaaa
gagctgagggcagacatagtacgggaggaatggacgaactttacaaatag

Abbreviated protein name:

*Vn*CYP94N2$_{1\text{-}30}$–*Vc*CYP94N1v2$_{31\text{-}514}$–mCherry

Amino acid sequence:

MDLPSATSAAVVIILLTIYLLTKQKSPATTSPASTGKNGSTSLESYPVIGNLPHFVKNRNRFLDWVA
EIISQSPTGTVIAAPLVFTSNPENVEHTAKSRFDAYARGPAATAVLHDFLGSGILNVDGDSWRAQ
RKTASSEFTTRSLRAFILDAVDGEAAGRLLPLLSRAAASGEVFDLQDVLERFAFDNICSIIFDADP
NCLNDTHDGVGERFYHAFHDATLLSTGRYYYPFHWVWRLLRWLNLGTEKRLRDAVSDVHKAI
DELVGSRKTEVGTTVRRQGGGSDLLSRFAEGGDYSDDVLRDVLINFVLAGRDTTPSALTWFFF
MISSRPDVVDQILDEIRSIRDHQDRSNPNGGGGGFTLEELREMNYLHAAITESLRLNPPVPLMP
KMCMEDDVLPDGTVVRRGWTVMYSAFAMGRKAEIWGEDCMEFKPERWLDDGGCFKSASAY
RLPAFHAGPRICLGKDMAYIQMKAVASSLLERFEVEVVEKRGKPELSITMRMDRGLPVRVKERK
RGCGSAGSAAGSGEFMVSKGEEDNMAIIKEFMRFKVHMEGSVNGHEFEIEGEGEGRPYEGTQ
TAKLKVTKGGPLPFAWDILSPQFMYGSKAYVKHPADIPDYLKLSFPEGFKWERVMNFEDGGVV
TVTQDSSLQDGEFIYKVKLRGTNFPSDGPVMQKKTMGWEASSERMYPEDGALKGEIKQRLKL
KDGGHYDAEVKTTYKAKKPVQLPGAYNVNIKLDITSHNEDYTIVEQYERAEGRHSTGGMDELYK
-

Codon-optimized DNA sequence for studies in *S. cerevisiae*:

atggatctgcctagcgccacgagtgcagctgttgtgataatattacttaccatttatttgttgacaaagcagaaaagtccagcgaccaca
agtcctgcttccaccggaaagaatggttctacttcccttgaatcctatccagtgatcggcaaccttccacacttcgtaaagaacagaaat
aggtttcttgactgggtggcagaaataattagccaaagccccactggtacagttatagccgcgccgttggttttcacgtcaaatcccgaa
aacgttgagcacaccgcgaagtctaggttcgatgcctatgcccgtggaccggccgcgaccgcagtgttacacgatttcttaggcagtg
ggatcttgaacgtagacggcgattcttggagagcacaaaggaagacagcttcatccgaattcactaccaggtctcttcgtgcgtttatatt
ggacgcagtcgacggggaagcggcgggtagactgctgccccttttaagcagagcagctgcttcaggcgaagttttcgatcttcagga
cgtacttgaaaggttcgccttcgataacatctgttcaataatattcgacgcagatcccaactgccttaatgatacacacgacggggttggt
gagaggttttatcatgccttccatgacgctacacttctatccacggggcgttattactatccttttcactgggtgtggagactgttgcgttggtt
gaaccttggtacggaaaaaagattaagagacgctgtatccgatgttcacaaagcaattgacgaactggtaggctccagaaaaacag
aagtgggtactaccgtccgtcgtcaaggtggcggctctgatttgctaagcaggttcgcggaaggtggcgactacagcgacgacgttct
aagggatgtgctgatcaatttcgttttggctggtagagacacaacgcctagtgcgcttacttggtttttctttatgatttcttcaagacccgatg
tggtagaccagatacttgatgagatcaggagcataagggaccaccaggatcgttccaatccaaatggtggcggaggaggcttcacc
ttggaggagttacgtgaaatgaactacttgcacgccgctatcacagagagtttaaggctaaacccgcctgtgcccttaatgcccaaaat
gtgtatggaggacgacgttttgccagacggaactgtggtacgtcgtggctggacagtaatgtactctgcgtttgctatgggtagaaaggc
agagatctggggagaggactgtatggaatttaagcccgagagatggcttgatgatggtggttgttttaagtccgcctctgcttatcgtttac
cggcttttcatgccggtcctagaatttgtctaggcaaagatatggcgtatatccagatgaaagccgttgccagctccttgctggagcgtttt
gaggttgaggtcgtggaaaaaaggggtaaaccggaactgtccatcacgatgcgtatggacaggggacttccagtccgtgtaaaaga
aagaaagaggggatgcgggagcgccggtagtgcagcaggttccggggagtttatggtaagtaaaggtgaagaggacaatatggc
cattattaaagaatttatgcgtttcaaagtacacatggaagggtctgttaacggtcatgagtttgagatagagggcgagggggaagggc
gtccgtacgaaggaacacaaaccgcaaagctgaaagtaacgaaagggggcctctaccatttgcttgggatattttgtccccgcaatt
catgtatgggagcaaagcgtacgtcaagcatcccgccgacatacctgattacctaaaacttagttttcctgaaggcttcaaatgggaaa
gagtgatgaatttcgaagacggcggcgtagtcacggtcacacaggatagttccttacaggatggtgagttcatatacaaagtaaagtt
gcgtgggactaatttcccctctgatgggccagtaatgcaaaaaaagacgatggggtgggaggcttctagtgaacgtatgtacccgga
ggacggggctctgaagggggagatcaaacagaggttaaaacttaaagacgggggtcactacgacgccgaagttaaaactacctat
aaggctaagaagccagtgcaattacccggagcctataatgtgaacattaaattagacataacatcacataacgaagattacactata
gttgagcaatatgaaagagctgagggcagacatagtacgggaggaatggacgaactttacaaatag

Abbreviated protein name:

*Vc*GABAT1v2

Amino acid sequence:

MGSTEAPVSTPNPGVQTVAANGSSCLAGTKSVEAKGFKGHDMLAPFTPGWKSTSLEPVVIEK
SEGCYVYDNHGTKYLDTIAGLWCTSLGGNEPRLIEAATAQLNKLPFYHNFCNRVTIPSLDLAKEL
LDMFTAKKMGKVFFTNSGSEANDTQVKLVRYYFNALGKPNKKKFIAQTKSFHGTTVASASLSGL
MHLHYKFDLPIPNVLHSDCPHYWRYHLPGESEEEFSSRLADNLEKLILKEEPDTVAAFIAEPVM
GASGVFLPPETYFEKIQAVLKKYDILFIADEVVTAFARLGTMFGCDKYNIQPDLVSLAKALSSAYIP
IGAVIVSQEISEVINRQSTQIGTFAHGCTFSGHPVACAVALETLKIYKERNIVERVQAISKRFQDGI
KAFSDSPIIGEIRGTGLIIGVDFTKNKSPNDVFPYKWGVGGIFVSECAKRGMIVHEPIGDCTTLSP
ALIISEEEIEQIIQIFGEALKSTEKQVEQLQSQNTAT-

Native DNA sequence cloned directly from *V. californicum* for studies in *N. benthamiana*:[1]

atgggatccactgaggcgcctgtatcaactcccaaccccggcgtacagacagttgcggcgaacgggtcatcatgcttggcagggac
caagtccgttgaagtgaaagggtttaaggggcatgacatgctggcacctttcacccctggatggaagagcacctccttggagcctgttg
tcattgagaagtcggagggttgttatgtttacgacaatcatggaacgaaatatcttgatactattgctgggttgtggtgcacatctttaggag
gaaatgagcctcgacttatagaagctgcaactgcacagttaaataaattgccattttaccacaacttctgcaatcgtgtcaccatgccctc
tttggatctagccaaagaacttcttgatatgttcactgccaagaaaatgggaaaggtgttcttcactaatagtggttctgaggccaatgac
actcaggtgaagctggttcgatattatttcaatgcactgggaaagccaaataagaagaaatttatagctcaaacgaaatcgttccatgg
aactacagtagcatctgctagtctctcgggccttatgcatctacattataagttcgatctgccaattccaaatgtattgcactcagattgtcc
acactattggcgttaccatctaccaggtgagtcggaagaggagttctcgtcaaggttagctgataatttggagaaacttattctaaaaga
agaaccagatacggttgctgctttcattgctgaacctgtcatgggcgcatcgggtgttttccttcccccgagacctactttgagaagattc
aagctgtcctaaagaaatacgatattctcttcattgcggatgaggtcgtcactgcattcgcaaggttagggacaatgtttggatgtgacaa
atacaacattcagccggacctcgtctccttagcaaaagctctttcatctgcctacattcccatcggcgcagttatcgttagccaagaaattt
ctgaagttataaatcgtcaaagcacccaaattggtacatttgctcacgggtgtacgttttcggggcatccagtggcctgcgctgttgctttg
gagacacttaagatttataaggagaggaatattgttgagcgcgtccaggctatatcaaaaaggtttcaagatggcattaaagccttctct
gatagccccattattggggagattcgtggaaccgggttgatcatcggggtcgactttactaagaacaagtctccgaatgatatcttccctt
acaagtgggggggtcggaggaatatttgtatcagaatgcgcaaaacgcgggatgatagttcatgagcctatcggagattgcacgacgt
tgtcacctgcactgataatatctgaagaagagattgaacaaataatacaaatctttggagaagccctgaagagtacagagaagcaa
gtggagcagctccagtctcagaataccgcaacttaa

[1]This gene for studies in *N. benthamiana* transient was clonded directly from *V. californicum* and is 99% identical to the published sequence for *Vc*GABAT1v2.

Codon-optimized DNA sequence for studies in *S. cerevisiae*:

atggggtctacggaggccccggtgtccactccaaacccgggagtacagactgtggcggcgaacggatcttcctgcttggcggggac
gaagtcagttgaggcaaagggtttcaagggacatgacatgttagcccccttttacccccggttggaaatctacttctcttgaacccgtagt
catagagaagtctgaggggtgttatgtatacgataaccatggaacgaaatacctggacacgatagcaggtctgtggtgtactagccttg
gcggaaatgaacctagattgatcgaggcagcgaccgctcaattgaacaagttgccgttttaccacaatttttgcaacagagtgactatc
ccctccttagatcttgctaaagagctgttagacatgtttaccgcgaaaaagatggggaaagtattttttacaaacagcggttcagaagcg
aatgatacgcaggtgaaattagtaaggtactacttcaatgcacttggcaaaccgaataaaaaaaagttcatcgctcagactaagtcctt
ccatgggacaactgtcgcatcagccagtctatctgggcttatgcacctgcattacaagtttgatttaccgattcccaatgttttgcactcaga
ttgtccacactactggaggtaccatttacctggggaaagtgaagaagaattctcttcaaggttagctgataatcttgagaaactaattttga
aagaggaacccgacaccgttgctgcttttattgcggaaccggtaatgggtgcttccggtgtctttctaccgccggaaacgtattttgagaa
aatacaggctgtgctgaagaaatatgatatccttttcatcgctgatgaggtagtgacagcgtttgcgcgtctgggtaccatgttcggatgc
gataaatataatatccagccagaccttgtaagccttgctaaagccttgagtagtgcatatattcctatcggggcggtcatcgtcagtcaag
aaatatctgaggtgatcaacaggcaatcaacgcagattgggactttcgcgcacggttgtacgttctcagggcaccccgtcgcatgtgc
agtggccttggagaccttaaagatatacaaggagagaaatattgtagagagggtgcaggcaatatccaaaaggtttcaagatggtatt
aaagcatttagcgattcaccaataataggtgagatcagggggactggcctgataattggtgtggattttaccaaaaacaagtcccctaa

tgacgtgtttccttacaagtggggtgttggcggcattttcgtatctgaatgtgccaaaagaggaatgattgttcatgagccaataggggatt
gcaccactttaagtccggcattgataatatctgaggaagaaatagagcagatcatacagatcttcggagaagccttgaagtccactga
gaagcaggtcgaacagctgcagtcccagaatacggcgacttaa

Abbreviated protein name:

*Vc*GABAT1v2–mCherry

Amino acid sequence:

MGSTEAPVSTPNPGVQTVAANGSSCLAGTKSVEAKGFKGHDMLAPFTPGWKSTSLEPVVIEK
SEGCYVYDNHGTKYLDTIAGLWCTSLGGNEPRLIEAATAQLNKLPFYHNFCNRVTIPSLDLAKEL
LDMFTAKKMGKVFFTNSGSEANDTQVKLVRYYFNALGKPNKKKFIAQTKSFHGTTVASASLSGL
MHLHYKFDLPIPNVLHSDCPHYWRYHLPGESEEEFSSRLADNLEKLILKEEPDTVAAFIAEPVM
GASGVFLPPETYFEKIQAVLKKYDILFIADEVVTAFARLGTMFGCDKYNIQPDLVSLAKALSSAYIP
IGAVIVSQEISEVINRQSTQIGTFAHGCTFSGHPVACAVALETLKIYKERNIVERVQAISKRFQDGI
KAFSDSPIIGEIRGTGLIIGVDFTKNKSPNDVFPYKWGVGGIFVSECAKRGMIVHEPIGDCTTLSP
ALIISEEEIEQIIQIFGEALKSTEKQVEQLQSQNTATGSAGSAAGSGEFMVSKGEEDNMAIIKEFM
RFKVHMEGSVNGHEFEIEGEGEGRPYEGTQTAKLKVTKGGPLPFAWDILSPQFMYGSKAYVKH
PADIPDYLKLSFPEGFKWERVMNFEDGGVVTVTQDSSLQDGEFIYKVKLRGTNFPSDGPVMQK
KTMGWEASSERMYPEDGALKGEIKQRLKLKDGGHYDAEVKTTYKAKKPVQLPGAYNVNIKLDI
TSHNEDYTIVEQYERAEGRHSTGGMDELYK-

Codon-optimized DNA sequence for studies in *S. cerevisiae*:

atggggtctacggaggccccggtgtccactccaaacccgggagtacagactgtggcggcgaacggatcttcctgcttggcggggac
gaagtcagttgaggcaaagggtttcaagggacatgacatgttagcccctttacccccggttggaaatctacttctcttgaacccgtagt
catagagaagtctgaggggtgttatgtatacgataaccatggaacgaaatacctggacacgatagcaggtctgtggtgtactagccttg
gcggaaatgaacctagattgatcgaggcagcgaccgctcaattgaacaagttgccgttttaccacaattttgcaacagagtgactatc
ccctccttagatcttgctaaagagctgttagacatgtttaccgcgaaaaagatggggaaagtatttttacaaacagcggttcagaagcg
aatgatacgcaggtgaaattagtaaggtactacttcaatgcacttggcaaaccgaataaaaaaaagttcatcgctcagactaagtcctt
ccatgggacaactgtcgcatcagccagtctatctgggcttatgcacctgcattacaagtttgatttaccgattcccaatgttttgcactcaga
ttgtccacactactggaggtaccatttacctggggaaagtgaagaagaattctcttcaaggttagctgataatcttgagaaactaattttga
aagaggaacccgacaccgttgctgcttttattgcggaaccggtaatgggtgcttccggtgtctttctaccgccggaaacgtatttttgagaa
aatacaggctgtgctgaagaaatatgatatccttttcatcgctgatgaggtagtgacagcgtttgcgcgtctgggtaccatgttcggatgc
gataaatataatatccagccagaccttgtaagccttgctaaagccttgagtagtgcatatattcctatcggggcggtcatcgtcagtcaag
aaatatctgaggtgatcaacaggcaatcaacgcagattgggactttcgcgcacggttgtacgttctcagggcaccccgtcgcatgtgc
agtggccttggagaccttaaagatatacaaggagagaaatattgtagagagggtgcaggcaatatccaaaaggtttcaagatggtatt
aaagcatttagcgattcaccaataataggtgagatcaggggggactggcctgataattggtgtggattttaccaaaaacaagtcccctaa
tgacgtgtttccttacaagtggggtgttggcggcattttcgtatctgaatgtgccaaaagaggaatgattgttcatgagccaataggggatt
gcaccactttaagtccggcattgataatatctgaggaagaaatagagcagatcatacagatcttcggagaagccttgaagtccactga
gaagcaggtcgaacagctgcagtcccagaatacggcgactgggagcgccggtagtgcagcaggttccggggagtttatggtaagt
aaaggtgaagaggacaatatggccattattaaagaatttatgcgtttcaaagtacacatggaagggtctgttaacggtcatgagtttgag
atagagggcgaggggggaagggcgtccgtacgaaggaacacaaaccgcaaagctgaaagtaacgaaagggggggcctctaccat
ttgcttgggatattttgtccccgcaattcatgtatgggagcaaagcgtacgtcaagcatcccgccgacatacctgattacctaaaacttag
ttttcctgaaggcttcaaatgggaaagagtgatgaatttcgaagacggcggcgtagtcacggtcacacaggatagttccttacaggatg
gtgagttcatatacaaagtaaagttgcgtgggactaatttcccctctgatgggccagtaatgcaaaaaaagacgatggggtgggagg
cttctagtgaacgtatgtacccggaggacggggctctgaagggggagatcaaacagaggttaaaacttaaagacgggggtcactac
gacgccgaagttaaaactacctataaggctaagaagccagtgcaattacccggagcctataatgtgaacattaaattagacataaca
tcacataacgaagattacactatagttgagcaatatgaaagagctgagggcagacatagtacgggaggaatggacgaactttacaa
atag

Abbreviated protein name:

*Vc*CYP90G1v3

Amino acid sequence:

MTPLVVLFFLFPTLLVLVVAAFGQLAGKDDGWRKRGLRLPPGTMGWPLVGETLSFGKIHPSTSI
GDYLEEHIHKYGKIFKANLFASQAVVSVDAELNRFVMLNDGRLFEPCTPKGVLDILGHATPMALS
GDLHRYIKSLSVDFMGIGRMKSYFLPDAERYITETLASWEEGTPFQAKEEASKMMFNLMVKNV
LSMKAGVPETERLRKLYMSFMKGVIALPLNFPGSAYKKAVEARKVILGVINELMKERIQKRRDGT
DDIGEADLLGFVLEQSNLDAEQFGDLLLGLLFGGHETSATAITLLIYFLHDCPLAVKQLREEHMEI
VRMKRQRGEPAALTWEDYKLMEFSQCVVRETLRLGNVVKFIVRKASTDIKFKGYDIPKGWTVL
PILTAAHVDPSVYENVHKFDPWRWQTGSTSKALNDNYMPFGLGLRNCAGLQLAKLEIVVFLHH
LVLNFDWELAEPDNPIASPFPEFPRGLPIKVRRLSLLQ-

Native DNA sequence cloned directly from *V. californicum* for studies in *N. benthamiana*:[1]

atgactccattagttgttctcttctttctgttccctacactactagtgttggtggttgcagcatttgggcagctagcaggcaaggatgatgggt
ggaagaggagggggctgaggctcccaccgggcaccatgggttggccgctcgtcggagaaaccctatccttcggaaagatccactc
cagcacttccatcggagactacctcgaggagcgcatccacaagtacggaaagattttcaaggcgaacttgttcgcatctcaggcggt
ggtttcggtggacgcggagctgaaccggttcgtgatgctgaatgacgggcggctgttcgagccgtgcaccccgaaggggggtgctgga
catcctggggcacgcgacaccgatggcgttgtcgggtgatctgcaccgctacatcaagtctctgtccgttgatttcatggggatcggaa
ggatgaagagctacttcctccccgacgccgagcggtacatcacggagacgctcgcctcgtgggaggagggcacgccattccaagc
caaggaggaggcatccaagatgatgttcaatttgatggtgaagaacgttctcagcatgaaagctggtgtccccgagaccgagcggct
ccgcaagctttacatgtctttcatgaaggggggtcattgcgttacctctcaatttccctggatctgcctacaaaaaagccgtagaggcaag
aaaagtgattctgggagtgataaacgagttgatgaaggaaaggatccaaaagagaagagacggaacggacgatatcggcgaag
ctgacctattagggttcgtactcgagcagtccaacctcaacgccgagcagttcggcgatctcttgttgggtttgttgttcggcggccacga
gacctcagctacggccatcaccctgctcatctacttccttcacgactgccctttggccattcaacaactccgggaagagcacatggaga
tcgtgaggaggaaaaggcaaaaaagagagcctgctgcactgacatgggaggactacaaactaatggagtttagccaaggggggg
gtgcgagaaactcttcgattgggtaacgtggtcaagtttattgtgcgcaaggcgagcactgatattaaattcaaagggtatgatattccca
aagggtggaccgtgttgccgatcttgacagccgcccatgttgatccctctgtttacgagaacgttcacaaattcgatccatggagatggc
agaccggttctacaagcaaagccttgaacgacaactacatgcctttcggtttgggattgcgcaattgcgcgggcctgcaactcgccaa
gttggagattgttgtgtttcttcaccatctcgtgctcaacttcgactgggagctggcagagcccgacaatcccgtagcgtcccctttccccg
agtttcccaggggcctacccatcaaggttcgccggctttcgctcctccaataa

[1]This gene for studies in *N. benthamiana* transient was clonded directly from *V. californicum* and is 97% identical to the published sequence for *Vc*CYP90G1v3.

Codon-optimized DNA sequence for studies in *S. cerevisiae*:

atgaccccacttgtagtcctgttctttttatttccgaccttattggttctagtcgtcgctgcgttcggacaactggctggcaaggacgatggct
ggaggaagaggggcctaaggctacctcctggcacgatggggtggccattagtcggggagacgctgtcctttggcaaaatccaccct
agcacttctattggggattatctggaggaacacatccacaaatacggaaagatatttaaagcgaatttattcgcaagtcaagctgtagta
tccgtagatgctgaattgaaccgttttgtaatgttaaacgacggaagattattcgagccgtgcaccccctaaaggcgtactggacatattg
ggtcatgctactccaatggcgctatccggcgacctgcatcgttacataaagtccttatcagtagattttatggggatcggaaggatgaag
tcatactttttgcctgatgctgaacgttatattaccgaaactttggcttcatgggaagaaggtacaccttttcaggctaaggaagaagcaa
gtaaaatgatgtttaacctaatggtcaagaatgttcttagcatgaaggcgggtgtgcccgagaccgaaagacttagaaagttatatatgt
ctttcatgaaggggggttatagcacttccacttaactttcccgggagcgcatataaaaaggctgtagaggcccgtaaggtgatcttggga
gtcattaacgagctaatgaaagaaagaatccaaaagaggagggatgggacagacgatatcggcgaagctgacctactaggatttg
tgctggaacagtcaaatctggatgcggagcaattcggggatctattgttaggattactgttcgggggccatgagactagcgctacggct
attactttgctaatatatttccttcatgattgccccttggcggtaaagcaattaagggaagagcatatggaaatcgtgaggatgaagaga
caacgtggcgagcctgcggcattaacctgggaagactataaattgatggagttcagtcaatgtgtcgtacgtgagacactgcgtcttgg

caacgtggtgaagttcattgttaggaaggcgtcaacggacataaagtttaagggttatgacattcccaaagggtggacggttcttccaat
cctgaccgcagctcatgtcgacccttctgtgtatgagaatgtgcacaaattcgatccatggaggtggcaaactggctctaccagcaag
gcgctgaatgataactacatgcctttcgggttaggattaagaaattgcgccggattacaattggctaagctagagatagttgtttttctacat
cacctggtgttaaacttcgattgggaactggccgaaccagacaatcctatcgcttccccattccctgagtttcccagagggcttccaata
aaggttagaaggctatccctacttcaataa

Abbreviated protein name:

*Vc*CYP90G1v3–mCherry

Amino acid sequence:

MTPLVVLFFLFPTLLVLVVAAFGQLAGKDDGWRKRGLRLPPGTMGWPLVGETLSFGKIHPSTSI
GDYLEEHIHKYGKIFKANLFASQAVVSVDAELNRFVMLNDGRLFEPCTPKGVLDILGHATPMALS
GDLHRYIKSLSVDFMGIGRMKSYFLPDAERYITETLASWEEGTPFQAKEEASKMMFNLMVKNV
LSMKAGVPETERLRKLYMSFMKGVIALPLNFPGSAYKKAVEARKVILGVINELMKERIQKRRDGT
DDIGEADLLGFVLEQSNLDAEQFGDLLLGLLFGGHETSATAITLLIYFLHDCPLAVKQLREEHMEI
VRMKRQRGEPAALTWEDYKLMEFSQCVVRETLRLGNVVKFIVRKASTDIKFKGYDIPKGWTVL
PILTAAHVDPSVYENVHKFDPWRWQTGSTSKALNDNYMPFGLGLRNCAGLQLAKLEIVVFLHH
LVLNFDWELAEPDNPIASPFPEFPRGLPIKVRRLSLLQGSAGSAAGSGEFMVSKGEEDNMAIIK
EFMRFKVHMEGSVNGHEFEIEGEGEGRPYEGTQTAKLKVTKGGPLPFAWDILSPQFMYGSKAY
VKHPADIPDYLKLSFPEGFKWERVMNFEDGGVVTVTQDSSLQDGEFIYKVKLRGTNFPSDGPV
MQKKTMGWEASSERMYPEDGALKGEIKQRLKLKDGGHYDAEVKTTYKAKKPVQLPGAYNVNI
KLDITSHNEDYTIVEQYERAEGRHSTGGMDELYK-

Codon-optimized DNA sequence for studies in *S. cerevisiae*:

atgaccccacttgtagtcctgttctttttatttccgaccttattggttctagtcgtcgctgcgttcggacaactggctggcaaggacgatggct
ggaggaagaggggcctaaggctacctcctggcacgatggggtggccattagtcggggagacgctgtcctttggcaaaatccaccct
agcacttctattggggattatctggaggaacacatccacaaatacggaaagatatttaaagcgaatttattcgcaagtcaagctgtagta
tccgtagatgctgaattgaaccgttttgtaatgttaaacgacggaagattattcgagccgtgcaccccctaaaggcgtactggacatattg
ggtcatgctactccaatggcgctatccggcgacctgcatcgttacataaagtccttatcagtagattttatggggatcggaaggatgaag
tcatactttttgcctgatgctgaacgttatattaccgaaactttggcttcatgggaagaaggtacaccttttcaggctaaggaagaagcaa
gtaaaatgatgtttaacctaatggtcaagaatgttcttagcatgaaggcgggtgtgcccgagaccgaaagacttagaaagttatatatgt
ctttcatgaaggggggttatagcacttccacttaactttcccgggagcgcatataaaaaggctgtagaggcccgtaaggtgatcttggga
gtcattaacgagctaatgaaagaaagaatccaaaagaggagggatgggacagacgatatcggcgaagctgacctactaggatttg
tgctggaacagtcaaatctggatgcggagcaattcggggatctattgttaggattactgttcgggggccatgagactagcgctacggct
attactttgctaatatatttccttcatgattgccccttggcggtaaagcaattaagggaagagcatatggaaatcgtgaggatgaagaga
caacgtggcgagcctgcggcattaacctgggaagactataaattgatggagttcagtcaatgtgtcgtacgtgagacactgcgtcttgg
caacgtggtgaagttcattgttaggaaggcgtcaacggacataaagtttaagggttatgacattcccaaagggtggacggttcttccaat
cctgaccgcagctcatgtcgacccttctgtgtatgagaatgtgcacaaattcgatccatggaggtggcaaactggctctaccagcaag
gcgctgaatgataactacatgcctttcgggttaggattaagaaattgcgccggattacaattggctaagctagagatagttgtttttctacat
cacctggtgttaaacttcgattgggaactggccgaaccagacaatcctatcgcttccccattccctgagtttcccagagggcttccaata
aaggttagaaggctatccctacttcaagggagcgccggtagtgcagcaggttccggggagtttatggtaagtaaaggtgaagagga
caatatggccattattaaagaatttatgcgtttcaaagtacacatggaagggtctgttaacggtcatgagtttgagatagagggcgaggg
ggaagggcgtccgtacgaaggaacacaaaccgcaaagctgaaagtaacgaaagggggcctctaccatttgcttgggatattttgt
ccccgcaattcatgtatgggagcaaagcgtacgtcaagcatcccgccgacatacctgattacctaaaacttagttttcctgaaggcttca
aatgggaaagagtgatgaatttcgaagacggcggcgtagtcacggtcacacaggatagttccttacaggatggtgagttcatataca
aagtaaagttgcgtgggactaatttcccctctgatgggccagtaatgcaaaaaaagacgatggggtgggaggcttctagtgaacgtat
gtacccggaggacggggctctgaagggggagatcaaacagaggttaaaacttaaagacgggggtcactacgacgccgaagttaa
aactacctataaggctaagaagccagtgcaattacccggagcctataatgtgaacattaaattagacataacatcacataacgaaga
ttacactatagttgagcaatatgaaagagctgagggcagacatagtacgggaggaatggacgaactttacaaatag

Abbreviated protein name:

*Sv*MSBP

Amino acid sequence:

MEQKLISEEDLMSIEIWETLKEAITTYTGLSPTLFFTIVALSLAFYHAVFGLFGSSSSSSSSSSSNS
HQNPRNFVEESEPLPPPVQLGEITEDDLKNYDGSDSKKPLLMAIKGQIYDVSQSRMFYGPGGP
YALFAGKDASRALAKMSFEDKDLTGDISGLGPFELEALQDWEYKFMSKYVKVGTIKKDAPPSDA
PSPSEPSEAADVTDREAPKHAEDGPAETVEPSSVGDAEKKEE-

Codon-optimized DNA sequence for studies in *S. cerevisiae*:

atggaacagaagttgatttccgaagaagacctcatgtccattgagatttgggagacgttgaaggaagccatcacgacttacacaggat
tgagccccacactgttcttcactatcgtggctttgagtttagctttctaccatgccgtcttcggtttgtttgggagcagtagttctagctcttcatc
cagttcttccaatagtcaccagaacccacgtaacttcgtggaggagtcagagccactgccaccgcccgtgcagctaggcgagatca
ctgaggacgacctgaagaattatgacggcagcgactctaagaagcctctactaatggcgatcaaaggtcagatttatgacgtgtctca
gagcagaatgttctacggtccaggtgggccttacgcccttttttgcaggaaaggatgcttccagagctcttgctaaaatgagtttcgaaga
caaagatttgacgggggacatttccggtctaggacctttcgagttggaagccctgcaagactgggaatacaagttcatgtccaagtac
gtaaaagttggcactattaagaaagacgcccctccatccgacgccccaagcccttctgaaccctcagaggcagcagatgtaacaga
cagggaagcacccaagcacgctgaagacggacctgctgagacggtcgaaccgagctccgttggagacgctgaaaaaaaggaa
gagtaa

Supplementary Table S4. Gene-specific portion of DNA primers used to amplify genes for plasmid construction or *S. cerevisiae* strain construction. This table continues to page 56.

Gene Name	Primer Direction	Primer DNA Sequence
ERG1	Forward	ATGTCTGCTGTTAACGTTGCAC
	Reverse	TTAACCAATCAACTCACCAAACAAAAATG
ERG9	Forward	ATGGGAAAGCTATTACAATTGGCATTG
	Reverse	TCACGCTCTGTGTAAAGTGTATATATAATAAAAC
ERG20	Forward	ATGGCTTCAGAAAAAGAAATTAGGAGAG
	Reverse	CTATTTGCTTCTCTTGTAAACTTTGTTCAAG
ERG7	Forward	ATGACAGAATTTTATTCTGACACAATCG
	Reverse	TTAAAGCGTATGTGTTTCATATGCC
*Dr*DHCR7	Forward	ATGATGGCGTCAGACCGTGTC
	Reverse	TTAGAATATGTTCGGTAGTAGCCTATAGGAC
*Dr*DHCR24	Forward	ATGGATCCTTTGCTATACTTGGG
	Reverse	CTAATGACGCGCAGACTTACAG
*St*DHCR7	Forward	ATGGTGGAGAACAAGTTAGTCC
	Reverse	TTAGTAGATGCCCGGAATGAC
*Gg*DHCR24	Forward	ATGAGTGCGGTATGGTCCTTG
	Reverse	TTAATGTCTGGCCGCCTTG
*Vc*CYP90B27v1	Forward	ATGGCAATGGAACTATTACTGCTAA
	Reverse	TTAATCGCCCAAGGGCCTAAC
*Ec*CPR	Forward	ATGGAACAAACGGCTGTTAAAG
	Reverse	TTACCAGACATCACGTAAGTAACG
*Vc*CYP90B27v1–mCherry	Forward	ATGGCAATGGAACTATTACTGCTAA
	Reverse	CTATTTGTAAAGTTCGTCCATTCCTC
*Pp*CYP90B27	Forward	ATGGCTTTGGAGTTAATTTTGGTCT
	Reverse	TCATGCCTCACTTTTCTCAAGTG
*Ar*CYP71D443	Forward	ATGGAATTCACCTACTACTTTAGCC
	Reverse	TTAGGCCGGAAGAGTTCTTCTG

Gene Name	Primer Direction	Primer DNA Sequence
*At*CYP90B1	Forward	ATGTTCGAGACCGAGCATCATAC
	Reverse	TTATAATATCCTGGACACACGTATCGG
*Os*CYP90B2	Forward	ATGGCTGCGATGATGGC
	Reverse	TTACTCTTGTTCATCGTCTTGAGC
*Sl*CYP90B3	Forward	ATGTCAGATTTAGAATTCTTTTTGTTCTTAATTCC
	Reverse	TTAGTCATGAAGCTTGTTAAGGGAAC
*Tf*CYP90B51	Forward	ATGTCCGACTCTGACATAACCTTTT
	Reverse	TTAAATCAGACTGTGGCTTTGGAC
*Pp*CYP90B52	Forward	ATGGAAGGATTGCTACTGTTGC
	Reverse	TTAAGTAATGGCCCTGACTTTAATCTG
*Dz*CYP90B71	Forward	ATGGCACCTATGGAGCTGTTAC
	Reverse	TTATGCTGATGCTTTATGATCAATAGC
*Ia*CYP708A15v2	Forward	ATGAACCTAGTGTGGACAGCG
	Reverse	TTACTTGGGAAACGGGCTAAC
*At*CYP724A1	Forward	ATGGGCTGGCCGTTCATAG
	Reverse	TTAGTCTTCAAGGAATTTAGTTGGCTC
*Os*CYP724B1	Forward	ATGGTCGGAGGGGAGTTAG
	Reverse	TTAGGATTCACTGCAAAGAGGC
*Sl*CYP724B2	Forward	ATGGGAGAAGAGGGAAGCTTAC
	Reverse	TTAGGTTGAGTTTTTGTGTAACCTTATTG
*Dz*CYP90B71–mCherry	Forward	ATGGCACCTATGGAGCTGTTAC
	Reverse	CTATTTGTAAAGTTCGTCCATTCCTC
*Aa*CPR	Forward	ATGCAGTCAACTACATCAGTC
	Reverse	TCATTCCAAGTCCTCTTCGG
*At*CPR	Forward	ATGACGTCAGCTCTGTATGCC
	Reverse	TCACCAGACATCACGCAAATAC
*Ps*CPR	Forward	ATGGCACCAATCAATATCGAGG
	Reverse	TTATTGACGAAACGGCTTAGTCC

Gene Name	Primer Direction	Primer DNA Sequence
*Dz*CPR	Forward	ATGAAACTGAGTGCCCTGG
	Reverse	TTACCAGACATCACGTAGATACCTTC
*At*CPR–mCherry	Forward	ATGACGTCAGCTCTGTATGCC
	Reverse	CTATTTGTAAAGTTCGTCCATTCCTC
*Vc*CYP94N1v2	Forward	ATGGATCTTCCTTCAGCATCAG
	Reverse	CTAGCATCCCCTCTTTCTTTCTTTTAC
*Vn*CYP94N2	Forward	ATGGATCTGCCTAGCGC
	Reverse	TTAACCTCTCTTCCTCTCTTTAACTC
*Vn*CYP94N2$_{1\text{-}30}$–*Vc*CYP94N1v2$_{31\text{-}514}$	Forward	ATGGATCTGCCTAGCGC
	Reverse	CTAGCATCCCCTCTTTCTTTCTTTTAC
*Vc*CYP94N1v2–mCherry	Forward	ATGGATCTTCCTTCAGCATCAG
	Reverse	CTATTTGTAAAGTTCGTCCATTCCTCCC
*Vn*CYP94N2–mCherry	Forward	ATGGATCTGCCTAGCGC
	Reverse	CTATTTGTAAAGTTCGTCCATTCCTCCC
*Vn*CYP94N2$_{1\text{-}30}$–*Vc*CYP94N1v2$_{31\text{-}514}$–mCherry	Forward	ATGGATCTGCCTAGCGC
	Reverse	CTATTTGTAAAGTTCGTCCATTCCTCCC
*Vc*GABAT1v2	Forward	ATGGGGTCTACGGAGGC
	Reverse	TTAAGTCGCCGTATTCTGGG
*Vc*GABAT1v2–mCherry	Forward	ATGGGGTCTACGGAGGC
	Reverse	CTATTTGTAAAGTTCGTCCATTCCTCCC
*Vc*CYP90G1v3	Forward	ATGACCCCACTTGTAGTC
	Reverse	TTATTGAAGTAGGGATAGCCTTCTA
*Vc*CYP90G1v3–mCherry	Forward	ATGACCCCACTTGTAGTC
	Reverse	CTATTTGTAAAGTTCGTCCATTCCTC
*Sv*MSBP	Forward	ATGGAACAGAAGTTGATTTCCG
	Reverse	TTACTCTTCCTTTTTTTCAGCGTC

Supplementary Table S5. Gene-specific portion of DNA primers used to amplify *V. californicum* genes for transient expression in *N. benthamiana*. This table continues to page 58.

Gene Name	Primer Direction	Primer DNA Sequence
*Vc*CYP90B27v1	Forward	ATGGCGATGGAGCTCTTATTGT
	Reverse	TTAGTCTCCGAGGGGGCG
*Vc*CYP94N1v2	Forward	ATGGATCTACCCTCCGCCTC
	Reverse	CTAACACCCTCTCTTCCTCTCCTT
*Vc*GABAT1v2	Forward	ATGGGATCCACTGAGGCG
	Reverse	TTAAGTTGCGGTATTCTGAGACTGG
*Vc*CYP90G1v3	Forward	ATGACTCCATTAGTTGTTCTCTTCTTTCTG
	Reverse	TTATTGGAGGAGCGAAAGCCG
*Sl*SSR2	Forward	ATGTCGGATGCTAAGGCCCC
	Reverse	TCAATGTAGCTGCTTATTTAAAAGGCG
*Sl*3βHSD2	Forward	ATGGGTGAAGAAAAATGGTGTGTG
	Reverse	CTAATTCTTCTTGCTTTTGTCAAATATC
*Sl*SMO4	Forward	ATGGCTTCCATGATCGAATCTGCTTG
	Reverse	TTACATTGCTTTGCCCTCTTGC
*Sl*CPI	Forward	ATGAAAGGCAATAAAGTGAATAGTGC
	Reverse	TCAAGATGTCATGGCAAACCATG
*Sl*SDR	Forward	ATGGCAAATAAGCTCAGGTTGGAG
	Reverse	TTATTGTAGCTTCAAAATAGAACTTAG
*Sl*CYP51	Forward	ATGGAGTTAGGTGACAACAAGATTTTG
	Reverse	TTATTCAACAGAGAGCTTTCGGCG
*Sl*C14-R	Forward	ATGGATCTCAATAATCTCTTCATTTCTC
	Reverse	CTAGTAAACGTAAGGTAAGATCCTG
*Sl*8,7 SI	Forward	ATGGCAAGCCAGGGAGAAGC
	Reverse	TCAGCGGGTCTTGGTCTTCTGC
*Sl*SMO3	Forward	ATGTTGCCTTTTGAGAGTATTGAAG
	Reverse	TTATTCAGATTTGAAAACAGGTGCTG

Gene Name	Primer Direction	Primer DNA Sequence
SlC5	Forward	ATGGAGGATTACTTGAAGCTATTCG
	Reverse	TTACATTTTCTTGGCATCCTCTTCAAC
Sl7-DR2	Forward	ATGGCGGAGTCTCAGTTGGTTC
	Reverse	CTAATAAATTCCAGGGACTACCCGG

2.2. Guide of plasmids

Supplementary Table S6. Plasmids used in this study. This table continues to page 61.

**Plasmid Name**	**Genotype**	**Reference (JBEI ICE Registry Part ID)**
pCUT_KanMX	Cas9 protein and guide RNA to selectively cut in the KanMX marker	This study (JPUB_024453)
pCUT_YPRCΔ15	Cas9 protein and guide RNA to selectively cut at YPRCΔ15	[2] (JPUB_007481)
pCUT_ARS1021	Cas9 protein and guide RNA to selectively cut at ARS1021	[2] (JPUB_007472)
pCUT_ARS1014	Cas9 protein and guide RNA to selectively cut at ARS1014	[2] (JPUB_007473)
pCUT_ARS416	Cas9 protein and guide RNA to selectively cut at ARS416	[2] (JPUB_007463)
pCUT_ARS911	Cas9 protein and guide RNA to selectively cut at ARS911	[2] (JPUB_007471)
pCUT_ARS720	Cas9 protein and guide RNA to selectively cut at ARS720	[2] (JPUB_007469)
pCUT_ARS1309	Cas9 protein and guide RNA to selectively cut at ARS1309	[2] (JPUB_007476)
pCUT_ARS1622	Cas9 protein and guide RNA to selectively cut at ARS1622	[2] (JPUB_007480)
pCUT_ARS511	Cas9 protein and guide RNA to selectively cut at ARS511	[2] (JPUB_007464)
pESC-URA_PW-4	pGAL2-ERG1/ERG9-pGAL10/pGAL1-ERG20/ERG7-pGAL7	This study (JPUB_024455)
pESC-URA_PW-5	*Dr*DHCR7-pGAL10/pGAL1-*Dr*DHCR24	This study (JPUB_024457)
pESC-URA_PW-6	*St*DHCR7-pGAL10/pGAL1-*Dr*DHCR24	This study (JPUB_024459)
pESC-URA_PW-7	*Dr*DHCR7-pGAL10/pGAL1-*Gg*DHCR24	This study (JPUB_024461)
pESC-URA_PW-8	*St*DHCR7-pGAL10/pGAL1-*Gg*DHCR24	This study (JPUB_024463)
pESC-URA_PW-9	*Ec*CPR-pGAL10/pGAL1-*Vc*CYP90B27v1	This study (JPUB_024465)

Plasmid Name	Genotype	Reference (JBEI ICE Registry Part ID)
pESC-URA_PW-10	*Ec*CPR-pGAL10/pGAL1-*Vc*CYP90B27v1–mCherry	This study (JPUB_024467)
pESC-URA_PW-11	*Ec*CPR-pGAL10/pGAL1-*Pp*CYP90B27	This study (JPUB_024469)
pESC-URA_PW-12	*Ec*CPR-pGAL10/pGAL1-*Ar*CYP71D443	This study (JPUB_024471)
pESC-URA_PW-13	*Ec*CPR-pGAL10/pGAL1-*At*CYP90B1	This study (JPUB_024473)
pESC-URA_PW-14	*Ec*CPR-pGAL10/pGAL1-*Os*CYP90B2	This study (JPUB_024475)
pESC-URA_PW-15	*Ec*CPR-pGAL10/pGAL1-*Sl*CYP90B3	This study (JPUB_024477)
pESC-URA_PW-16	*Ec*CPR-pGAL10/pGAL1-*Tf*CYP90B51	This study (JPUB_024479)
pESC-URA_PW-17	*Ec*CPR-pGAL10/pGAL1-*Pp*CYP90B52	This study (JPUB_024481)
pESC-URA_PW-18	*Ec*CPR-pGAL10/pGAL1-*Dz*CYP90B71	This study (JPUB_024483)
pESC-URA_PW-19	*Ec*CPR-pGAL10/pGAL1-*Ia*CYP708A15v2	This study (JPUB_024485)
pESC-URA_PW-20	*Ec*CPR-pGAL10/pGAL1-*At*CYP724A1	This study (JPUB_024487)
pESC-URA_PW-21	*Ec*CPR-pGAL10/pGAL1-*Os*CYP724B1	This study (JPUB_024489)
pESC-URA_PW-22	*Ec*CPR-pGAL10/pGAL1-*Sl*CYP724B2	This study (JPUB_024491)
pESC-URA_PW-23	*Ec*CPR-pGAL10/pGAL1-*Dz*CYP90B71–mCherry	This study (JPUB_024493)
pESC-URA_PW-24	*Ec*CPR-pGal10/pGAL1-*Dz*CYP90B71	This study (JPUB_024495)
pESC-URA_PW-25	*Aa*CPR-pGal10/pGAL1-*Dz*CYP90B71	This study (JPUB_024497)
pESC-URA_PW-26	*At*CPR-pGal10/pGAL1-*Dz*CYP90B71	This study (JPUB_024499)

Plasmid Name	Genotype	Reference (JBEI ICE Registry Part ID)
pESC-URA_PW-27	*Ps*CPR-pGal10/pGAL1-*Dz*CYP90B71	This study (JPUB_024501)
pESC-URA_PW-28	*Dz*CPR-pGal10/pGAL1-*Dz*CYP90B71	This study (JPUB_024503)
pESC-URA_PW-29	mCherry–*At*CPR-pGal10/pGAL1-*Dz*CYP90B71	This study (JPUB_024505)
pESC-URA_PW-32	*At*CPR-pGAL10/pGAL1-*Vc*CYP94N1v2	This study (JPUB_024507)
pESC-URA_PW-33	*At*CPR-pGAL10/pGAL1-*Vn*CYP94N2	This study (JPUB_024509)
pESC-URA_PW-34	*At*CPR-pGAL10/pGAL1-*Vn*CYP94N2$_{1\text{-}30}$–*Vc*CYP94N1v2$_{31\text{-}514}$	This study (JPUB_024511)
pESC-URA_PW-35	*At*CPR-pGAL10/pGAL1-*Vc*CYP94N1v2–mCherry	This study (JPUB_024513)
pESC-URA_PW-36	*At*CPR-pGAL10/pGAL1-*Vn*CYP94N2–mCherry	This study (JPUB_024515)
pESC-URA_PW-37	*At*CPR-pGAL10/pGAL1-*Vn*CYP94N2$_{1\text{-}30}$–*Vc*CYP94N1v2$_{31\text{-}514}$–mCherry	This study (JPUB_024517)
pESC-URA_PW-38	empty-pGAL10/pGAL1-*Vc*GABAT1v2	This study (JPUB_024519)
pESC-URA_PW-39	*Vc*CYP90G1v3-pGAL10/pGAL1-*Vc*GABAT1v2–mCherry	This study (JPUB_024521)
pESC-URA_PW-40	*Vc*CYP90G1v3-pGAL10/pGAL1-*Vc*GABAT1v2	This study (JPUB_024523)
pESC-URA_PW-41	mCherry–*Vc*CYP90G1v3-pGAL10/pGAL1-*Vc*GABAT1v2	This study (JPUB_024525)
pESC-URA_PW-42	empty-pGAL10/pGAL1-*Sv*MSBP	This study (JPUB_024527)

2.3. Guide of *S. cerevisiae* strains

Supplementary Table S7. *S. cerevisiae* strains used in this study. This table continues to page 65.

Strain	Parent Strain	Genotype	Note	Reference (JBEI ICE Registry Part ID)
CEN.PK2-1C	n/a	MATa; his3D1; leu2-3_112; ura:3-52; trp1-289; MAL2-8c; SUC2	Wild type	[3]
GTy23	CEN.PK2-1C	erg9::KanMX_pCTR3-ERG9 leu2–3, 112::His3MX6_pGAL1-ERG19/pGAL10-ERG8 ura3–52::URA3_ pGAL1-mvaS(A110G)/pGAL10-mvaE(CO) his3Δ1::hphMX4_pGAL1-ERG12/pGAL10-IDI1	Upregulated mevalonate pathway	[4]
JWy601	GTy23	ura3–52 prototrophy removed	Enables the use of a URA3-based Cas9 gene integration system[2]	[4] (JPUB_010669)
PW-1	JWy601	erg9::pERG9-ERG9	Triterpenoid biosynthesis	This study (JPUB_024528)
PW-2	PW-1	erg5::pCTR3-ERG5	Enables repression of ergosterol biosynthesis	This study (JPUB_024530)
PW-3	PW-2	erg6::pCTR3-ERG6	Enables repression of ergosterol biosynthesis	This study (JPUB_024532)
PW-4	PW-3	YPRCΔ15::pGAL2-ERG1/ERG9-pGAL10/pGAL1-ERG20/ERG7-pGAL7	Cholesterol biosynthesis	This study (JPUB_024534)
PW-5	PW-4	ARS1021::*Dr*DHCR7-pGAL10/pGAL1-*Dr*DHCR24	22-Hydroxycholesterol biosynthesis	This study (JPUB_024536)
PW-6	PW-4	ARS1021::*St*DHCR7-pGAL10/pGAL1-*Dr*DHCR24	22-Hydroxycholesterol biosynthesis	This study (JPUB_024538)
PW-7	PW-4	ARS1021::*Dr*DHCR7-pGAL10/pGAL1-*Gg*DHCR24	22-Hydroxycholesterol biosynthesis	This study (JPUB_024540)

**Strain**	**Parent Strain**	**Genotype**	**Note**	**Reference (JBEI ICE Registry Part ID)**
PW-8	PW-4	ARS1021::*St*DHCR7-pGAL10/pGAL1-*Gg*DHCR24	22-Hydroxycholesterol biosynthesis	This study (JPUB_024542)
PW-9	PW-8	ARS1014::*Ec*CPR-pGAL10/pGAL1-*Vc*CYP90B27v1	22-Hydroxycholesterol biosynthesis	This study (JPUB_024544)
PW-10	PW-8	pESC_Ura3_*Ec*CPR-pGAL10/pGAL1-*Vc*CYP90B27v1–mCherry	Studying the Localization of *Vc*CYP90B27v1	This study (JPUB_024546)
PW-11	PW-8	ARS1014::*Ec*CPR-pGAL10/pGAL1-*Pp*CYP90B27	22-Hydroxycholesterol biosynthesis	This study (JPUB_024547)
PW-12	PW-8	ARS1014::*Ec*CPR-pGAL10/pGAL1-*Ar*CYP71D443	22-Hydroxycholesterol biosynthesis	This study (JPUB_024549)
PW-13	PW-8	ARS1014::*Ec*CPR-pGAL10/pGAL1-*At*CYP90B1	22-Hydroxycholesterol biosynthesis	This study (JPUB_024551)
PW-14	PW-8	ARS1014::*Ec*CPR-pGAL10/pGAL1-*Os*CYP90B2	22-Hydroxycholesterol biosynthesis	This study (JPUB_024553)
PW-15	PW-8	ARS1014::*Ec*CPR-pGAL10/pGAL1-*Sl*CYP90B3	22-Hydroxycholesterol biosynthesis	This study (JPUB_024555)
PW-16	PW-8	ARS1014::*Ec*CPR-pGAL10/pGAL1-*Tf*CYP90B51	22-Hydroxycholesterol biosynthesis	This study (JPUB_024557)
PW-17	PW-8	ARS1014::*Ec*CPR-pGAL10/pGAL1-*Pp*CYP90B52	22-Hydroxycholesterol biosynthesis	This study (JPUB_024559)
PW-18	PW-8	ARS1014::*Ec*CPR-pGAL10/pGAL1-*Dz*CYP90B71	22-Hydroxycholesterol biosynthesis	This study (JPUB_024561)
PW-19	PW-8	ARS1014::*Ec*CPR-pGAL10/pGAL1-*Ia*CYP708A15v2	22-Hydroxycholesterol biosynthesis	This study (JPUB_024563)

Strain	Parent Strain	Genotype	Note	Reference (JBEI ICE Registry Part ID)
PW-20	PW-8	ARS1014::*Ec*CPR-pGAL10/pGAL1-*At*CYP724A1	22-Hydroxycholesterol biosynthesis	This study (JPUB_024565)
PW-21	PW-8	ARS1014::*Ec*CPR-pGAL10/pGAL1-*Os*CYP724B1	22-Hydroxycholesterol biosynthesis	This study (JPUB_024567)
PW-22	PW-8	ARS1014::*Ec*CPR-pGAL10/pGAL1-*Sl*CYP724B2	22-Hydroxycholesterol biosynthesis	This study (JPUB_024569)
PW-23	PW-8	pESC_Ura3_*Ec*CPR-pGAL10/pGAL1-*Dz*CYP90B71–mCherry	Studying the Localization of *Dz*CYP90B71	This study (JPUB_024571)
PW-24	PW-8	pESC_Ura3_*Ec*CPR-pGal10/pGAL1-*Dz*CYP90B71	22-Hydroxycholesterol biosynthesis	This study (JPUB_024572)
PW-25	PW-8	pESC_Ura3_*Aa*CPR-pGal10/pGAL1-*Dz*CYP90B71	22-Hydroxycholesterol biosynthesis	This study (JPUB_024573)
PW-26	PW-8	pESC_Ura3_*At*CPR-pGal10/pGAL1-*Dz*CYP90B71	22-Hydroxycholesterol biosynthesis	This study (JPUB_024574)
PW-27	PW-8	pESC_Ura3_*Ps*CPR-pGal10/pGAL1-*Dz*CYP90B71	22-Hydroxycholesterol biosynthesis	This study (JPUB_024575)
PW-28	PW-8	pESC_Ura3_*Dz*CPR-pGal10/pGAL1-*Dz*CYP90B71	22-Hydroxycholesterol biosynthesis	This study (JPUB_024576)
PW-29	PW-18	pESC_Ura3_mCherry–*At*CPR-pGAL10/pGAL1-*Dz*CYP90B71	Studying the Localization of *At*CPR	This study (JPUB_024577)
PW-30	PW-8	ARS1014::*At*CPR-pGAL10/pGAL1-*Dz*CYP90B71	22-Hydroxycholesterol biosynthesis	This study (JPUB_024578)
PW-31	PW-30	ARS416::*At*CPR-pGAL10/pGAL1-*Dz*CYP90B71	22-Hydroxycholesterol biosynthesis	This study (JPUB_024580)

Strain	Parent Strain	Genotype	Note	Reference (JBEI ICE Registry Part ID)
PW-32	PW-31	ARS911::*At*CPR-pGAL10/pGAL1-*Vc*CYP94N1v2	22-Hydroxycholesterol-26-al biosynthesis	This study (JPUB_024582)
PW-33	PW-31	ARS911::*At*CPR-pGAL10/pGAL1-*Vn*CYP94N2	22-Hydroxycholesterol-26-al biosynthesis	This study (JPUB_024584)
PW-34	PW-31	ARS911::empty-pGAL10/pGAL1-*Vn*CYP94N2$_{1-30}$-*Vc*CYP94N1v2$_{31-514}$	22-Hydroxy-26-aminocholesterol biosynthesis	This study (JPUB_024586)
PW-35	PW-31	pESC_Ura3_*At*CPR-pGAL10/pGAL1-*Vc*CYP94N1v2–mCherry	Studying the Localization of *Vc*CYP94N1v2	This study (JPUB_024588)
PW-36	PW-31	pESC_Ura3_*At*CPR-pGAL10/pGAL1-*Vn*CYP94N2–mCherry	Studying the Localization of *Vn*CYP94N2	This study (JPUB_024589)
PW-37	PW-31	pESC_Ura3_*At*CPR-pGAL10/pGAL1-*Vn*CYP94N2$_{1-30}$–*Vc*CYP94N1v2$_{31-514}$–mCherry	Studying the Localization of *Vn*CYP94N2$_{1-30}$-*Vc*CYP94N1v2$_{31-514}$	This study (JPUB_024590)
PW-38	PW-33	ARS720::empty-pGAL10/pGAL1-*Vc*GABAT1v2	22-Hydroxy-26-aminocholesterol biosynthesis	This study (JPUB_024591)
PW-39	PW-18	pESC_Ura3_*Vc*CYP90G1v3-pGAL10/pGAL1-*Vc*GABAT1v2–mCherry	Studying the Localization of *Vc*GABAT1v2	This study (JPUB_024593)
PW-40	PW-33	ARS1309::*Vc*CYP90G1v3-pGAL10/pGAL1-*Vc*GABAT1v2	Verazine biosynthesis	This study (JPUB_024594)
PW-41	PW-18	pESC_Ura3_mCherry–*Vc*CYP90G1v3-pGAL10/pGAL1-*Vc*GABAT1v2	Studying the Localization of *Vc*CYP90G1v3	This study (JPUB_024596)
PW-42	PW-40	ARS511::empty-pGAL10/pGAL1-*Sv*MSBP	Verazine biosynthesis	This study (JPUB_024597)

Supplementary Table S8. Mean (n = 3) final optical density at 600 nm (OD_{600}) for the primary *S. cerevisiae* strains used in production experiments in this study after a 48 h growth stage and a 48 h production stage.

Strain	Final OD_{600}
PW-4	25.7 ± 0.9
PW-5	23.8 ± 0.9
PW-6	23.1 ± 2.4
PW-7	23.5 ± 0.6
PW-8	26.6 ± 0.4
PW-30	24.0 ± 0.8
PW-31	23.2 ± 1.6
PW-32	23.7 ± 0.3
PW-33	21.6 ± 1.5
PW-34	24.0 ± 0.7
PW-38	23.3 ± 0.6
PW-40	25.9 ± 0.6
PW-42	26.1 ± 0.3

2.4. Guide of *N. benthamiana* transient expression systems

Supplementary Table S9. *N. benthamiana* transient expression systems used in this study. Genes for *Vc*CYP90B27v1, *Vc*CYP94N1v2, *Vc*GABAT1v2, *Vc*CYP90G1v3 were cloned directly from *V. californicum* and were 97–99% identical to published sequences.

System Name	Transiently Expressed Genes	Note
JR-1	*Vc*CYP90B27v1, *Vc*CYP94N1v2, *Sl*SSR2, *Sl*3βHSD2, *Sl*SMO4, *Sl*CPI, *Sl*SDR, *Sl*CYP51, *Sl*C14-R, *Sl*8,7 SI, *Sl*SMO3, *Sl*C5, and *Sl*7-DR2	Produces 22-hydroxycholesterol-26-al (**8**)
JR-2	*Vc*CYP90B27v1, *Vc*CYP94N1v2, *Vc*GABAT1v2, *Sl*SSR2, *Sl*3βHSD2, *Sl*SMO4, *Sl*CPI, *Sl*SDR, *Sl*CYP51, *Sl*C14-R, *Sl*8,7 SI, *Sl*SMO3, *Sl*C5, and *Sl*7-DR2	Produces 22-hydroxy-26-aminocholesterol (**10**)
JR-3	*Vc*CYP90B27v1, *Vc*CYP94N1v2, *Vc*GABAT1v2, *Vc*CYP90G1v3, *Sl*SSR2, *Sl*3βHSD2, *Sl*SMO4, *Sl*CPI, *Sl*SDR, *Sl*CYP51, *Sl*C14-R, *Sl*8,7 SI, *Sl*SMO3, *Sl*C5, and *Sl*7-DR2	Produces verazine (**1**)

3. SUPPLEMENTARY DATA

3.1. Native verazine biosynthetic pathways in *V. californicum and V. nigrum*

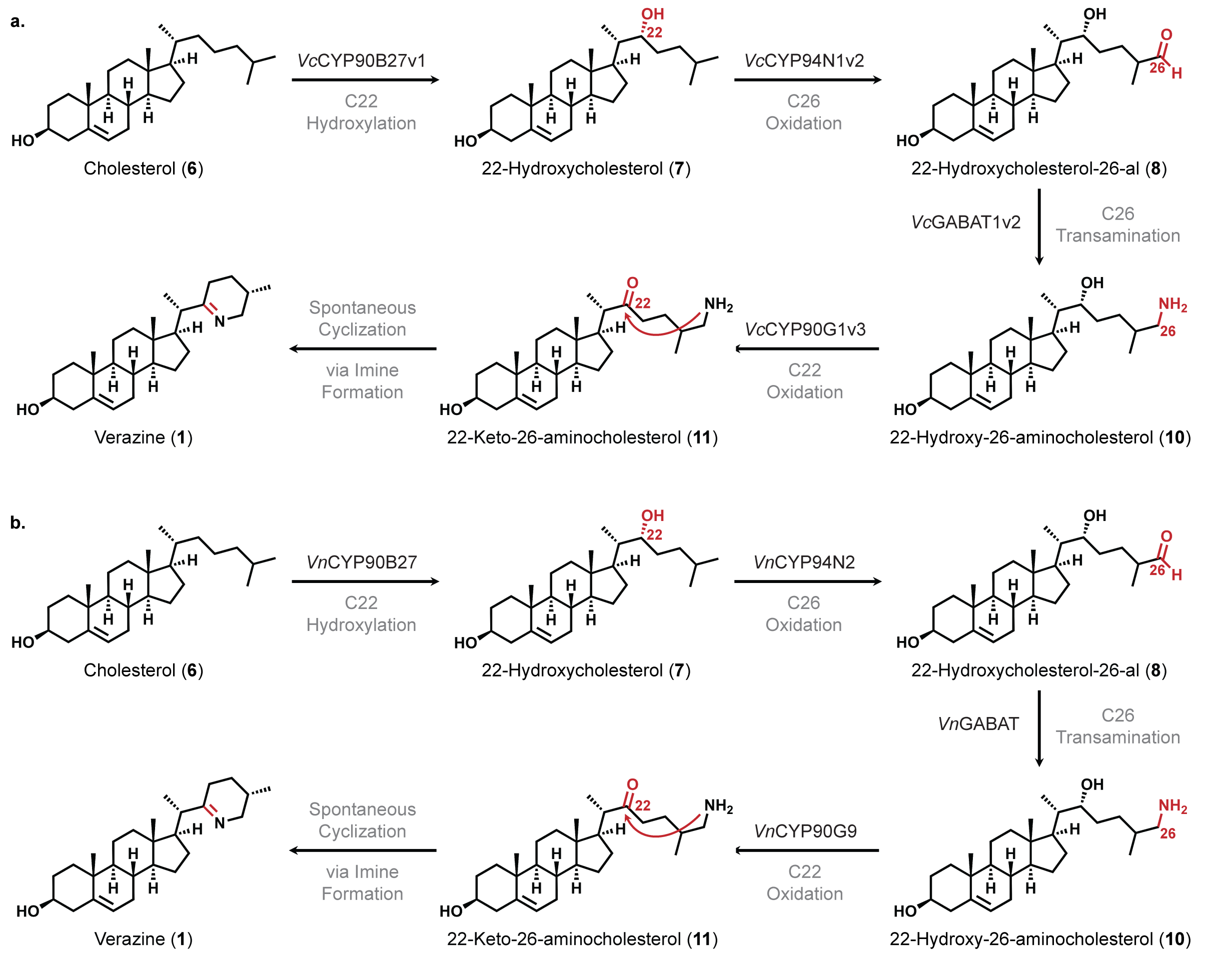

Supplementary Figure S1. The native biosynthetic pathway from cholesterol (**6**) to verazine (**1**) in **a.** *V. californicum*[5] and **b.** *V. nigrum*.[6]

3.2. Native mevalonate, triterpene, and sterol biosynthetic pathways in *S. cerevisiae*

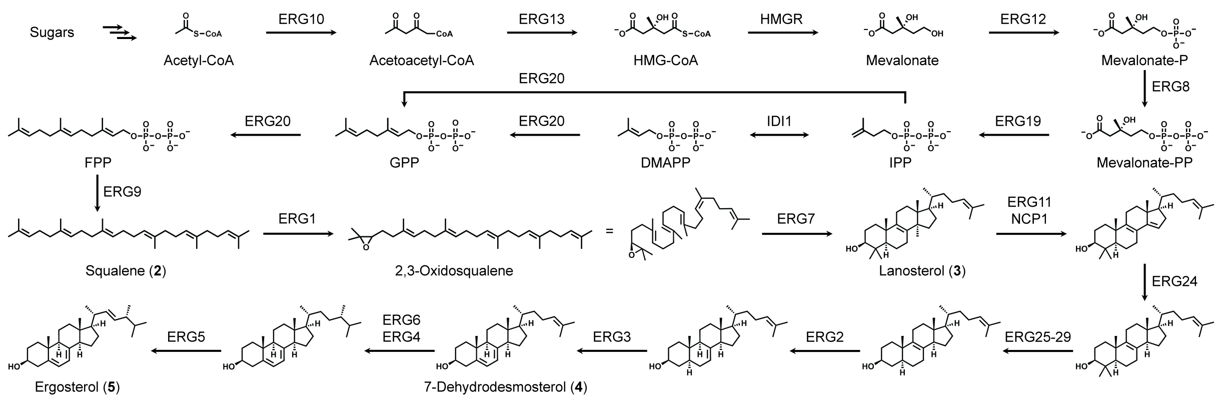

Supplementary Figure S2. The native biosynthetic pathway from sugars to ergosterol (**5**) in *S. cerevisiae*.[7] The final four steps in this pathway have an interchangeable order.[8]

3.3. Gene mining for *V. nigrum* enzymes

a.

```
VcCYP94N1v2 (AJT59561.1)      MDLPSASAAVAAATAAVIFLLTIYLLPKKKSPASTGKNGSTSLESYPVIGNLPHFVKNRN    60
VcCYP94N2v2 (AJT59563.1)      MDLPSATS---VAAAALIILLTIYLLTKQKSPATTGNSGSTSLKSYPIIGNLPHFVKNRN    57
VnCYP94N2 (WRU85179.1)        MDLPSATS-----AAVVIILLTIYLLTKQKSPATTGNSGSTSLKSYPIIGNLPHFVKNRN    54
                              ******::     :*.:*:******* *:****:**:.*****:***:************

VcCYP94N1v2 (AJT59561.1)      RFLDWVAEIISQSPTGTVIAAPLVFTSNPENVEHTAKSRFDAYARGPAATAVLHDFLGSG    120
VcCYP94N2v2 (AJT59563.1)      RFLDWVAEIISLSPTDTVIATPLVFTANPDNVEHTAKSRFDTYSRGPAATAVLHDFLGSG    117
VnCYP94N2 (WRU85179.1)        RFLDWVAEIIALSPTDTVIAAPLVFTANPDNVEHTAKSRFDTYSRGPAATAVLHDFLGSG    114
                              **********: ***.****:*****:**:***********:*:****************

VcCYP94N1v2 (AJT59561.1)      ILNVDGDSWRAQRKTASSEFTTRSLRAFILDAVDGEAAGRLLPLLSRAAASGEVFDLQDV    180
VcCYP94N2v2 (AJT59563.1)      ILNVDGESWRAQRKTASSEFTTRSLRAFILDAVDGEAAGRLLPLLSRAAASGEVLDLQDV    177
VnCYP94N2 (WRU85179.1)        ILNVDGDGWRAQRKTASSEFTTRSLRAFILDAVDGEAAGRLLPLLSRAAASGEVLDLQDV    174
                              ******:.**********************************************:*****

VcCYP94N1v2 (AJT59561.1)      LERFAFDNICSIIFDADPNCLNDTHDGVGERFYHAFHDATLLSTGRYYYPFHWVWRLLRW    240
VcCYP94N2v2 (AJT59563.1)      LERFAFDNICSIIFDADPNCLNDTHDGVGERFYHAFHDATLLSTGRYYYPFHWVWRLLRW    237
VnCYP94N2 (WRU85179.1)        LERFAFDNICSIIFDADPNCLNDTHDGVGERFYHAFHDATLLSTGRYYYPFHWVWRLLRW    234
                              ************************************************************

VcCYP94N1v2 (AJT59561.1)      LNLGTEKRLRDAVSDVHKAIDELVGSRKTEVGTTVRRQGGGSDLLSRFAEGGDYSDDVLR    300
VcCYP94N2v2 (AJT59563.1)      LNLGNEKRLRDAVSDVHKAIDELVGSRRTEVGTTVRRQGGGSDLLSRFAESGDYSDDVLR    297
VnCYP94N2 (WRU85179.1)        LNLGNEKRLRNAVSDVHKAIDELVGSRRTEVGTTVRRQGGGSDLLSRFAEGGDYSDDVLR    294
                              ****.*****:****************:**********************.*********

VcCYP94N1v2 (AJT59561.1)      DVLINFVLAGRDTTPSALTWFFFMISSRPDVVDQILDEIRSIRDHQDRSNPNGGGGGFTL    360
VcCYP94N2v2 (AJT59563.1)      DVLINFVLAGRDTTPSALTWFFFSISLRPDVVDKILDEIRSIRDRQDRNNPNSGGGGFTL    357
VnCYP94N2 (WRU85179.1)        DVLINFVLAGRDTTPSALTWFFFSISSRPDVVEKILDEIRSIRERQDRNNPDGGGGGFTL    354
                              *********************** ** *****::*********::***.**:.*******

VcCYP94N1v2 (AJT59561.1)      EELREMNYLHAAITESLRLNPPVPLMPKMCMEDDVLPDGTVVRRGWTVMYSAFAMGRKAE    420
VcCYP94N2v2 (AJT59563.1)      EELREMNYLHAAITESLRLNPPVPLMPKMCMEDDVLPDGTVVRRGWTVMYSAFVMGRKAE    417
VnCYP94N2 (WRU85179.1)        EELREMNYLHAAITESLRLNPPVPLMPKMCMEDDVLPDGTVVRRGWTVMYSAFVMGRKAE    414
                              *****************************************************.******

VcCYP94N1v2 (AJT59561.1)      IWGEDCMEFKPERWLDDGGCFKSASAYRLPAFHAGPRICLGKDMAYIQMKAVASSLLERF    480
VcCYP94N2v2 (AJT59563.1)      IWGEDCMEFKPERWLDDGGCFKSASAYRLPAFHAGPRICLGKDMAYIQMKAVASSMLERF    477
VnCYP94N2 (WRU85179.1)        IWGEDCLEFKPERWLDDGGCFKSASAYRLPAFHAGPRICLGKDMAYIQMKAVASSMLERF    474
                              ******:************************************************:****

VcCYP94N1v2 (AJT59561.1)      EVEVVEKRGKPELSITMRMDRGLPVRVKERKRGC-  514
VcCYP94N2v2 (AJT59563.1)      EVEVVEKRGKPELSITMRMDRGLPVRIKERKRGC-  511
VnCYP94N2 (WRU85179.1)        EVEVVEKRGKPELSITMRMDRGLPVRVKERKRGF-  508
                              **************************:****** *
```

b.

	***Vc*CYP94N1v2**	***Vc*CYP94N2v2**	***Vn*CYP94N2**
***Vc*CYP94N1v2**	100% (100%)		
***Vc*CYP94N2v2**	93.4% (95.1%)	100% (100%)	
***Vn*CYP94N2**	93.1% (94.7%)	96.9% (97.8%)	100% (100%)

Supplementary Figure S3. Multiple sequence alignment of CYP94N P450s involved in verazine biosynthesis. **a.** Multiple sequence alignment of CYP94N P450s from *V. californicum* and *V. nigrum*. Predicted transmembrane segments are shown in red. **b.** Percent identity and similarity matrix of aligned CYP94N P450s. Similarity is given in parentheses.

3.4. GC-MS characterization

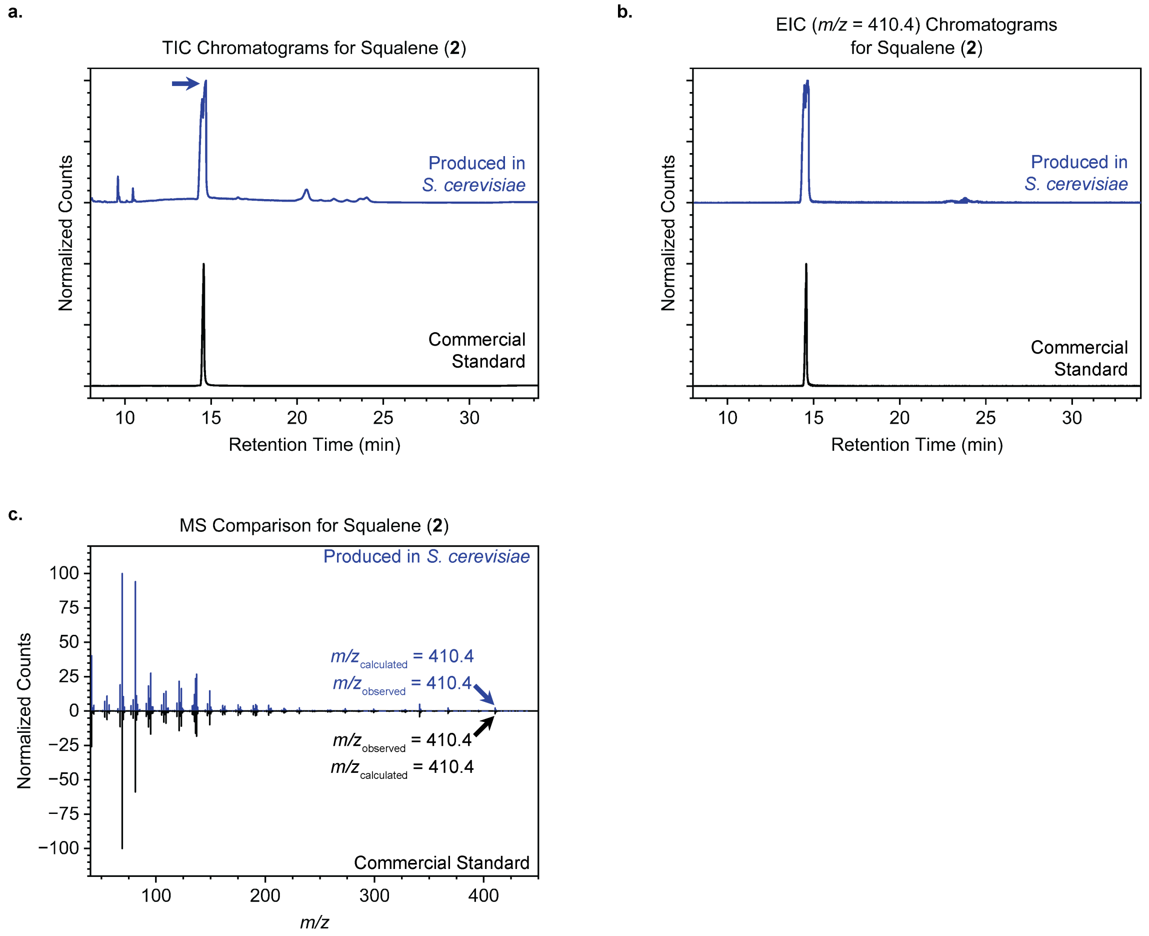

Supplementary Figure S4. a. Total ion chromatograms (TIC), **b.** extracted ion chromatograms (EIC, *m/z* = 410.4), and **c.** mass spectra from GC-MS characterization of squalene (**2**) produced in *S. cerevisiae* strain PW-4 (top, blue) and squalene (**2**) from a commercial standard (bottom, black).

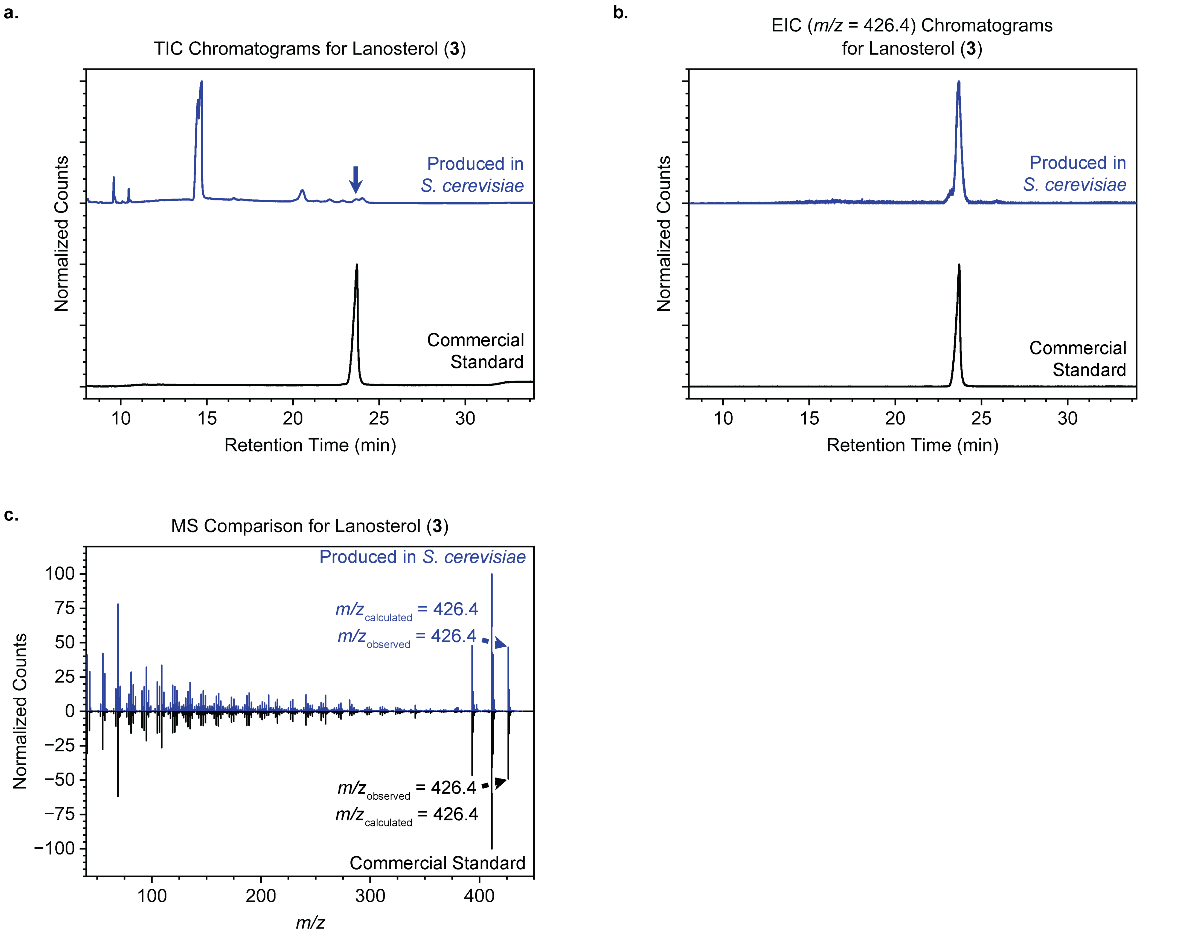

Supplementary Figure S5. a. Total ion chromatograms (TIC), **b.** extracted ion chromatograms (EIC, *m/z* = 426.4), and **c.** mass spectra from GC-MS characterization of lanosterol (**3**) produced in *S. cerevisiae* strain PW-4 (top, blue) and lanosterol (**3**) from a commercial standard (bottom, black).

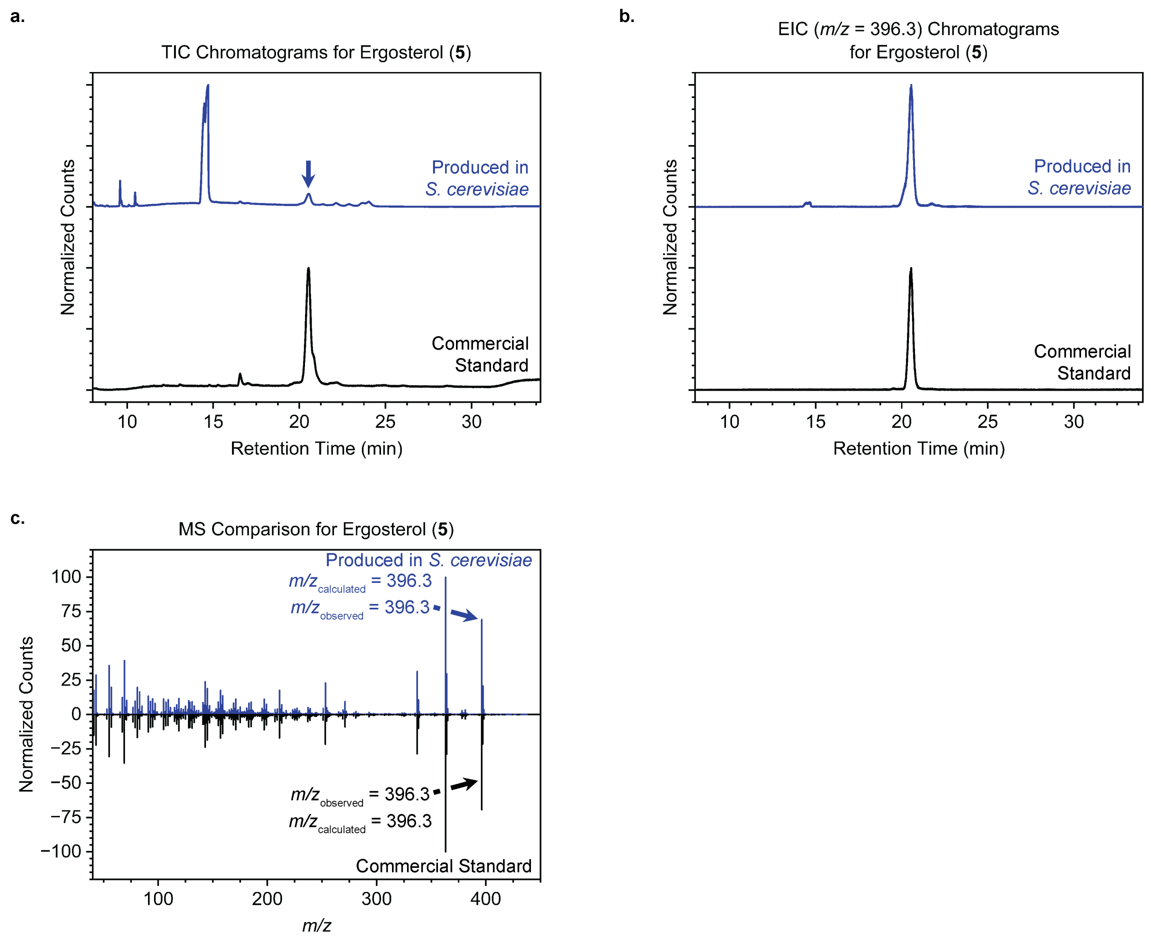

Supplementary Figure S6. a. Total ion chromatograms (TIC), **b.** extracted ion chromatograms (EIC, *m/z* = 396.3), and **c.** mass spectra from GC-MS characterization of ergosterol (**5**) produced in *S. cerevisiae* strain PW-4 (top, blue) and ergosterol (**5**) from a commercial standard (bottom, black).

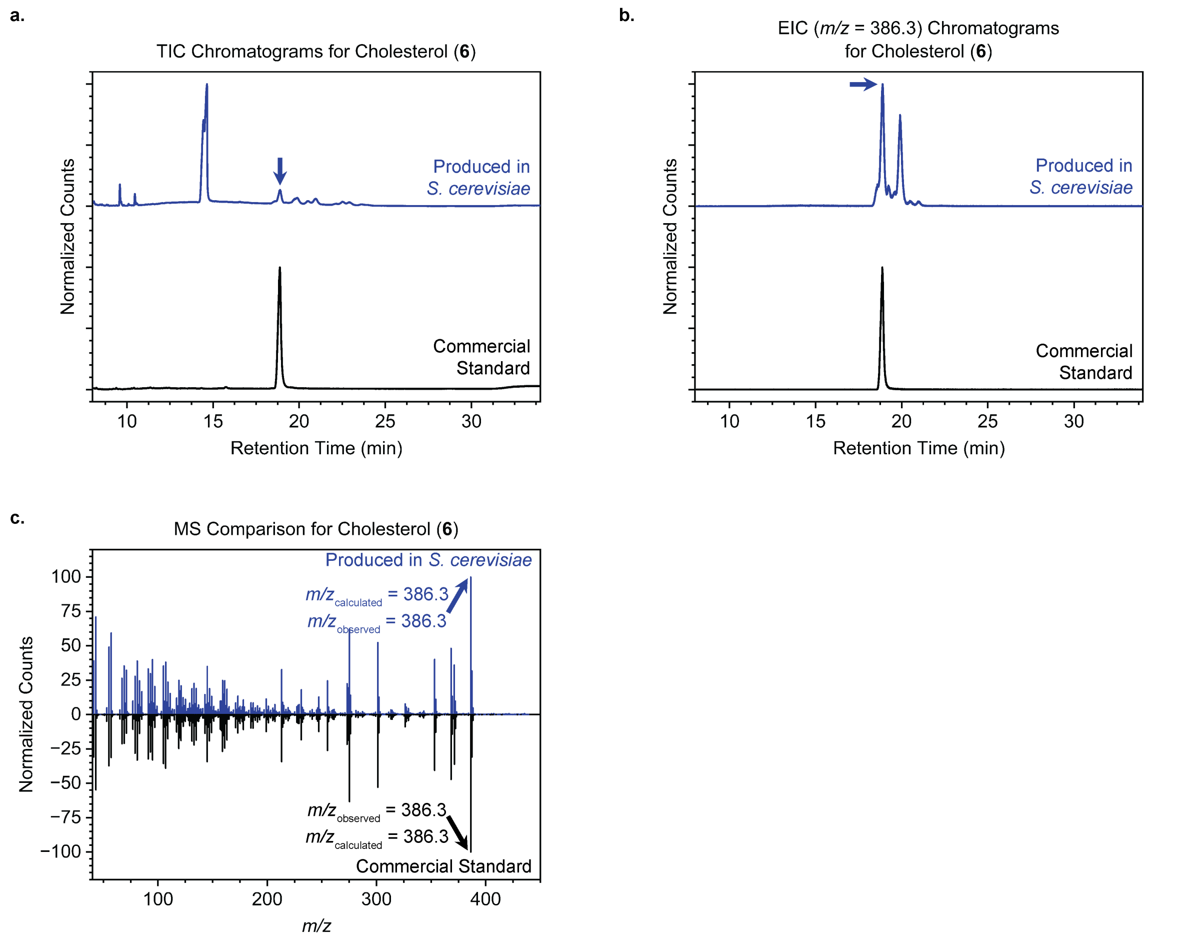

Supplementary Figure S7. a. Total ion chromatograms (TIC), **b.** extracted ion chromatograms (EIC, *m/z* = 386.3), and **c.** mass spectra from GC-MS characterization of cholesterol (**6**) produced in *S. cerevisiae* strain PW-8 (top, blue) and cholesterol (**6**) from a commercial standard (bottom, black).

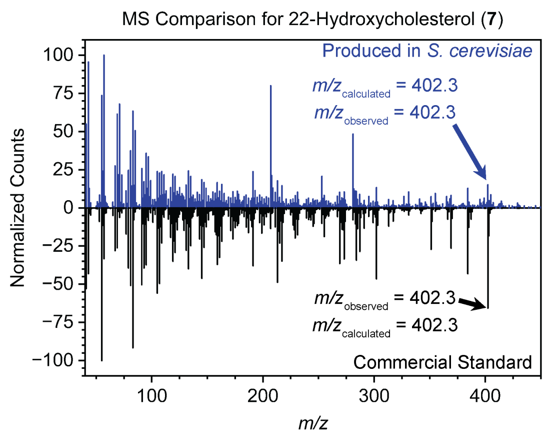

Supplementary Figure S8. a. Mass spectra from GC-MS characterization of 22-hydroxycholesterol (**7**) produced in *S. cerevisiae* strain PW-9 (top, blue) and 22-hydroxycholesterol (**7**) from a commercial standard (bottom, black). The *m/z* = 207.0 peak in the *S. cerevisiae* product (but not the commercial standard) is a contaminant peak on the GC-MS when this data was collected.

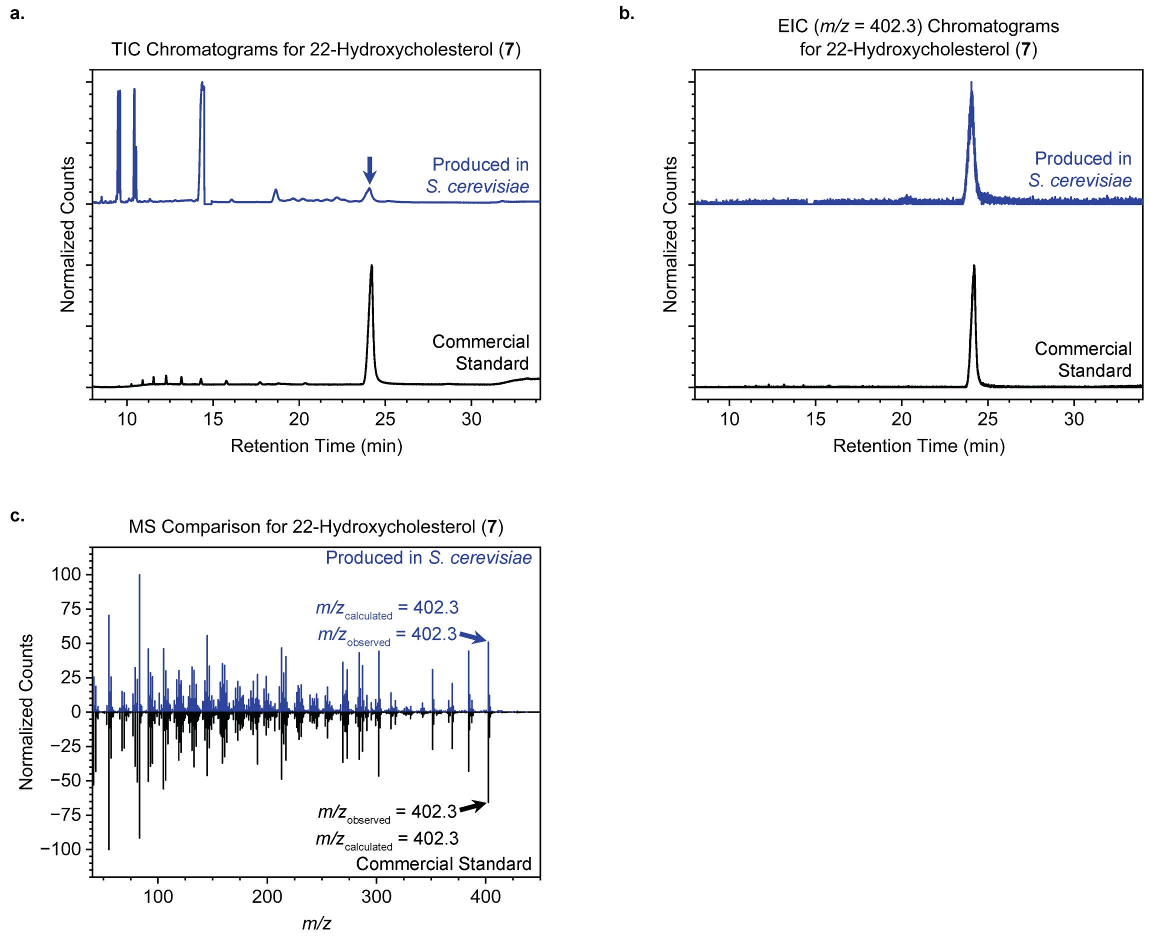

Supplementary Figure S9. a. Total ion chromatograms (TIC), **b.** extracted ion chromatograms (EIC, *m/z* = 402.3), and **c.** mass spectra from GC-MS characterization of 22-hydroxycholesterol (**7**) produced in *S. cerevisiae* strain PW-31 (top, blue) and 22-hydroxycholesterol (**7**) from a commercial standard (bottom, black). There is a gap from 14.5–14.9 min in the TIC chromatogram for *S. cerevisiae* strain PW-31 because the detector was turned off during this time window.

3.5. LC-MS characterization

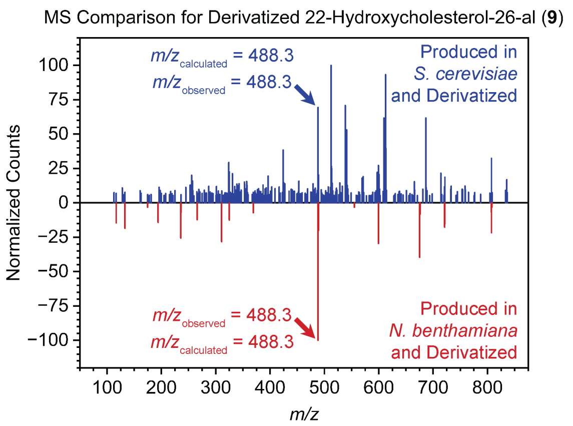

Supplementary Figure S10. a. Mass spectra from LC-MS characterization of 22-hydroxycholesterol-26-al (**8**) produced in *S. cerevisiae* strain PW-32 (top, blue) of 22-hydroxycholesterol-26-al (**8**) produced in *N. benthamiana* transient expression system JR-1 (bottom, red) after both were derivatized to (**9**).

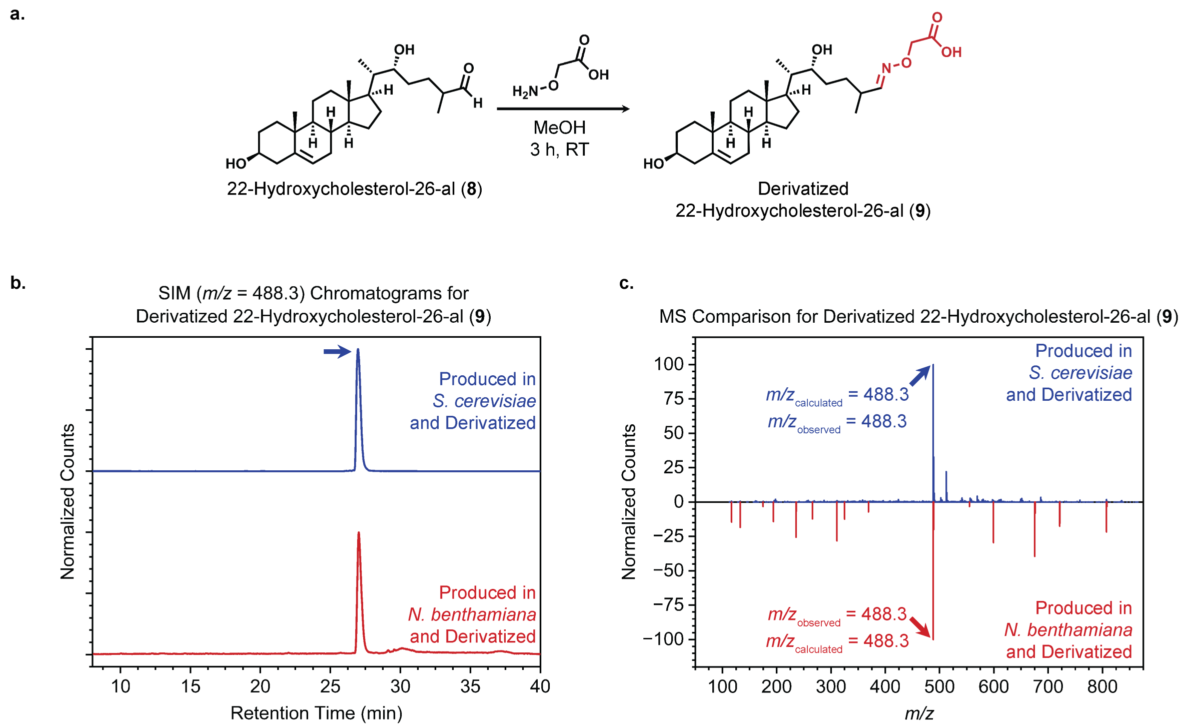

Supplementary Figure S11. a. Scheme for the conversion of 22-hydroxycholesterol-26-al (**8**) into derivatized 22-hydroxycholesterol-26-al (**9**) prior to LC-MS characterization. **b.** Single ion monitoring (SIM) chromatograms (*m/z* = 488.4) and **c.** mass spectra from LC-MS characterization of derivatized 22-hydroxycholesterol-26-al (**9**) for 22-hydroxycholesterol-26-al (**8**) that was produced in *S. cerevisiae* strain PW-33 (top, blue) and *N. benthamiana* transient expression system JR-1 (bottom, red) after both were derivatized to (**9**).

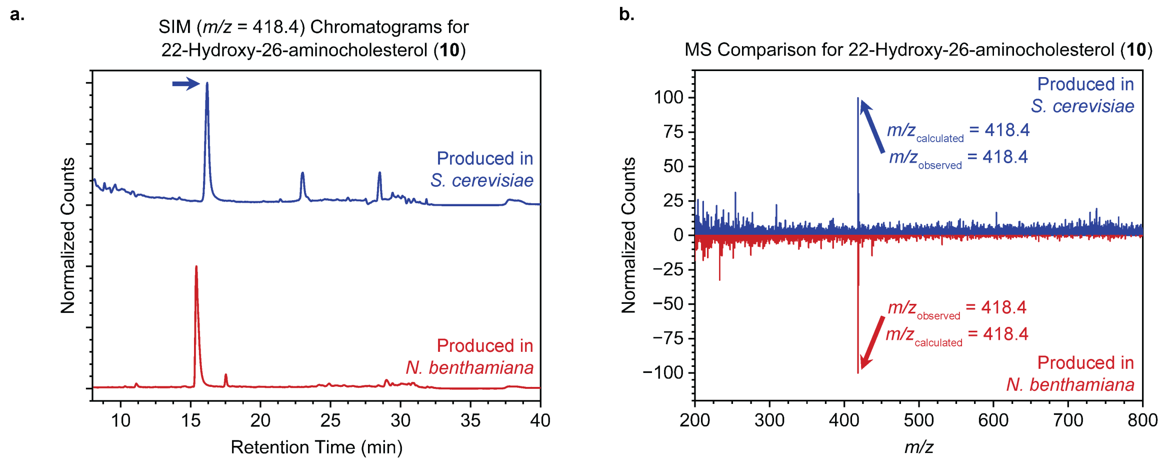

Supplementary Figure S12. a. Single ion monitoring (SIM) chromatograms (*m/z* = 418.4) and **c.** mass spectra from LC-MS characterization of 22-hydroxy-26-aminocholesterol (**10**) that was produced in *S. cerevisiae* strain PW-38 (top, blue) and *N. benthamiana* transient expression system JR-2 (bottom, red).

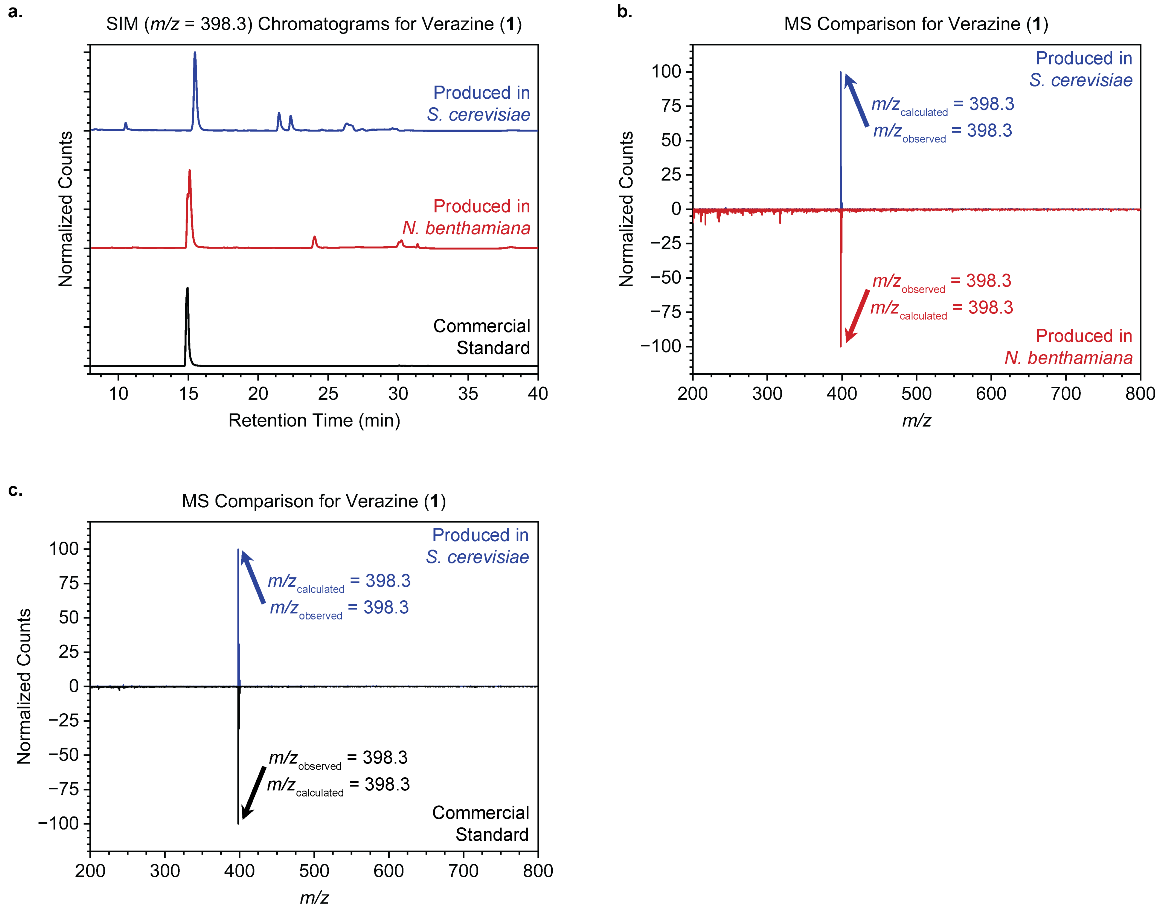

Supplementary Figure S13. a. Single ion monitoring (SIM) chromatograms (*m/z* = 398.3) and **b.-c.** mass spectra from LC-MS characterization of verazine (**1**) that was produced in *S. cerevisiae* strain PW-40 (top, blue), produced in *N. benthamiana* transient expression system JR-3 (bottom, red), and from a commercial standard (bottom, black).

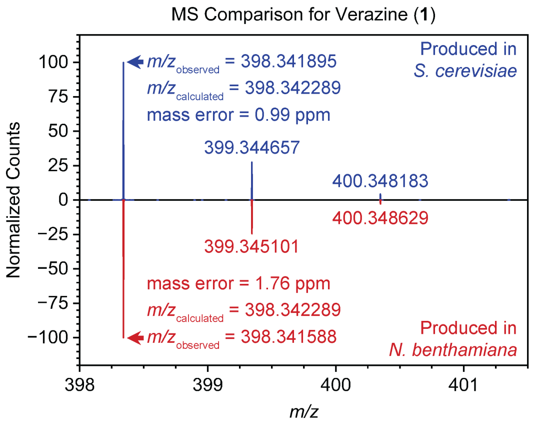

Supplementary Figure S14. High resolution LC-MS characterization for verazine (**1**) produced in *S. cerevisiae* (top, blue) and from a commercial standard (bottom, red).

3.6. DBTL optimization of titers

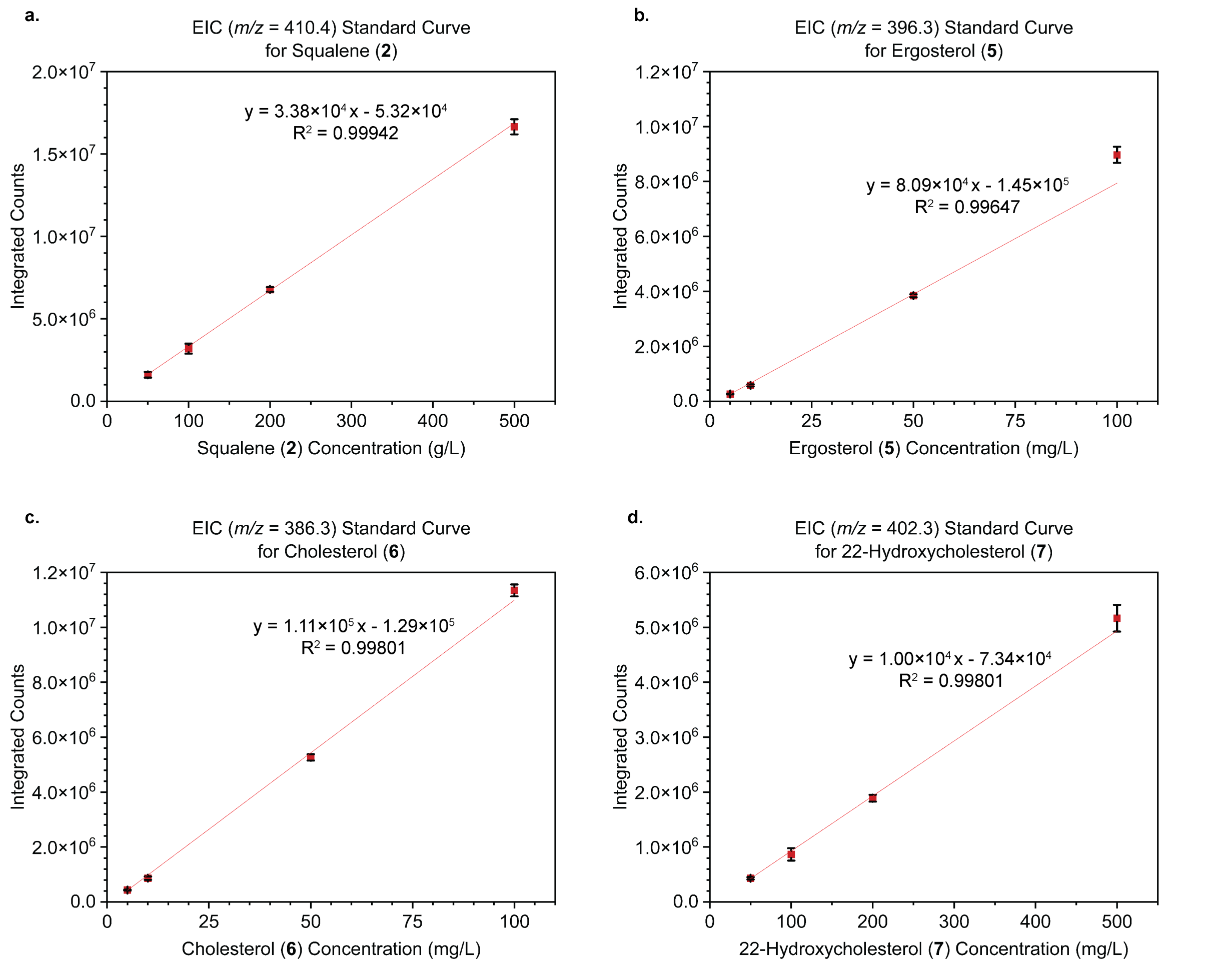

Supplementary Figure S15. Total ion chromatogram (TIC) standard curves from GC-MS characterization for commercial standards of **a.** squalene (**2**), **b.** ergosterol (**5**), **c.** cholesterol (**6**), and **d.** 22-hydroxycholesterol (**7**).

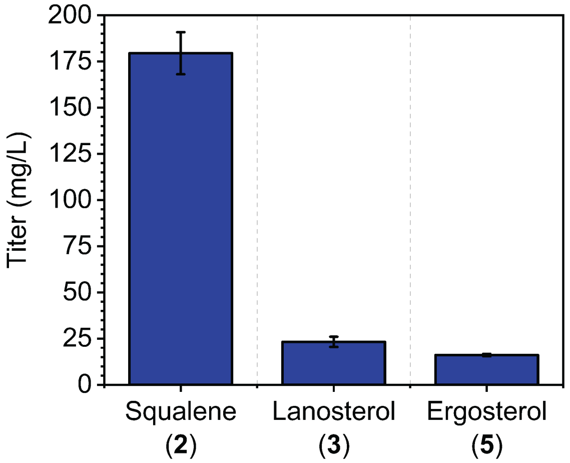

Supplementary Figure S16. Production titers for squalene (**2**), lanosterol (**3**), and ergosterol (**5**) in PW-4 as measured by GC-MS. The standard curve for ergosterol (**5**) was used to estimate the production titers for lanosterol (**3**).

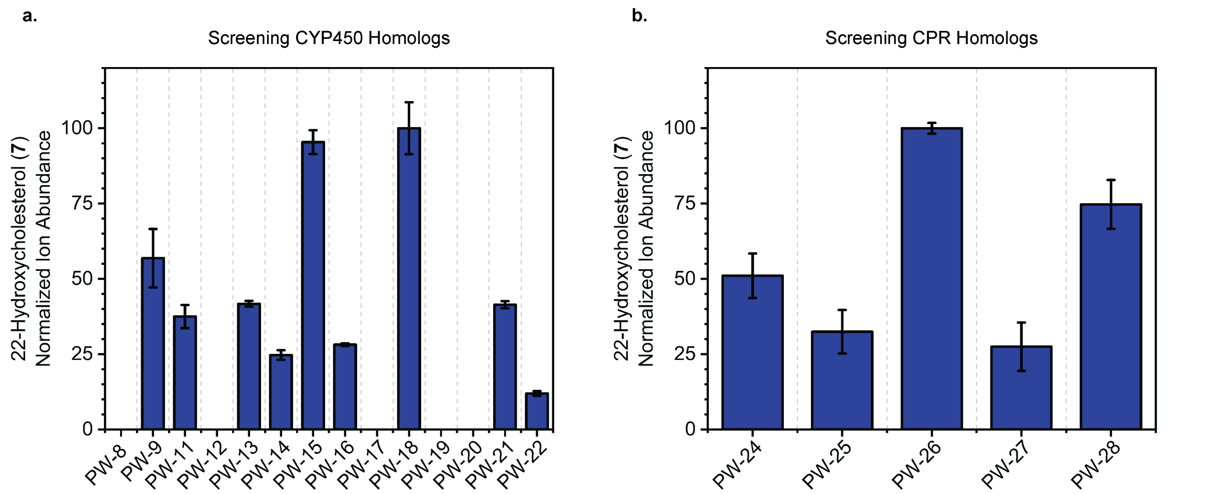

Supplementary Figure S17. Production titers for 22-hydroxycholesterol (**7**) as measured by GC-MS across a homolog screen of **a.** CYP450s and **b.** CPRs.

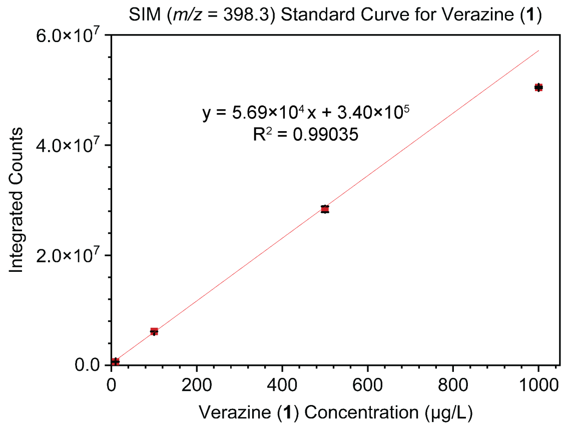

Supplementary Figure S18. Single ion monitoring (SIM) chromatograms (*m/z* = 398.3) standard curve from LC-MS characterization for a commercial standard of verazine (**1**).

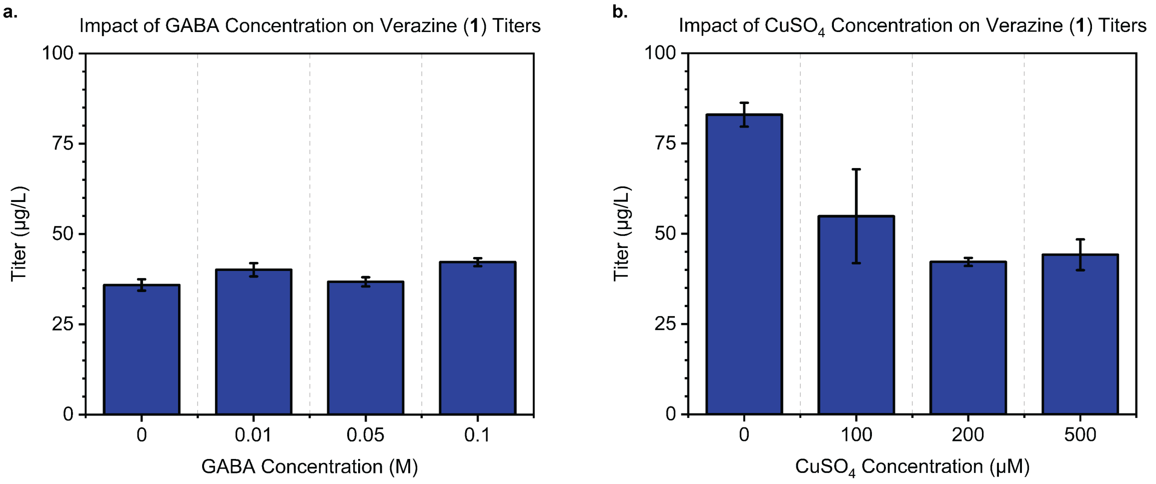

Supplementary Figure S19. The impact of **a.** GABA and **b.** CuSO4 concentration in the production stage medium on verazine (**1**) production was quantified using single ion monitoring (SIM) chromatograms (*m/z* = 398.3) from LC-MS characterization.

a.

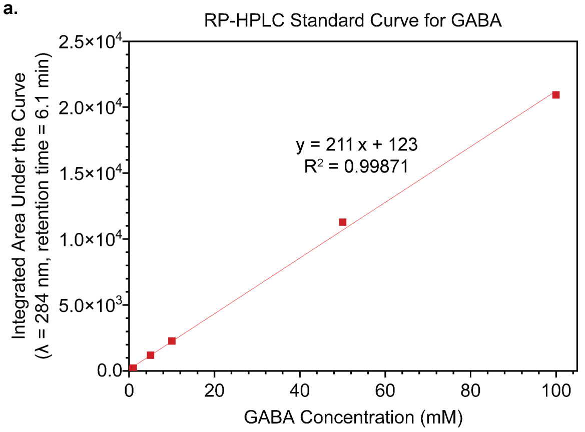

b. GABA Utilization by Yeast Strain PW-42 After 48 h

**Designed Initial GABA Concentration in the Production Medium (mM)**	**Quantified Final GABA Concentration in the Medium After the Production Stage (mM)**
100	105 ± 4
50	55 ± 1
10	9.7 ± 0.2
0	0

Supplementary Figure S20. a. Integrated area under the curve (λ = 284 nm, retention time = 6.1 min) standard curve from reverse-phase high-performance liquid chromatography (RP-HPLC) characterization for GABA derivatized with diethyl ethoxymethylenemalonate (DEEMM). **b.** GABA utilization by yeast strain PW-42 was quantified after a 48 h production stage. GABA in the medium after the production stage was derivatized by DEEMM and quantified by RP-HPLC.

3.7. Confocal microscopy

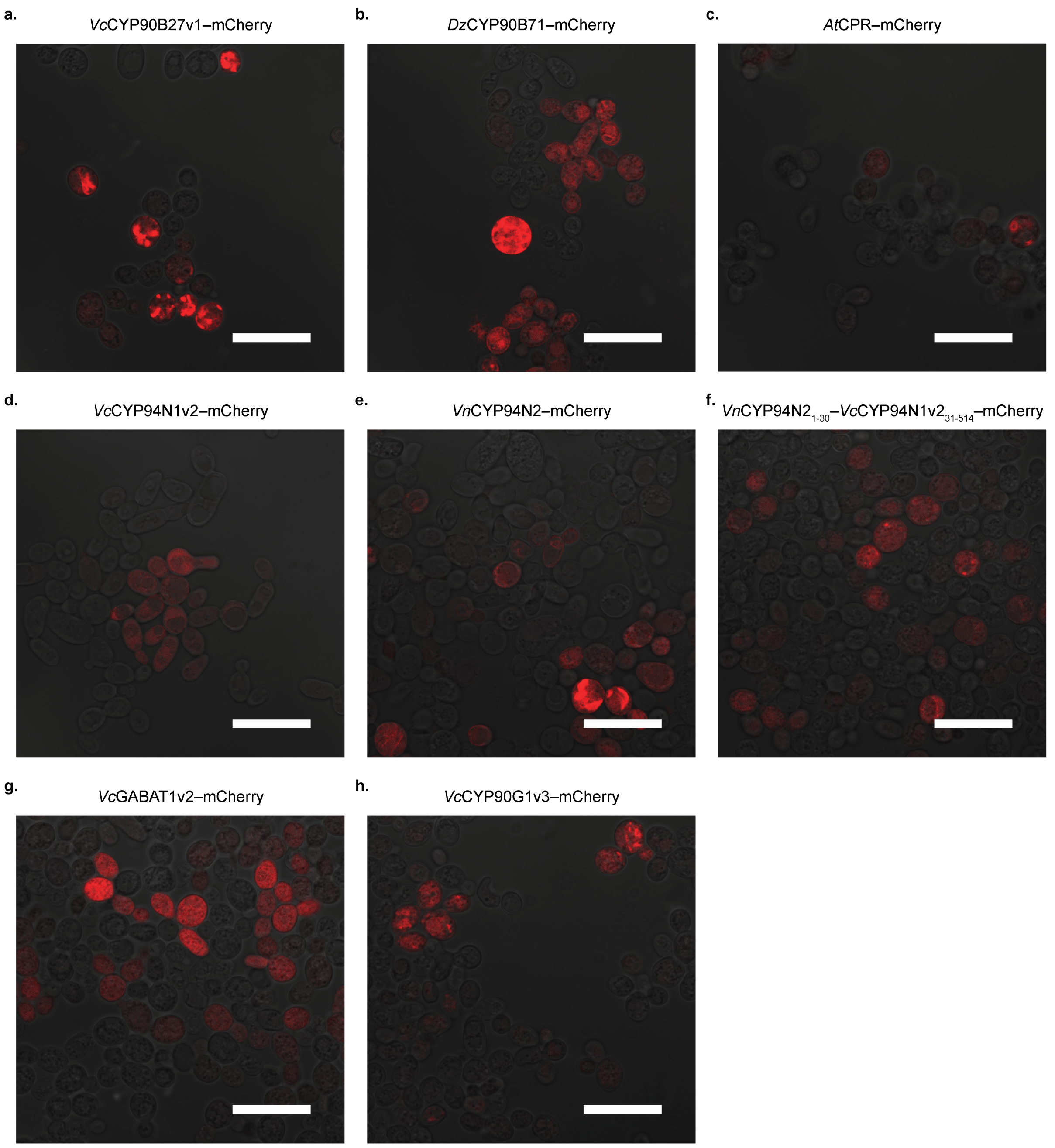

Supplementary Figure S21. Confocal microscopy images characterizing the localization of **a.** *Vc*CYP90B27v1–mCherry (strain PW-10), **b.** *Dz*CYP90B71–mCherry (strain PW-23), **c.** *At*CPR–mCherry (strain PW-29), **d.** *Vc*CYP94N1v2–mCherry (strain PW-35), **e.** *Vn*CYP94N2–mCherry (strain PW-36), **f.** *Vn*CYP94N2$_{1-30}$–*Vc*CYP94N1v2$_{31-514}$–mCherry (strain PW-37), **g.** *Vc*GABAT1v2–mCherry (strain PW-39), and **h.** *Vc*CYP90G1v3–mCherry (strain PW-41). These images are a combination of mCherry fluorescence and brightfield transmission images. All scale bars are 20 μm.

3.8. Proteomics

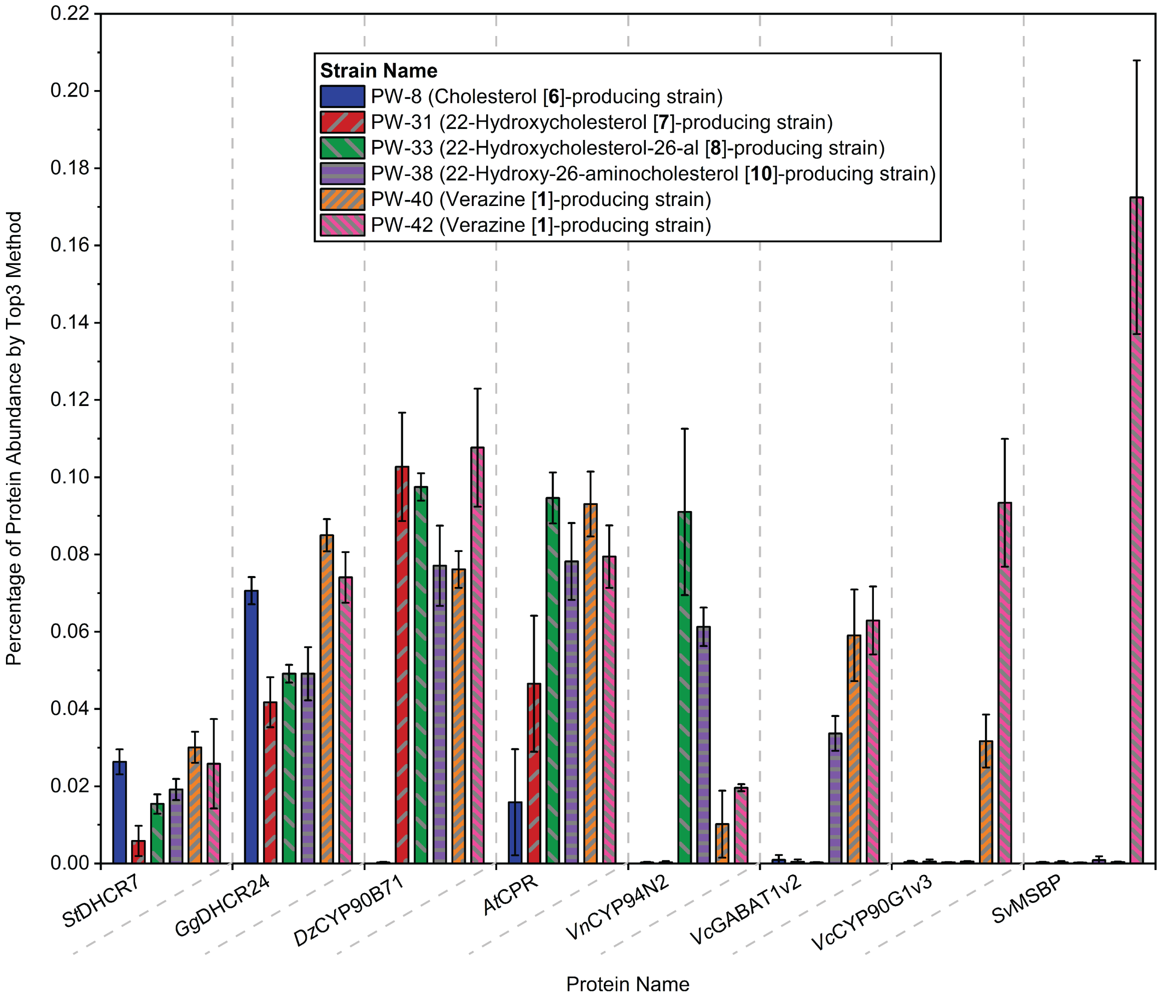

Supplementary Figure S22. Expression levels for each heterologous protein were quantified in *S. cerevisiae* strains using LC-MS-based proteomics characterization via the Top3 method.

4. REFERENCES

(1) Liu, Y.; Zhao, X.; Gan, F.; Chen, X.; Deng, K.; Crowe, S. A.; Hudson, G. A.; Belcher, M. S.; Schmidt, M.; Astolfi, M. C. T.; Kosina, S. M.; Pang, B.; Shao, M.; Yin, J.; Sirirungruang, S.; Iavarone, A. T.; Reed, J.; Martin, L. B. B.; El-Demerdash, A.; Kikuchi, S.; Misra, R. C.; Liang, X.; Cronce, M. J.; Chen, X.; Zhan, C.; Kakumanu, R.; Baidoo, E. E. K.; Chen, Y.; Petzold, C. J.; Northen, T. R.; Osbourn, A.; Scheller, H.; Keasling, J. D. Complete Biosynthesis of QS-21 in Engineered Yeast. *Nature* **2024**, *629*, 937–944. https://doi.org/10.1038/s41586-024-07345-9.

(2) Reider Apel, A.; d'Espaux, L.; Wehrs, M.; Sachs, D.; Li, R. A.; Tong, G. J.; Garber, M.; Nnadi, O.; Zhuang, W.; Hillson, N. J.; Keasling, J. D.; Mukhopadhyay, A. A Cas9-Based Toolkit to Program Gene Expression in Saccharomyces Cerevisiae. *Nucleic Acids Res.* **2017**, *45* (1), 496–508. https://doi.org/10.1093/nar/gkw1023.

(3) Entian, K. D.; Kötter, P. Entian KD and Kötter P Yeast Genetic Strain and Plasmid Collections. *Yeast Genetic Strain and Plasmid Collections. Methods in Microbiology* **2007**, *36*, 629–666.

(4) Wong, J.; d'Espaux, L.; Dev, I.; van der Horst, C.; Keasling, J. De Novo Synthesis of the Sedative Valerenic Acid in Saccharomyces Cerevisiae. *Metab. Eng.* **2018**, *47*, 94–101. https://doi.org/10.1016/j.ymben.2018.03.005.

(5) Augustin, M. M.; Ruzicka, D. R.; Shukla, A. K.; Augustin, J. M.; Starks, C. M.; O'Neil-Johnson, M.; McKain, M. R.; Evans, B. S.; Barrett, M. D.; Smithson, A.; Wong, G. K.-S.; Deyholos, M. K.; Edger, P. P.; Pires, J. C.; Leebens-Mack, J. H.; Mann, D. A.; Kutchan, T. M. Elucidating Steroid Alkaloid Biosynthesis in Veratrum Californicum: Production of Verazine in Sf9 Cells. *Plant J.* **2015**, *82* (6), 991–1003. https://doi.org/10.1111/tpj.12871.

(6) Kou, C.; Liu, J.; Yin, X.; He, D.; Liu, J.; Hua, X.; Ma, R.; Sun, W.; Xue, Z.; Ma, P. Efficient Heterologous Biosynthesis of Verazine, a Metabolic Precursor of the Anti-Cancer Drug Cyclopamine. *Plant Commun.* **2024**, *5*, 100831. https://doi.org/10.1016/j.xplc.2024.100831.

(7) Bhattacharya, S.; Esquivel, B. D.; White, T. C. Overexpression or Deletion of Ergosterol Biosynthesis Genes Alters Doubling Time, Response to Stress Agents, and Drug Susceptibility in Saccharomyces Cerevisiae. *MBio* **2018**, *9* (4). https://doi.org/10.1128/mBio.01291-18.

(8) Souza, C. M.; Schwabe, T. M. E.; Pichler, H.; Ploier, B.; Leitner, E.; Guan, X. L.; Wenk, M. R.; Riezman, I.; Riezman, H. A Stable Yeast Strain Efficiently Producing Cholesterol Instead of Ergosterol Is Functional for Tryptophan Uptake, but Not Weak Organic Acid Resistance. *Metab. Eng.* **2011**, *13* (5), 555–569. https://doi.org/10.1016/j.ymben.2011.06.006.
